# Strain induced reactivity of cyclic iminoboranes: the (2 + 2) cycloaddition of a 1*H*-1,3,2-diazaborepine with ethene†

**DOI:** 10.1039/d3sc04901a

**Published:** 2023-12-12

**Authors:** Divanshu Gupta, Ralf Einholz, Holger F. Bettinger

**Affiliations:** a Institut für Organische Chemie, Universität Tübingen Auf der Morgenstelle 18 72076 Tübingen Germany holger.bettinger@uni-tuebingen.de

## Abstract

Iminoboranes have gathered immense attention due to their reactivity and potential applications as isoelectronic and isosteric alkynes. While cyclic alkynes are well investigated and useful reagents, cyclic iminoboranes are underexplored and their existence was inferred only *via* trapping experiments. We report the first direct spectroscopic evidence of a cyclic seven-membered iminoborane, 1-(*tert*-butyldimethylsilyl)-1*H*-1,3,2-diazaborepine 2, under cryogenic matrix isolation conditions. The amino-iminoborane 2 was photochemically generated in solid argon at 4 K from 2-azido-1-(*tert*-butyldimethylsilyl)-1,2-dihydro-1,2-azaborinine (3) and was characterized using FT-IR, UV-vis spectroscopy, and computational chemistry. The characteristic BN stretching vibration (1751 cm^−1^) is shifted by about 240 cm^−1^ compared to linear amino-iminoboranes indicating a significant weakening of the bond. The Lewis acidity value determined computationally (LA_B_ = 9.1 ± 2.6) is similar to that of boron trichloride, and twelve orders of magnitude lower than that of 1,2-azaborinine (BN-aryne, LA_B_ = 21.5 ± 2.6), a six-membered cyclic iminoborane. In contrast to the latter, the reduced ring strain of 2 precludes nitrogen fixation, but it unexpectedly allows facile (2 + 2) cycloaddition reaction with C_2_H_4_ under matrix isolation conditions at 30 K.

## Abstract 2

Cyclic seven-membered iminoborane, 1-(*tert*-butyldimethylsilyl)-1*H*-1,3,2-diazaborepine 2, generated in cryogenic conditions which undergoes facile (2 + 2) cycloaddition reaction with C_2_H_4_ at 30 K.

## Graphical abstract

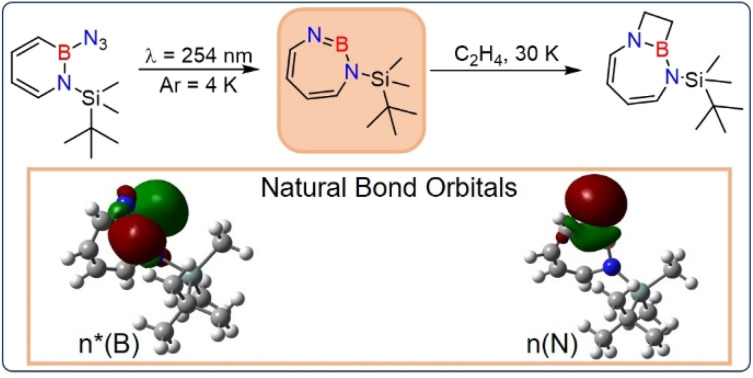

## Introduction

**Scheme 1:**
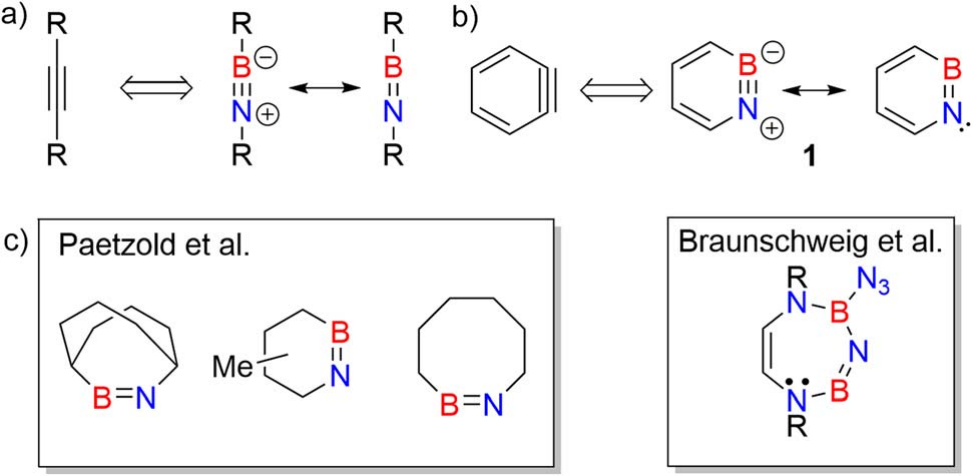
(a) Iminoboranes and their analogy with alkynes. (b) Resonance forms of 1,2-azaborinine (1) and its analogy with *ortho*-benzyne. (c) Representative examples of cyclic iminoboranes previously inferred as reactive intermediates by Paetzold *et al.*^61^ and Braunschweig *et al.*^42^

Iminoboranes are an important class of BN containing compounds.^1–3^ The BN/CC isosterism relates iminoboranes and alkynes (Scheme 1a).^4^ In contrast to the latter, iminoboranes are kinetically unstable towards cyclooligomerization or polymerization and thus special conditions or steric protection are required for their synthesis and isolation.^1,2,5–12^ The first iminoborane stable at room temperature, F_5_C_6_-BN-*t*Bu, was reported by Paetzold *et al.*,^11^ and followed by numerous examples.^2,9,13–15^ Since then, iminoboranes have attracted tremendous attention.^16–23^ Various iminoboranes have been investigated over the past decades to understand their reactivity^24–27^ including the formation of BN-doped polycyclic aromatic hydrocarbons (PAHs),^28–32^ formation of N-heterocyclic carbene coordinated iminoboranes,^25,33–36^ synthesis of BN containing heterocycles,^37–44^ (2 + 2) and (2 + 3) cycloaddition reactions with a number of polar double bonds (*e.g.*, RR′C=O) and dipolar reagents,^45–50^ formation of iminoboryl carboranes,^19,51^ and use of frustrated Lewis pairs to stabilize iminoboranes.^19,52–57^ A series of *ab initio* computational studies performed by Gilbert compared iminoboranes and alkynes with respect to the electronic and geometric structure, as well as the reactivity in (2 + 2) and (2 + 4) cycloadditions towards alkenes and alkynes.^58–60^

Studies on cyclic iminoboranes are quite rare.^42,61^ Due to ring strain, they are more reactive than linear iminoboranes,^1^ but coordination by N-heterocyclic carbenes (NHC) provides a way of stabilization.^36,62,63^ A special case of cyclic iminoboranes is the aromatic BN-aryne, 1,2-azaborinine 1, the BN analogue of *ortho*-benzyne (Scheme 1b).^64–66^ This was detected by matrix isolation methods by our group and shows remarkably high reactivity towards inert molecules.^64–66^ The polarity of the strained BN link of 1 results in a bonding situation that differs from that of the strained triple bond in arynes and from that of the BN unit in linear iminoboranes.^64,66,67^ The dibenzo derivative of 1, dibenzo[*c*,*e*][1,2]azaborinine, was inferred as reactive intermediate in solution,^68–70^ and can even activate the strong Si–F bond for subsequent insertion reaction.^70^

**Scheme 2:**
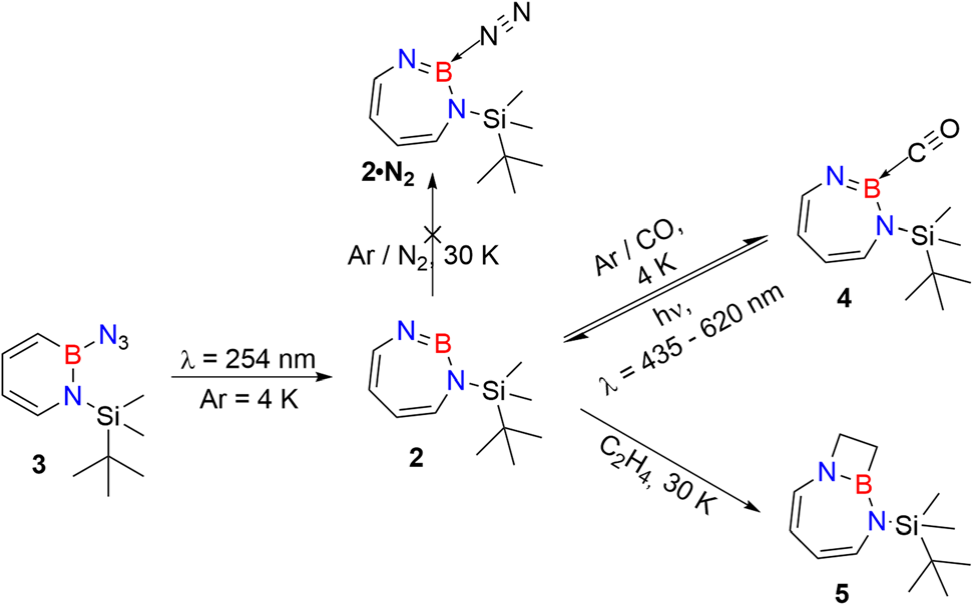
Photogeneration of 1-(*tert*-butyldimethylsilyl)-1,3,2-diazaborepine 2 under matrix isolation conditions and its reaction with CO and C_2_H_4_.

As iminoborane units in larger than six-membered rings have never been observed directly, but only inferred from trapping experiments (Scheme 1c),^42,61^ we studied the seven membered 1-(*tert*-butyldimethylsilyl)-1*H*-1,3,2-diazaborepine 2 to elucidate the impact of ring size on the reactivity of cyclic iminoboranes. The matrix isolation technique is ideally suited to study such highly reactive intermediates directly, and furthermore probe their reactivity towards interesting compounds. We generated target compound 2 in solid argon by the photolysis of 2-azido-1-(*tert*-butyldimethylsilyl)-1,2-dihydro-1,2-azaborinine (3) under cryogenic matrix conditions (see Scheme 2). On one hand, ring strain will be reduced in this seven-membered ring iminoborane compared to 1 and this is expected to reduce its reactivity, while on the other hand, the eight π-electron count may induce some degree of antiaromaticity that will potentially destabilize 2. Seven-membered cyclic iminoboranes were never directly observed, but recently inferred as reactive intermediate in the formation of diazadiboretidines by Braunschweig and co-workers.^42^ We provide here for the first time direct spectroscopic evidence for a cyclic seven-ring iminoborane and reveal its unexpectedly facile (2 + 2) cycloaddition reaction (Scheme 2).

## Results and discussion

### Generation and characterization of cyclic iminoborane 2

**Fig. 1:**
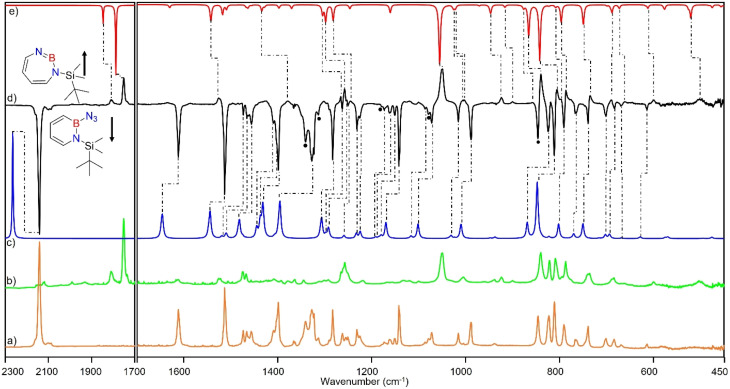
(a) IR spectrum obtained after deposition of 3 in Ar matrix at 28 K. (b) IR spectrum obtained after irradiation of Ar matrix with *λ* = 254 nm (following the deposition of 3). (c) Spectrum for ^11^B and ^10^B isotopologues (81 : 19) of 3 calculated at the B3LYP-D3(BJ)/6-311+G(d,p) level of theory. (d) Difference spectrum obtained after irradiation of Ar matrix with *λ* = 254 nm (following the deposition of 3). (e) Spectrum for ^11^B and ^10^B isotopologues (81 : 19) of 2 calculated at the B3LYP-D3(BJ)/6-311+G(d,p) level of theory (● corresponds to the overtones and combination bands according to computed anharmonic vibrational frequency analysis).

Matrix-isolation infrared spectroscopic studies of 3 were performed in solid Ar at 4 K. The most distinguished peak at 2141 cm^−1^ corresponds to the azide stretching vibration (Fig. 1, S9, S10 and Table S13 in ESI†). Irradiation of matrix isolated precursor 3 with *λ* = 254 nm until it was completely bleached, as shown in the difference spectrum (Fig. 1d), resulted in a new set of IR bands. This set is assigned to 2 based on comparison with computational data obtained at the B3LYP-D3(BJ)/6-311+G(d,p) level of theory (Fig. 1e). The formation of the structural isomer 1*H*-1,2,3-diazaborepine or the triplet boryl nitrene, the expected primary product of N_2_ extrusion from 3, can be excluded based on the computations (see Fig. S15†). Among the new bands formed during photolysis of 3, the most intense peak, 1751 cm^−1^ corresponds to the BN stretching vibration of 2 (Table S15†) by comparison with the computations (Fig. 1e). The band at 1809 cm^−1^ corresponds to the BN stretching vibration of the ^10^B isotopologue, and the isotopic shift of 58 cm^−1^ is in good agreement with computations (60 cm^−1^). The formation of the 1*H*-1,3,2-diazaborepine isomer is in agreement with the known behavior of azidoboranes: thermolysis or photolysis of diorganyl azidoboranes results in iminoboranes without trappable borylnitrenes,^1^ while only in the case of donor substitution (X = NR_2_, OR) the borylnitrenes X_2_BN could be trapped or directly observed.^71–77^ The photolysis of azide 3 is thus expected to result in ring enlargement by shift of the carbon rather than the nitrogen center to give the cyclic iminoborane 2.

For linear amino-iminoboranes, the BN stretching mode was reported around 1990 cm^−1^ and 2030 cm^−1^ for the ^11^B and ^10^B isotopologues, respectively.^78,79^ The large shift of the BN stretching frequency of 2 from linear diorganyl substituted amino-iminoboranes of around 240 cm^−1^ is due to the cyclic nature of 2 which results in considerable weakening of the BN bond. Note that the BN stretching for 1 at 1637/1634 cm^−1^ indicates an even weaker BN bond in the six-membered ring.^64^

**Fig. 2:**
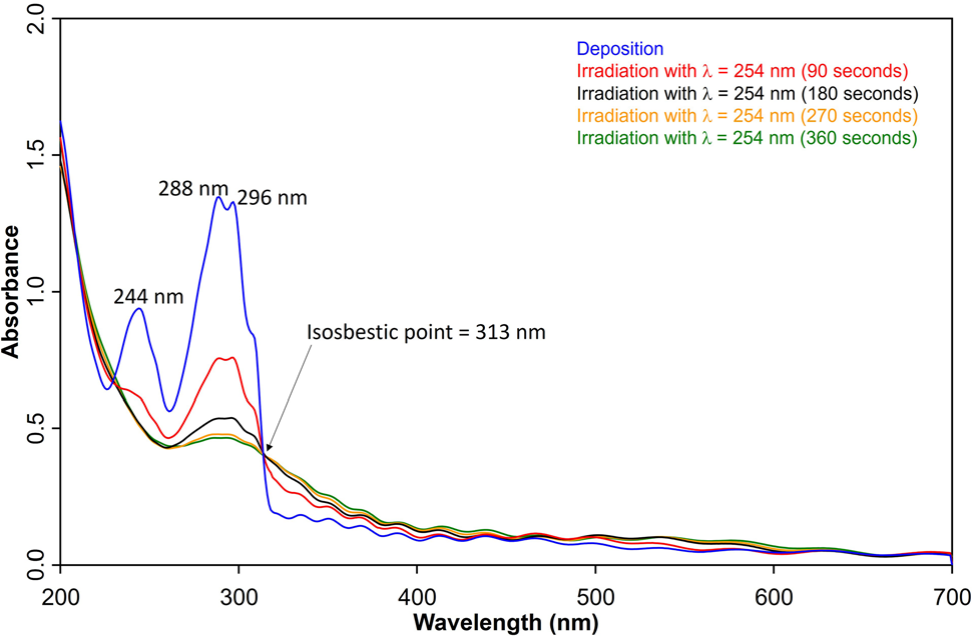
UV-vis spectra of 3 (blue: after deposition at 28 K, *λ*_max_ = 288 nm) and subsequent formation 2 (*λ*_max_ = 308 nm) after various irradiation (*λ* = 254 nm) steps under matrix isolation conditions.

UV/vis spectroscopy (Fig. 2) of the azide 3 in Ar at 8 K shows a strong feature around 290 nm with fine structure and maxima at 296 nm, 288 nm, and 244 nm that is typical for 1,2-dihydro-1,2-azaborinines.^80,81^ Irradiation with *λ* = 254 nm results in quick decrease of the azide absorption band with an isosbestic point at 313 nm. The photoproduct 2 only has a relatively weaker and broad absorption maximum centered at 305 nm. The measured spectra of 3 and 2 are in good agreement with TD-CAM-B3LYP/6-311+G(d,p) computations (Fig. S8†).

**Fig. 3:**
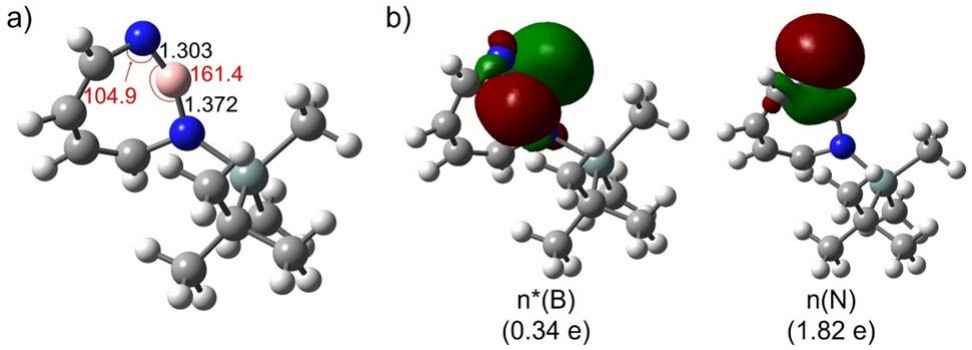
(a) Geometrical parameters of 2. (b) Natural bond orbitals (NBOs) and their occupation numbers of 2. Important bond lengths [Å] and bond angles [°] are given. All computations at the M06-2X/6-311+G(d,p) level of theory.

The most remarkable feature of the computed geometry (M06-2X/6-311+G(d,p)) of 2 is the distortion of the heptagon with a small angle of 104.9° at dicoordinated nitrogen and a large angle of 161.4° at boron in the singlet ground state (Fig. 3a). Similarly to the case of 1,2-azaborinine,^64^ this feature can be rationalized by the difference in electronegativity: the more electronegative N atom prefers to have a σ-type lone pair (HOMO-1), while the more electropositive B atom prefers an empty σ-type orbital (LUMO) which has a strong p character (Fig. 3b). The natural bond orbital (NBO) analysis at the M06-2X/6-311+G(d,p) level of theory arrives at occupancies of 1.82e^−^ and 0.34e^−^ for the lone pair and empty orbitals, respectively. There is a pronounced n(N) → n*(B) interaction [*E*(2) = 34.5 kcal mol^−1^] according to the second-order perturbation estimate of the donor–acceptor interaction in the NBO basis that is smaller than that in 1 (39.7 kcal mol^−1^). The natural charges obtained from the NBO analysis on N and B are large (−0.91 on N and +1.16 on B) while the Wiberg bond index between B and N is only 1.55. Compared to 1,2-azaborinine 1 the Wiberg bond index is larger (1.43 in 1) and the polarity of the BN bond (natural charges in 1: −0.81 on N and +1.00 on B; NBO occupancies of 1.82e^−^ and 0.21e^−^ for the lone pair and empty orbitals) is slightly increased in 2.

**Scheme 3:**
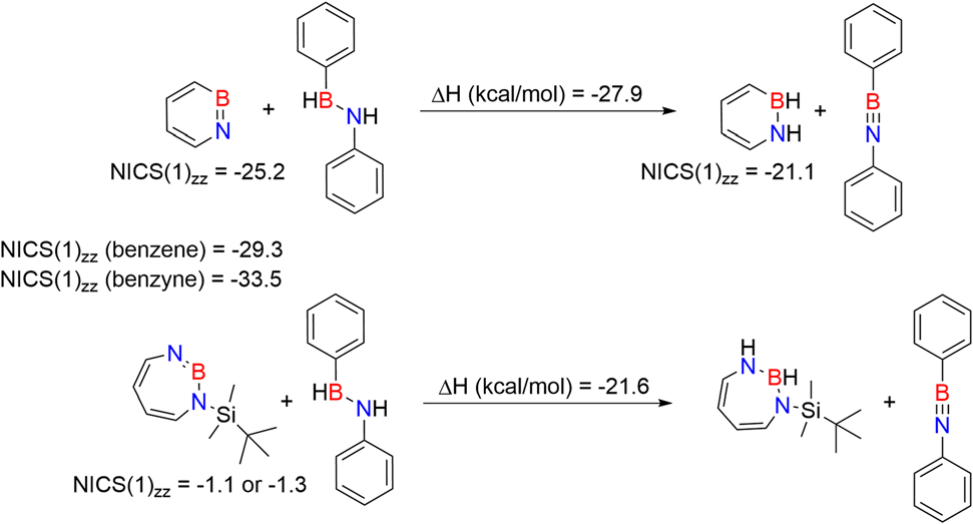
NICS(1)_*zz*_ values and homodesmotic reactions for the calculation of strain energy.

We compared the aromatic character of 2 with that of 1, benzene, and benzyne using nuclear independent chemical shift (NICS) calculations,^82–84^ placing the dummy atom 1 Å above the center of the ring to get the NICS(1)_*zz*_ value. 1,2-Azaborinine 1 has a NICS(1)_*zz*_ value of −25.2 which is lower than that of benzene (−29.3) and benzyne (−33.5), but still suggests aromatic character that is slightly larger than that of 1,2-dihydro-1,2-azaborine (NICS(1)_*zz*_ = −21.1) (Scheme 3).^85^ The NICS(1)_*zz*_ value of −1.1 and −3.1 computed for 2 is significantly lower suggesting that the antiaromaticity expected on the basis of the formal electron count is not relevant (see Fig. S17†). The two slightly differing values for 2 are due to the non-planar ring. The NICS(1)_*zz*_ value of antiaromatic *D*_2h_ cyclobutadiene is +10.9 computed at the same level of theory.

**Scheme 4:**
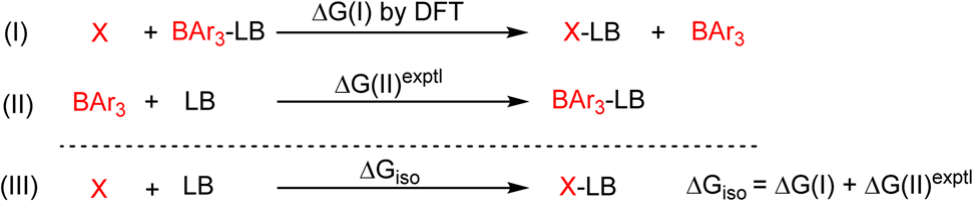
Combining the isodesmic reaction [eqn (I)] with an experimental reference reaction [eqn (II)] allows one to determine the Lewis acidities of X (X= 1 or 2) from Δ*G*_iso_ [eqn (III)]. The Lewis bases pyridine, acetonitrile, and benzaldehyde and the Lewis acids BAr_3_ (Ar = C_6_H_5_, 4-ClC_6_H_4_, 3,4,5-F_3_C_6_H_2_) were chosen as references. The borane Lewis acidity scale is defined so that LA_B_(triphenylborane) = 0 following Ofial *et al.*^86^

We compared the strain of the two cyclic iminoboranes 1 and 2 by considering homodesmotic reactions (Scheme 3). As expected, the strain energy of the seven-membered ring iminoborane 2 is lower than that of 1 by approximately 6.3 kcal mol^−1^ at the M06-2X/6-311+G(d,p) level of theory. We also computed the Lewis acidities (LA_B_) of 1 and 2 using the method of Ofial *et al.* from known Lewis basicities LB_B_ and Lewis acidities LA_B_, computed equilibrium constants (Scheme 4, 
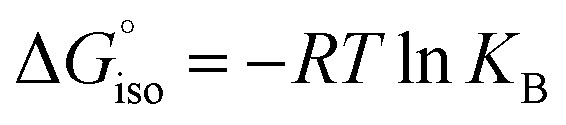
), and the equation log *K*_B_ = LA_B_ + LB_B_.^86^ Ofial *et al.*^86^ obtained the LA_B_ parameters from Δ*G*_iso_ to give LA_B_ = 8.4 ± 2.0 for BF_3_, LA_B_ = 9.3 ± 1.8 for BCl_3_, and LA_B_ = 10.1 ± 1.3 for BBr_3_. The qualitative ordering obtained by Ofial *et al.*^86^ of Lewis acidities LA_B_ with BF_3_ < BCl_3_ < BBr_3_ is in accordance with Lewis acidity rankings based on spectroscopic data.^87–89^ The individual LA_B_ parameters obtained from Δ*G*_iso_ for three different reference bases were averaged to give LA_B_ = 21.5 ± 2.6 for 1 and LA_B_ = 9.1 ± 2.6 for 2 (see the ESI† for further details). While the Lewis acidity of 2 is thus similar to that of BCl_3_, that of 1 is 12 orders of magnitudes larger.

### Interaction of 2 with N_2_

**Fig. 4:**
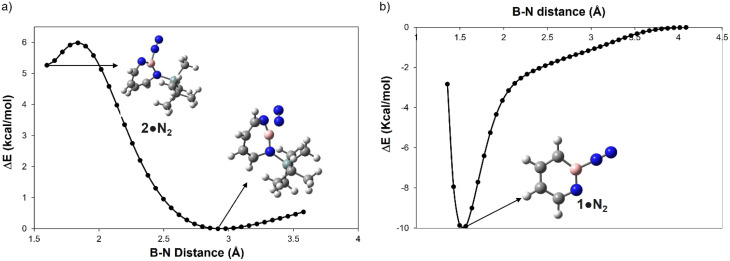
Plot for the relative energy in kcal mol^−1^ as a function of the boron N_2_ distance in Å for (a) 2 and (b) 1 computed at B3LYP-D3(BJ)/6-311+G(d,p) level of theory.

A hallmark of the superelectrophilic 1,2-azaborinine 1 is its ability to bind nitrogen to give adduct 1·N_2_ under cryogenic conditions.^64^ In order to study the Lewis acidity of the photogenerated 1*H*-1,3,2-diazaborepine 2 experimentally, the matrix was annealed up to 35 K. This did not lead to any further spectral changes, indicating that 2 cannot bind the photochemically extruded N_2_ that is lying in its vicinity. The potential energy paths computed with the B–N_2_ distance as parameter (B3LYP-D3(BJ)/6-311+G(d,p)) reveal that formation of the dative complex 2·N_2_ is energetically unfavorable and involves a barrier (Fig. 4a). In contrast, formation of 1·N_2_ is without barrier and exothermic (Fig. 4b).

### Interaction of 2 with CO

**Fig. 5:**
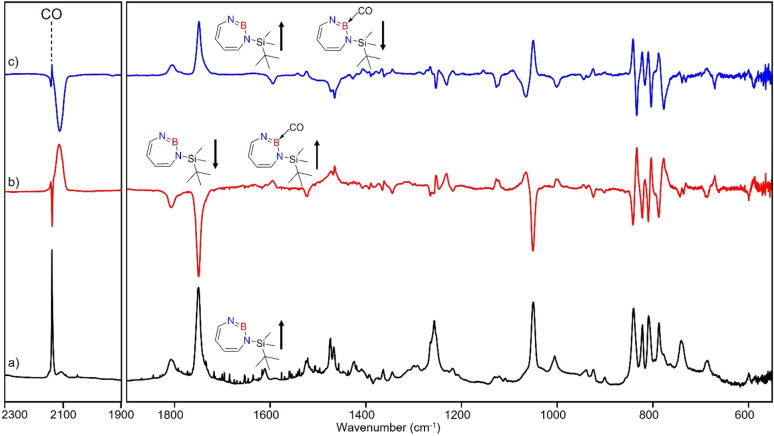
Infrared spectra obtained after irradiation of 3 in CO (2–3%) doped argon matrix. (a) After 60 min irradiation with *λ* = 254 nm at *T* = 4 K. (b) Difference spectrum after annealing for 30 min at 30 K (following the irradiation with *λ* = 254 nm). (c) Difference spectrum after irradiation with 435 nm > *λ* > 620 nm for 30 min (following the annealing at 30 K).

The stronger Lewis base CO can undergo formation of adduct 4 as revealed in experiments using mixtures of Ar and CO (1–2% in Ar) (Scheme 2 and Fig. 5). In separate experiments, the isotopologues ^13^CO and C^18^O (Fig. S11–S13†) were employed in order to obtain isotopic shifts of vibrational bands for comparison with computations.

**Fig. 6:**
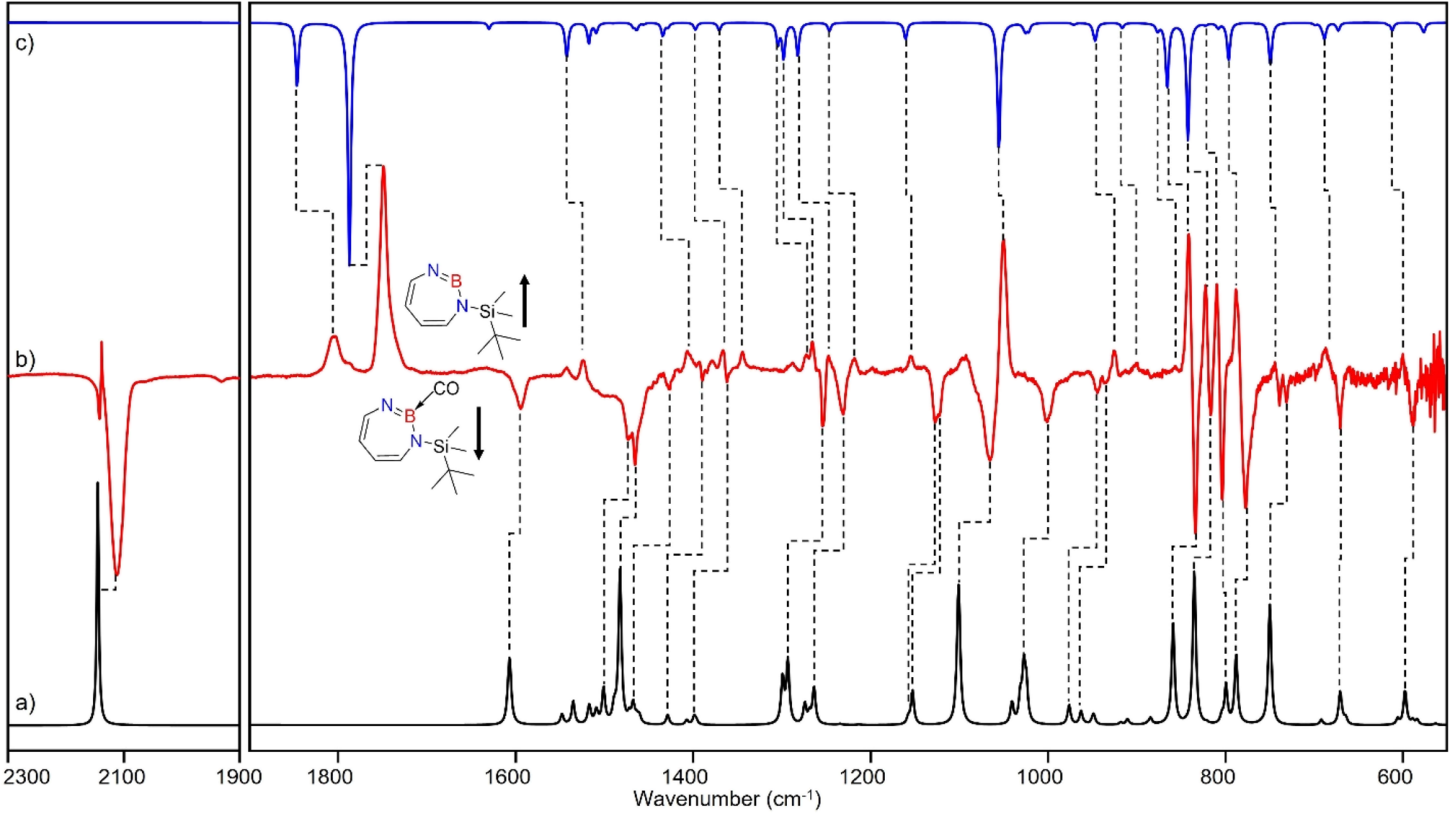
(a) Spectrum for ^11^B and ^10^B isotopologues (81 : 19) of 4 calculated at the B3LYP-D3(BJ)/6-311+G(d,p) level of theory. (b) Difference IR spectrum after irradiation with 435 nm > *λ* > 620 nm for 30 min (following the annealing step). (c) Spectrum for ^11^B and ^10^B isotopologues (81 : 19) of 2 calculated at the B3LYP-D3(BJ)/6-311+G(d,p) level of theory.

Irradiation of matrix-isolated precursor 3 with *λ* = 254 nm forms 2 in the presence of CO. Annealing the matrix to 30 K allows the formation of distinct bands which decrease upon subsequent irradiation with 435 nm > *λ* > 620 nm, while the bands corresponding to 2 collectively form again (see Fig. 5). We assign the bands formed after annealing of the matrix to 30 K to the Lewis acid–base adduct 4 based on comparison with the vibrational spectrum computed at the B3LYP-D3(BJ)/6-311+G(d,p) level of theory (see Fig. 6). The formation of the (2 + 1) product 4_(2 + 1) can be excluded based on the computations (see Fig. S16†).

The most intense peak at 2112 cm^−1^ is due to the CO stretching vibration of the Lewis acid–base adduct 4 (Table S16†). It is observable at 2065 cm^−1^ and 2064 cm^−1^ for the C^18^O and ^13^CO isotopologues, respectively (Tables S17 and S18†). These isotopic shifts of 47 cm^−1^ (^12^C^18^O *vs.*^12^C^16^O) and 48 cm^−1^ (^13^C^16^O *vs.*^12^C^16^O) are in very good agreement with those computed for 4 (47 cm^−1^ and 50 cm^−1^, respectively, see Table S19†).

**Fig. 7:**
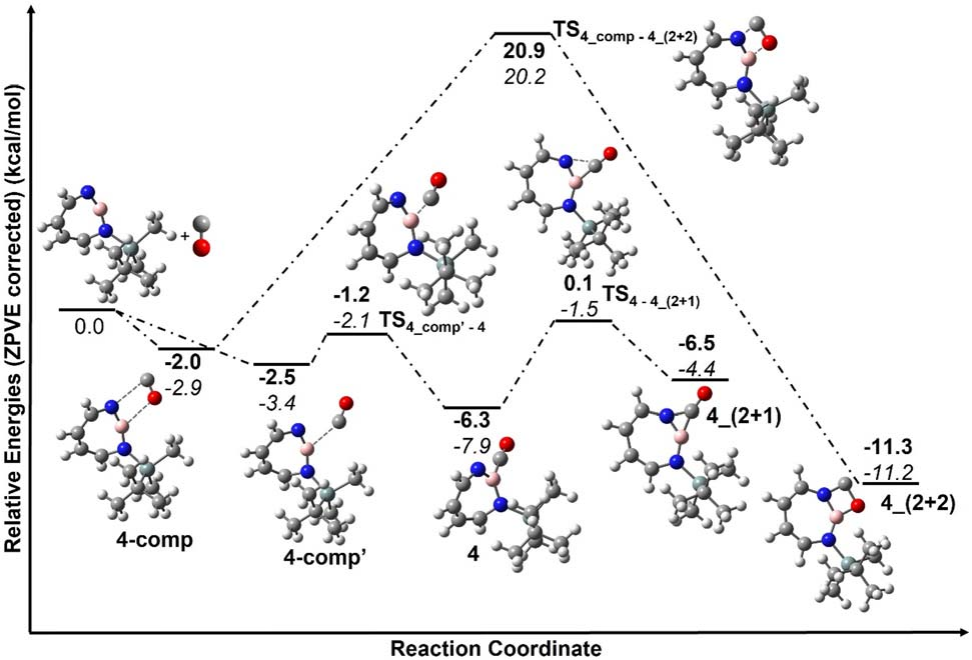
Reaction pathways for the reaction of 2 with CO at (bold: M06-2X/6-311+G(d,p); italics: DLPNO-CCSD(T)/cc-pVTZ//M06-2X/6-311+G(d,p)). Calculated ZPVE corrected energies in kcal mol^−1^ are shown.

The trapping of 2 with CO demonstrates that Lewis acid–base interaction is significantly preferred over (2 + 1) or (2 + 2) cycloaddition reactions, similar to the reaction of 1,2-azaborinine with CO.^66^ The formation of 4 has a barrier of 1.3 kcal mol^−1^ from the complex 4_comp′ at the DLPNO-CCSD(T)/cc-pVTZ//M06-2X/6-311+G(d,p) level of theory (Fig. 7). The formation of the (2 + 1) product 4_(2 + 1) from 4 is energetically uphill and cannot proceed thermally as its barrier of 6.4 kcal mol^−1^ (Fig. 7) is too high for this reaction to be observable at 30 K under matrix isolation conditions. The formation of the (2 + 2) cycloaddition product *via* complex 4_comp, the most stable product (−11.2 kcal mol^−1^ with respect to 2 + CO), is more favourable than 4 or 4_(2+1) by 3.3 kcal mol^−1^ and 6.8 kcal mol^−1^, respectively. But as it involves a barrier of 23.1 kcal mol^−1^ at the DLPNO-CCSD(T)/cc-pVTZ//M06-2X/6-311+G(d,p) level of theory, the reaction to 4_(2+2) cannot proceed under our experimental conditions. The trapping of 2 with CO demonstrates that Lewis acid–base interaction is significantly preferred over (2 + 1) or (2 + 2) cycloaddition reactions.

### Reaction of 2 with ethene

**Fig. 8:**
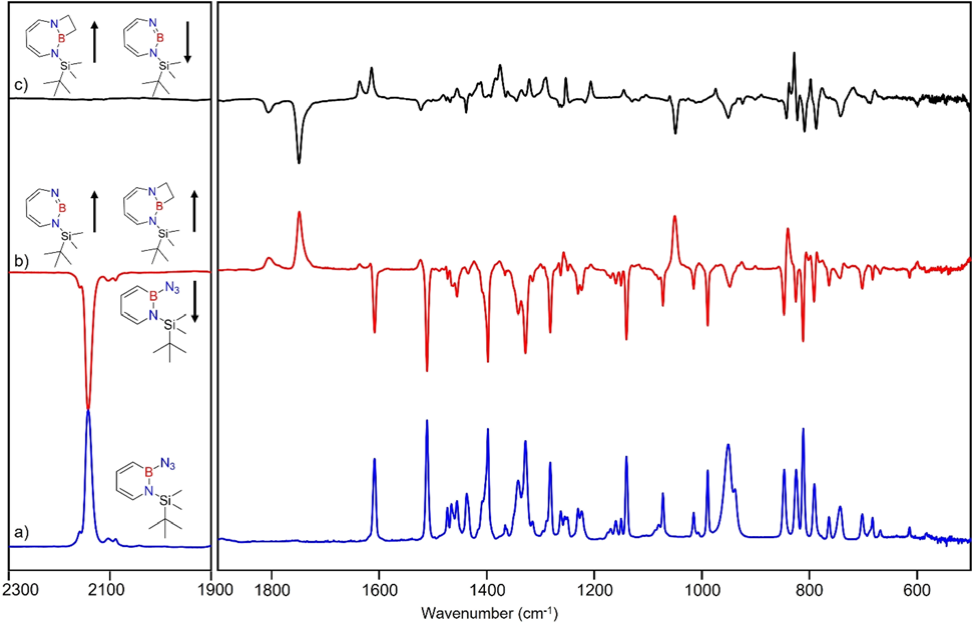
Infrared spectra obtained after (a) deposition of 3 in C_2_H_4_ (5%) doped argon matrix. (b) Difference spectrum after 60 min irradiation with *λ* = 254 nm at *T* = 4 K. (c) Difference spectrum after annealing for 30 min at 30 K (following the irradiation with *λ* = 254 nm).

**Fig. 9:**
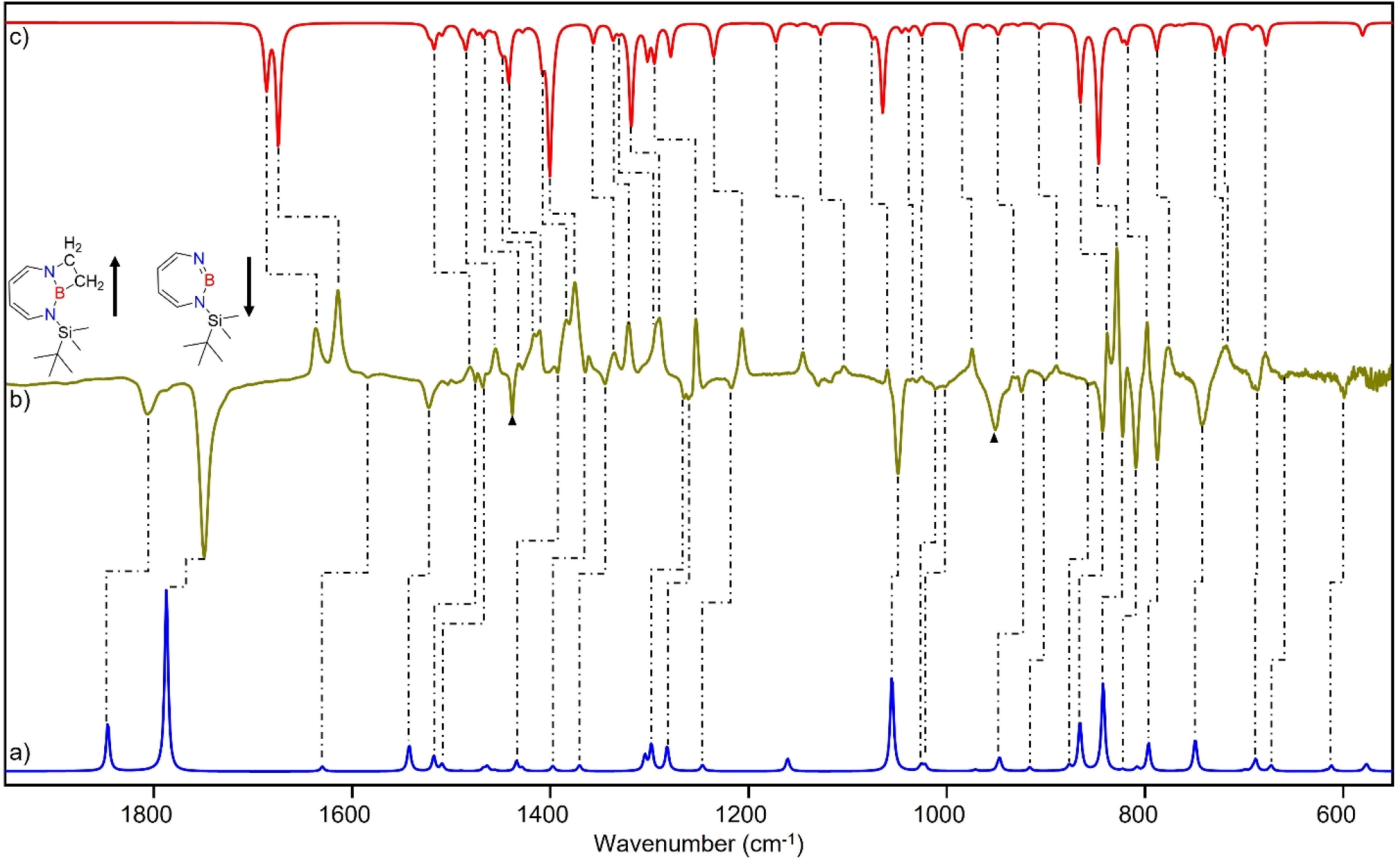
(a) Spectrum for ^11^B and ^10^B isotopologues (81 : 19) of 2 calculated at the B3LYP-D3(BJ)/6-311+G(d,p) level of theory. (b) Difference IR spectrum after annealing to 30 K for 60 min (following the irradiation step). (c) Spectrum for ^11^B and ^10^B isotopologues (81 : 19) of 5 calculated at the B3LYP-D3(BJ)/6-311+G(d,p) level of theory. (▲ corresponds to the peaks of C_2_H_4_)

After the successful generation of 2 and its trapping with CO, we used ethene as the simplest olefin for studying of the reactivity of 2 in (2 + 2) cycloaddition reactions. To examine the trapping of 2 with ethene, matrix-isolated precursor 3 was irradiated in the presence of 5% C_2_H_4_ in Ar with *λ* = 254 nm, which again resulted in 1*H*-1,3,2-diazaborepine 2. But apart from 2, new signals were also observed (Fig. 8) that we assigned with the help of the computed spectrum to 5, the (2 + 2) cycloaddition product between 2 and C_2_H_4_ (Scheme 2 and Fig. 9). Subsequently, the matrix was annealed to 30 K and it was observed that the set of signals corresponding to 2 were diminished (Fig. 8c), while the set of signals assigned to 5 increased in intensity (Fig. 8c). This shows that the formation of 5 from 2 and ethene can proceed thermally with a very low activation barrier.

The prominent and characteristic signals of 5, 1637 cm^−1^ and 1615 cm^−1^, are attributed to the C=C stretching vibration of the diazaborepine ring (Tables S20 and S21†). The same C=C stretching vibrations at 1637 cm^−1^ and 1615 cm^−1^ were also observed for the C_2_D_4_ isotope as the stretching mode does not involve any vibration of the ethene unit (Fig. S14†). The other most intense peaks were observed at 828 cm^−1^ and 1376 cm^−1^, which we assigned to a vibration including ring stretching and CH wagging of C_2_H_4_ and BN stretching and CH wagging, respectively. In case of C_2_D_4_, the most intense peak was observed at 1372 cm^−1^ attributed to BN stretching and CH wagging while a peak at 813 cm^−1^ corresponding to CH wagging of C_2_D_4_ was not so intense in case of reaction with C_2_D_4_. The experimentally observed isotopic shifts are in good agreement with the isotopic shift computed at the B3LYP-D3(BJ)/6-311+G(d,p) level of theory (see Table S22 in ESI†).

**Fig. 10:**
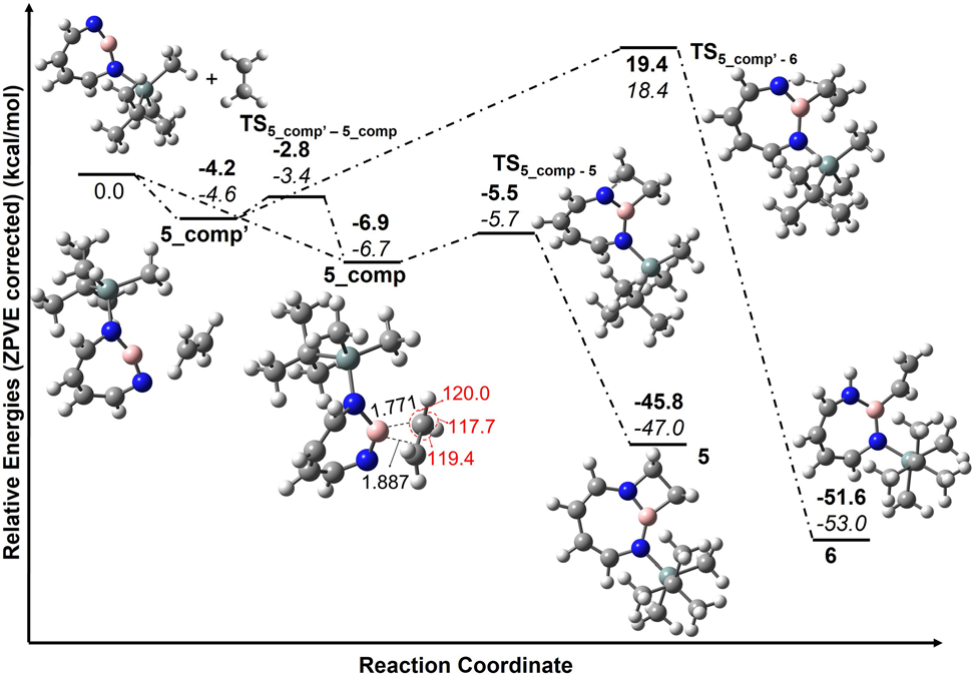
Pathways for the reaction of 2 with C_2_H_4_ at M06-2X/6-311+G(d,p) (bold) and DLPNO-CCSD(T)/cc-pVTZ//M06-2X/6-311+G(d,p) (italics). Calculated ZPVE corrected energies in kcal mol^−1^ are shown. Geometrical parameters of complex 5_comp computed at the M06-2X/6-311+G(d,p) level of theory. Important bond lengths [Å] and bond angles [°] are given.

According to computations at the DLPNO-CCSD(T)/cc-pVTZ//M06-2X/6-311+G(d,p) level of theory, 2 can interact with ethene to give two complexes, 5_comp′ and 5_comp, that are lower in energy than the separated reactants by 4.6 and 6.7 kcal mol^−1^, respectively (see Fig. 10). The interconversion between complexes 5_comp′ and 5_comp is associated with a barrier of 1.2 kcal mol^−1^. The formation of the (2 + 2) cycloaddition product 5 from complex 5_comp can proceed thermally as its barrier of 1.0 kcal mol^−1^ (Fig. 10) is small enough for this reaction to be observable at 30 K under matrix isolation conditions. Formation of the C–H insertion product 6*via* a concerted pathway from complex 5_comp′ involves a barrier of 23.0 kcal mol^−1^. This is significantly higher than the barrier of (2 + 2) addition product formation (Fig. 10) and explains the preferred formation of 5 in the experiment.

Complex 5_comp has carbon-boron distances (1.771 Å and 1.887 Å) that are shorter than the sum of van-der-Waals radii (3.62 Å) (see Fig. 10) indicative of a Lewis acid–base interaction.^90,91^ The H–C–H and H–C–C angles have changed so that the sum of bond angles around carbon is 357.1 and 359.6°, showing a slight deviation from planarity around carbon atoms in C_2_H_4_. The complex 5_comp is formed due to the interaction between the empty boron orbital with high p-character and the C=C π-bond. Further insight into the nature of bonding in 5_comp is provided by natural bond orbital analysis.^92–95^ The second-order perturbation theory analysis in the NBO basis gives an *E*(2) value of 314.8 kcal mol^−1^, implying strong stabilization due to π(C_2_H_4_) → n*(B)(empty orbital on boron) delocalization. The corresponding natural localized molecular orbitals (NLMO) have major contributions from the π(C=C) bond orbitals and “delocalization tails” of 20.6% from a slightly hybridized vacant orbital at boron (Tables S11 and S12†).

The comparison of the reaction mechanism for the reaction of ethene with 1,2-azaborinine 1 and 1-(*tert*-butyldimethylsilyl)-1,3,2-diazaborepine 2 is instructive. The former interacts with ethene to give a Lewis acid–base complex whose formation is thermodynamically highly exothermic (−23.7 kcal mol^−1^ at CCSD(T)/cc-pVTZ//M06-2X/6-311+G(d,p)).^67^ This complex is so low in energy that the formation of the (2 + 2) cycloaddition product has a barrier of 14.6 kcal mol^−1^ at CCSD(T)/cc-pVTZ//M06-2X/6-311+G(d,p).^67^ This is significantly higher than the barrier for the (2 + 2) cycloaddition product 5 (1.0 kcal mol^−1^ from 5_comp).^67^ It is highly interesting and unexpected that the much less Lewis acidic 2 is undergoing unprecedented (2 + 2) cycloaddition with ethene while the highly Lewis acidic 1 is expected to be trapped in the Lewis acid–base complex with ethene as this reaction should not be observable at 30 K under matrix isolation conditions. Preliminary experiments of 1 + C_2_H_4_ indicate that thermally initiated (2 + 2) cycloaddition is not occurring under similar matrix isolation conditions.

## Conclusions

We here for the first time reveal the generation, spectroscopic detection by matrix isolation, and reactivity studies of a seven-membered cyclic iminoborane of the 1*H*-1,3,2-diazaborepine type. The precursor 2-azido-1-(*tert*-butyldimethylsilyl)-1,2-dihydro-1,2-azaborinine (3) undergoes a photoinduced N_2_ extrusion and rearrangement to the 1-(*tert*-butyldimethylsilyl)-1*H*-1,3,2-diazaborepine 2 without detectable intermediates. Detailed combined experimental and computational investigations reveal that 2 shows high reactivity towards carbon monoxide and ethene even at the very low temperature. With CO, 2 reacts to form Lewis acid–base adduct 4 while the alternative (2 + 1) or (2 + 2) cycloaddition reactions were not observed due to high energy barriers. With C_2_H_4_, 2 undergoes (2 + 2) cycloaddition reaction to from product 5. The (2 + 2) cycloaddition with ethene discovered here shows that the 2 has the potential to provide novel modes of reactivity for the construction of BN-containing heterocycles.

## Data availability

The data underlying this study are available in the published article and its ESI.†

## Author contributions

D. G. investigation and writing of original draft, R. E. investigation and review & editing of the manuscript, H. F. B. conceptualization, supervision, providing resources, acquiring funding, and review & editing of manuscript.

## Conflicts of interest

The authors declare no conflict of interest.

## Supplementary Material

SC-015-D3SC04901A-s001
