# Supplementary material for: Strain induced reactivity of cyclic iminoboranes: the (2 + 2) cycloaddition of a 1H-1,3,2-diazaborepine with ethene
Source: Chem Sci. 2023 Dec 12;15(2):666–74. doi: 10.1039/d3sc04901a (PMC10763563; doi:10.1039/d3sc04901a)
Supplement: SC-015-D3SC04901A-s001 [file SC-015-D3SC04901A-s001.pdf]

Electronic Supplementary Material (ESI) for Chemical Science.
This journal is © The Royal Society of Chemistry 2023

Electronic Supporting Information

Strain induced reactivity of cyclic iminoboranes: The (2 + 2) cycloaddition of a 1-*H*-1,3,2-diazaborepine with ethene

Divanshu Gupta, Ralf Einholz, Holger F. Bettinger*

Institut für Organische Chemie, Universität Tübingen, Auf der Morgenstelle 18, 72076 Tübingen, Germany

*E-mail: holger.bettinger@uni-tuebingen.de

Contents

- Experimental and Computational Details S3
- Spectra (NMR, MS, UV-Vis and IR) and plots S7
- Relative ZPVE Energies for the Reaction of **2** with CO S18
- Relative Free Energies (ΔG) for the Reaction of **2** with CO S18
- Relative ZPVE Energies for the Reaction of **2** with C_2H_4 S19
- Relative Free Energies (ΔG) for the Reaction of **2** with C_2H_4 S19
- Isodesmic Reaction for Determining Lewis Acidity of **1** and **2** S20
- CASSCF Calculation to Understand the Ring Enlargement Process S23
- Intrinsic Reaction Coordinate (IRC) Path of the Reactions S26
- NBO Analysis and NBO plots of Complex **5_comp** S29
- Experimental and Computed Vibrational Frequencies of **3** in Ar Matrix S30
- Experimental and Computed Vibrational Frequencies of **2** in Ar Matrix S32
- Experimental and Computed Vibrational Frequencies of **4** in Ar Matrix S33
- Experimental and Computed Vibrational Frequencies of ^{13}CO - **4** in Ar Matrix S34
- Experimental and Computed Vibrational Frequencies of $C^{18}O$ - **4** in Ar Matrix S35
- Experimental and Computed Isotopic Shifts of **4** in Ar Matrix S36
- Experimental and Computed Vibrational Frequencies of **5** in Ar Matrix S36
- Experimental and Computed Vibrational Frequencies of C_2D_4 - **5** in Ar Matrix S37
- Experimental and Computed Isotopic Shifts of **5** in Ar Matrix S39
- Cartesian Coordinates of Stationary Points S40
- References S70

Experimental and Computational Details

General Procedure. All experiments were performed under anhydrous conditions using argon as protective gas. All commercially available compounds and dry solvents were purchased. ^{1}H and ^{13}C NMR spectra were referenced to tetramethylsilane, ^{11}B NMR spectra were referenced externally to BF_3•OEt_2. ^{1}H and ^{13}C spectra were calibrated to residual solvent signals. NMR spectra were recorded on a Bruker Avance III+ 400 spectrometer. MSD 5977 (Agilent) with DIP (SIM) was used for recording EI-MS. 1-(*Tert*-butyldimethylsilyl)-2-chloro-1,2-dihydro-1,2-azaborine and azidotrimethylsilane were purchased from abcr (Article ID AB541736 (now discontinued)) and AlfaAesar (Stock No. L00173), respectively.

Synthesis

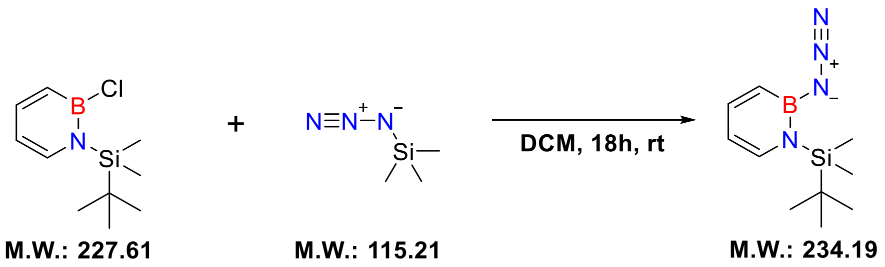

The reaction was conducted in a glove box under argon atmosphere. In a J-Young-NMR tube a solution of 45,4mg (0,2 mmol) 1-(*tert*-butyldimethylsilyl)-2-chloro-1,2-dihydro-1,2-azaborine in 0.5 mL deuterated dichloromethane was prepared. To this a solution of 23 mg (0.2 mmol) azidotrimethylsilane in 0.5 mL deuterated dichloromethane was added slowly by syringe. The progress of the reaction was monitored by NMR spectroscopy. After 18 h at room temperature all volatiles were removed under vacuum. 1-(*tert*-

butyldimethylsilyl)-2-azido-1,2-dihydro-1,2-azaborine remained and was used in all experiments without additional purification.

^{1}H-NMR: 400 MHz, CD_2Cl_2: 7.61 (1H, m), 7.15 (1H, m), 6.62 (1H, m), 6.19 (1H, m), 0.92 (9H, s), 0.47 (6H, s).

^{11}B{^{1}H}-NMR: 128 MHz, CD_2Cl_2: 31.9.

^{13}C{^{1}H}-NMR: 101 MHz, CD_2Cl_2: 146.3, 138.6, 121.5 (determined by 2D-NMR), 110.6, 26.6, 19.0, -2.8.

EI-MS: m/z (Int.%): 234.2 (25) M+, 177.1 (26) [M-C(CH_3)$_3$]+, 149.1 (100) [M-HC(CH_3)$_3$-N_2]+;

HRMS m/z: With our available equipment, high resolution mass spectrometry cannot be performed for air and moisture sensitive compounds.

Matrix Isolation Experimental Details

The precursor 1-(*tert*-butyldimethylsilyl)-2-azido-1,2-dihydro-1,2-azaborine **3** was sublimed from a glass flask at room temperature, and all gaseous materials leaving the flask were condensed onto a cold CsI window (for IR) or sapphire window (for UV-Vis) with a large excess of Argon 6.0 (Westfalen AG, 99.9999 %), or doped with 1-2 % of carbon monoxide 3.7 (Westfalen AG), $C^{18}O$ 2.0 (95 % ^{18}O) (Sigma Aldrich) or ^{13}CO 2.3 (99.1 % ^{13}C) (Westfalen AG) and 5 % of C_2H_4 (Sigma-Aldrich, 99.95 %) or C_2D_4 (Sigma-Aldrich, 99 %). The mixtures of these gases were dosed to 2.0 sccm by a mass flow controller (MKS mass flow PR400B). A Sumitomo SH-1 closed-cycle helium cryostat was

used to obtain the temperature as low as 4 - 7 K.[1] The infrared spectra in the range 400-4000 cm^{-1} were obtained using a Bruker Vertex 70 FTIR spectrometer with a standard resolution of 0.5 cm^{-1}, while electronic absorption spectra were measured using a Perkin Elmer Lambda 1050 spectrometer. For inducing photochemistry in the deposited matrix, a low-pressure mercury lamp (λ = 254 nm, PenRay) and high-pressure mercury lamps (USHIO, USH-508S) were used. The combination of dichroic mirrors of required wavelength (420 - 630 nm) range along with a Schott cutoff filter (GG-435) was employed to obtain 435 nm > λ > 630 nm.

Computational Details

All the structures were fully optimized using the M06-2X[2] global hybrid functional as well as B3LYP[3, 4] hybrid exchange–correlation energy functional along with Grimme's[5] London dispersion correction with Becke-Johnson damping B3LYP-D3(BJ).[3, 4] The 6-311+G(d,p) (split-valence triple-ζ with diffuse s and p functions for all atoms as well as d polarization functions on non-hydrogen atoms and p polarization functions for hydrogen)[6] basis set was adopted for all geometry optimizations. Harmonic vibrational frequencies were computed analytically which confirmed the nature of the stationary points as minima or first order saddle points (transition states) as well as to reproduce the simulated spectra of species **2-5** (including various isotopes). For identifying the combination bands and overtones, which were seen in addition to the fundamental bands in the experimental spectrum, anharmonic calculations were performed at B3LYP-D3(BJ)/6-311+G(d,p) level of theory using second-order vibrational perturbative approach.[7-9] Additionally, to reveal which minima are connected to the transition states, intrinsic reaction coordinate (IRC)[10,

[11] paths were calculated at the different level of theories for each reaction (see SI). To refine the energies, single point calculations based on all the structures were performed using domain based local pair natural orbital (DLPNO) coupled cluster theory with single, double, and a perturbative estimate of triple excitation (DLPNO-CCSD(T))[12-14] in conjunction with Dunning's[15, 16] triple-ζ (cc-pVTZ) basis set as implemented in ORCA 5.0.[17] The DLPNO-CCSD(T) computations used TightPNO cutoff for increased accuracy and the frozen core approximation. All density functional theory calculations were performed with Gaussian 16.[18] The excitation spectra were calculated using time-dependent DFT (TD-DFT) with Gaussian 16 at CAM-B3LYP/6-311+G(d,p) level of theory.[18, 19] Natural bond orbital analysis was carried with the NBO 6.0 program using the M06-2X/6-311+G(d,p) geometries.[20-23] Magnetic shielding values were computed by employing the GIAO method[24, 25] at B3LYP/6-311+G(d,p)//M06-2X/6-311+G(d,p) level of theory. To determine the Lewis acidity of the compounds, isodesmic reactions were computed at SMD(DCM)[26]/MN15[27]/def2-TZVP[28] level of theory.

Spectra

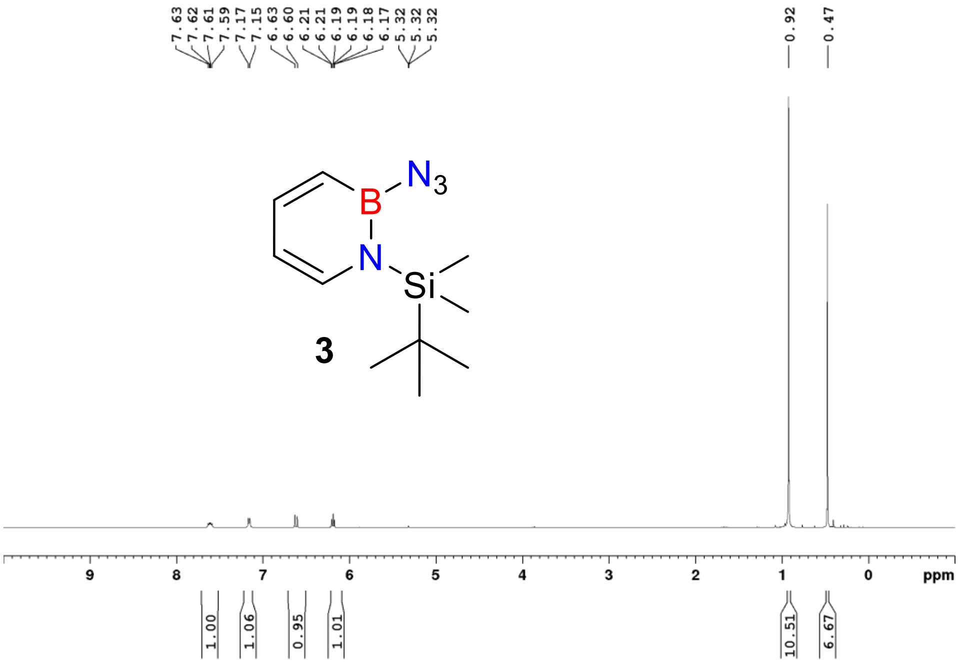

Figure S1. ^{1}H-NMR spectrum (400 MHz) of 1-(tert-butyldimethylsilyl)-2-azido-1,2-dihydro-1,2-azaborinine **3** in CD_2Cl_2.

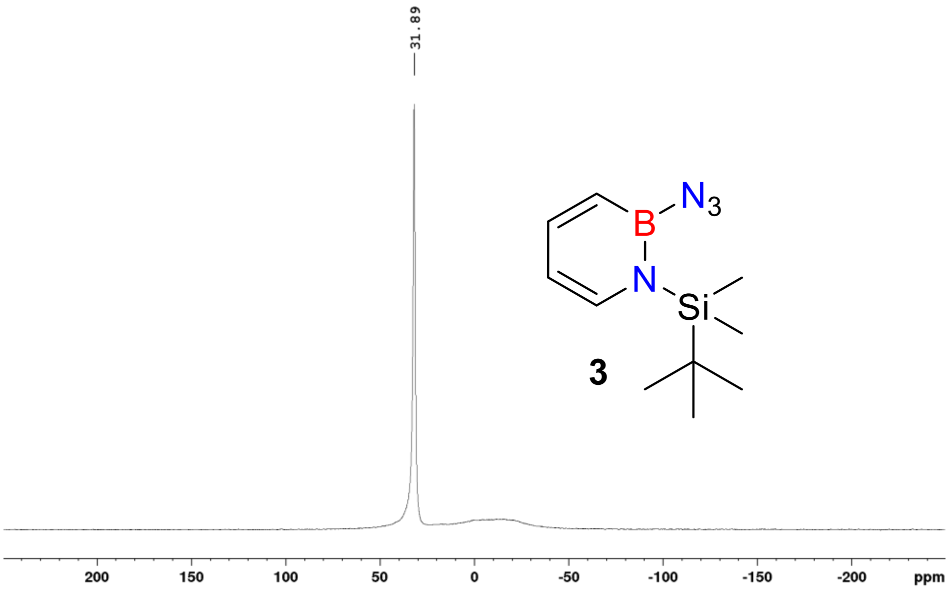

Figure S2. $^{11}B\{^{13}C\}$ -NMR spectrum (128 MHz) of 1-(tert-butyldimethylsilyl)-2-azido-1,2-dihydro-1,2-azaborinine **3** in CD_2Cl_2.

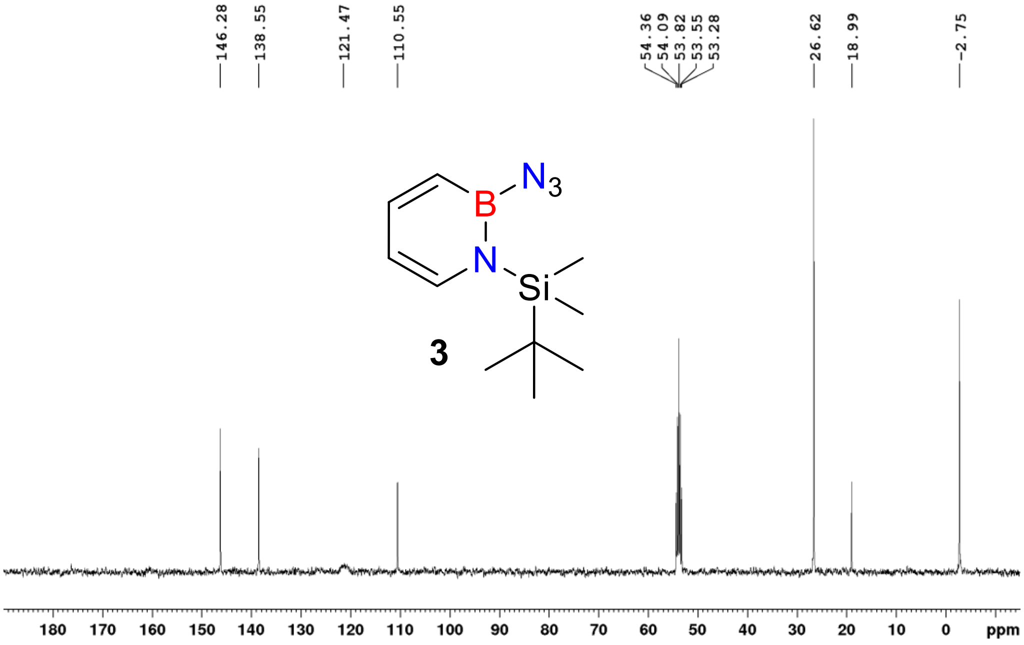

Figure S3. $^{13}C\{^{1}H\}$ -NMR spectrum (101 MHz) of 1-(tert-butyldimethylsilyl)-2-azido-1,2-dihydro-1,2-azaborinine **3** in CD_2Cl_2.

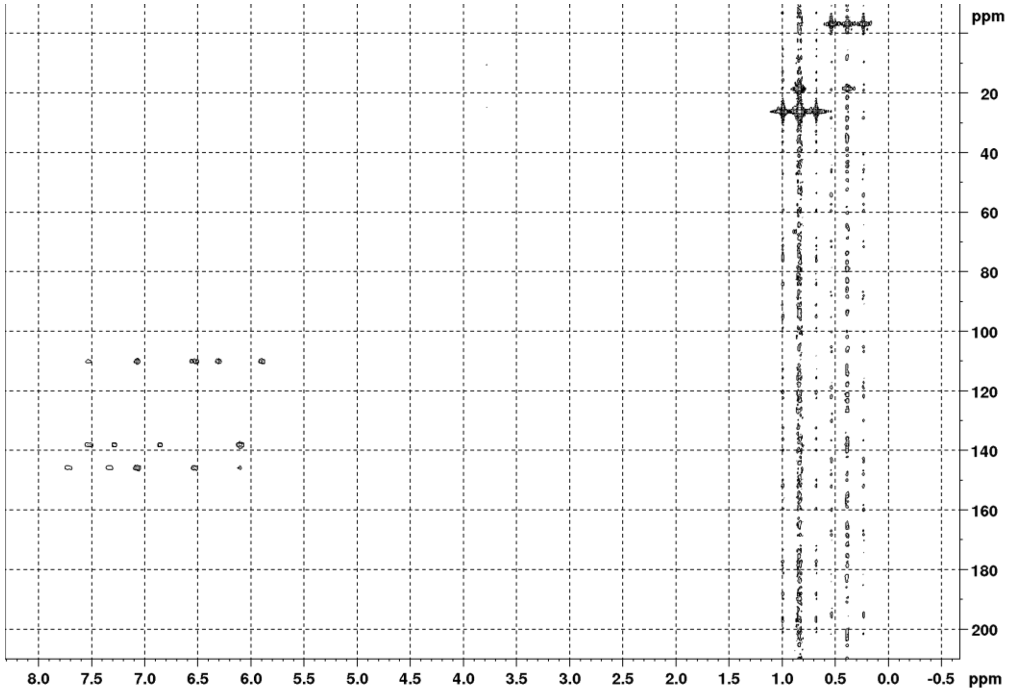

Figure S4. HMBC - NMR spectrum of 1-(tert-butyldimethylsilyl)-2-azido-1,2-dihydro-1,2-azaborinine **3** in CD_2Cl_2.

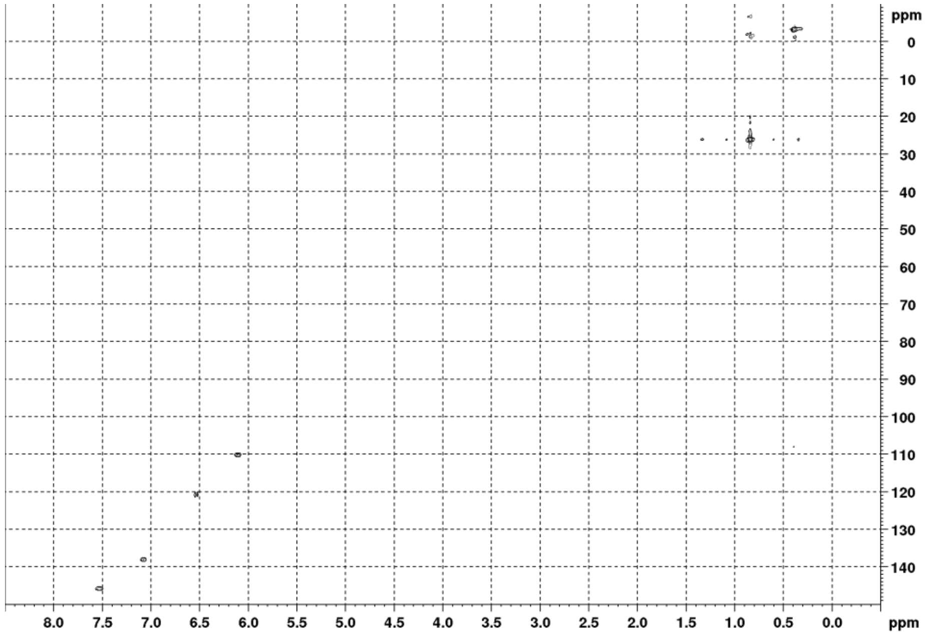

Figure S5. HSQC - NMR spectrum of 1-(tert-butyldimethylsilyl)-2-azido-1,2-dihydro-1,2-azaborinine **3** in CD_2Cl_2.

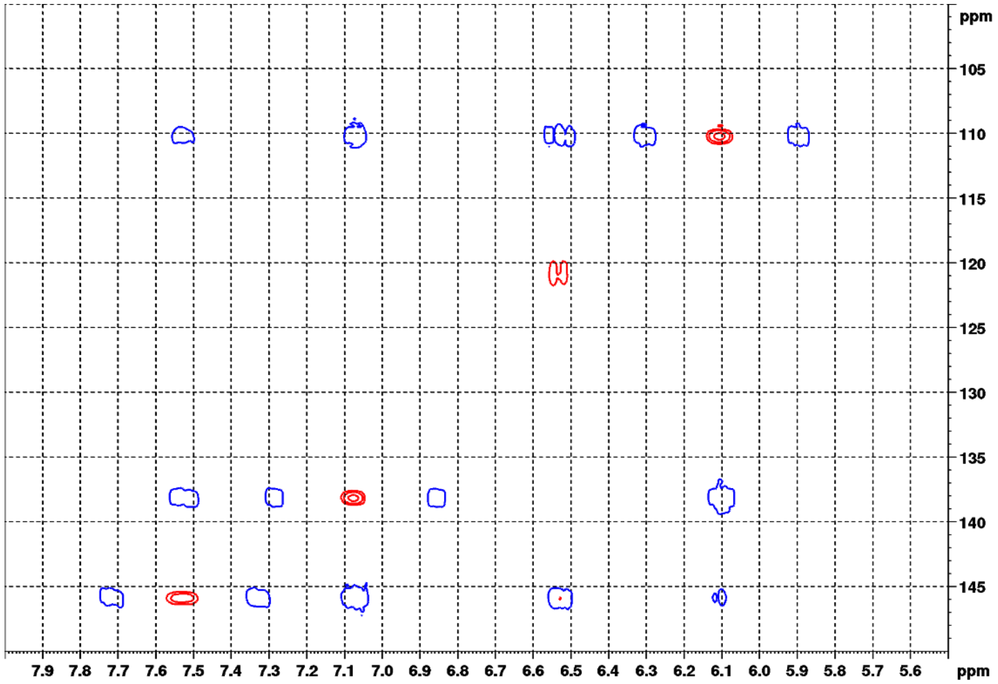

Figure S6. Overlapped HMBC-NMR and HSQC-NMR spectrum of 1-(tert-butyldimethylsilyl)-2-azido-1,2-dihydro-1,2-azaborinine **3** in CD_2Cl_2.

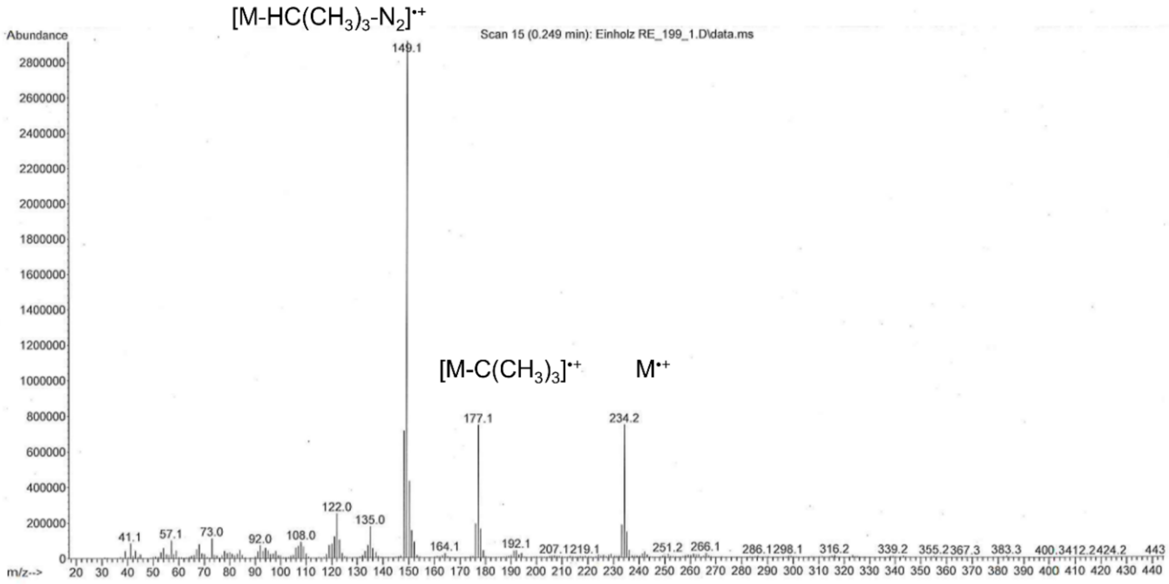

Figure S7. EI-MS spectrum of 1-(tert-butyldimethylsilyl)-2-azido-1,2-dihydro-1,2-azaborinine **3**.

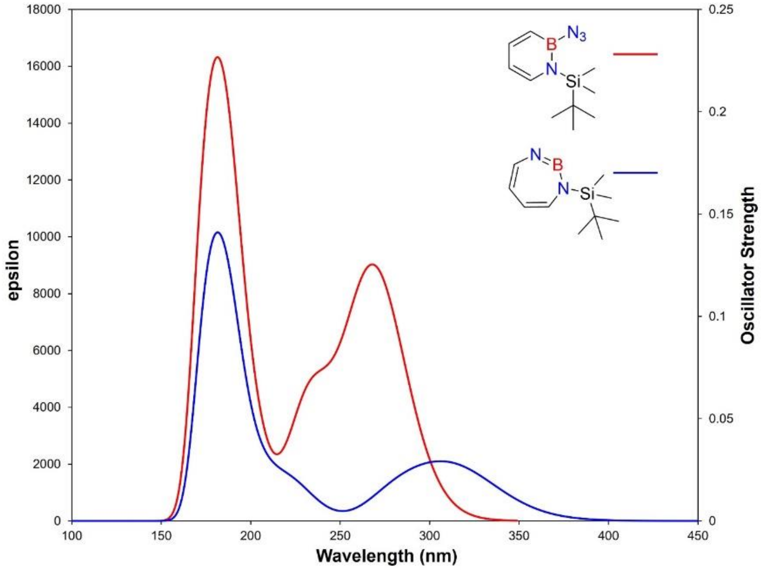

Figure S8. UV-Vis spectra of **3** (red) and **2** (blue) computed at TD-CAM-B3LYP/6-311+G(d,p) level of theory (half-width= 0.333 eV, first computed transition energy for **3** = 4.6 eV at λ_{max} = 269 nm, oscillator strength = 0.2157; first computed transition energy for **2** = 3.9 eV at λ_{max} = 317 nm, oscillator strength = 0.0403).

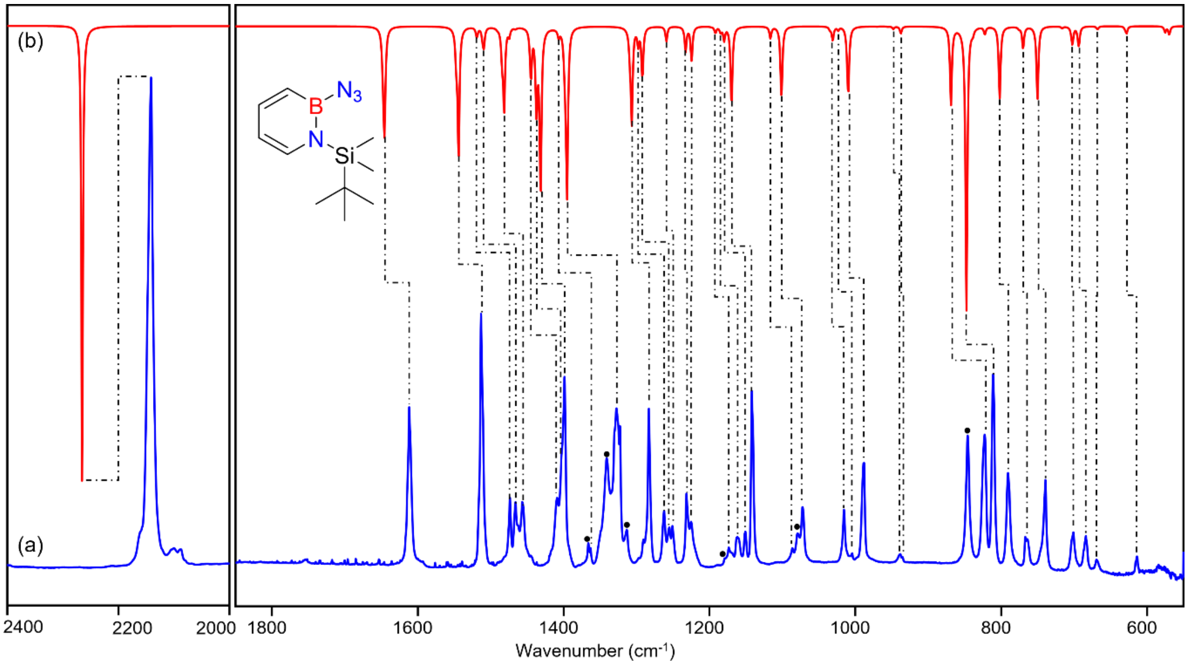

Figure S9. a) IR spectrum obtained after deposition of **3** in Ar matrix at 28 K. b) Spectrum for ^{11}B and ^{10}B isotopologues (81:19) of **3** calculated at the B3LYP-D3(BJ)/6–311+G(d,p) level of theory. (● corresponds to the overtones and combination bands (Table S9)).

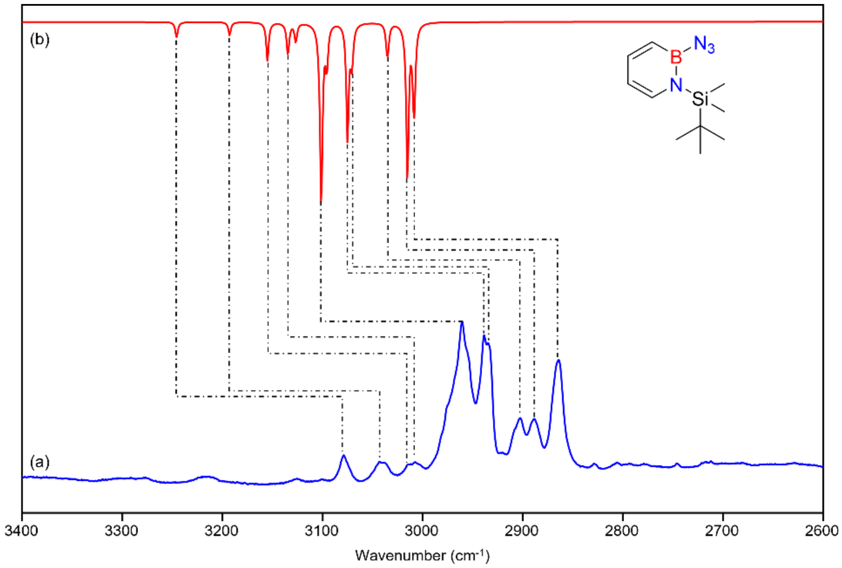

Figure S10. a) IR spectrum obtained after deposition of **3** in Ar matrix at 28 K. b) Spectrum for ^{11}B and ^{10}B isotopologues (81:19) of **3** calculated at the B3LYP-D3(BJ)/6–311+G(d,p) level of theory.

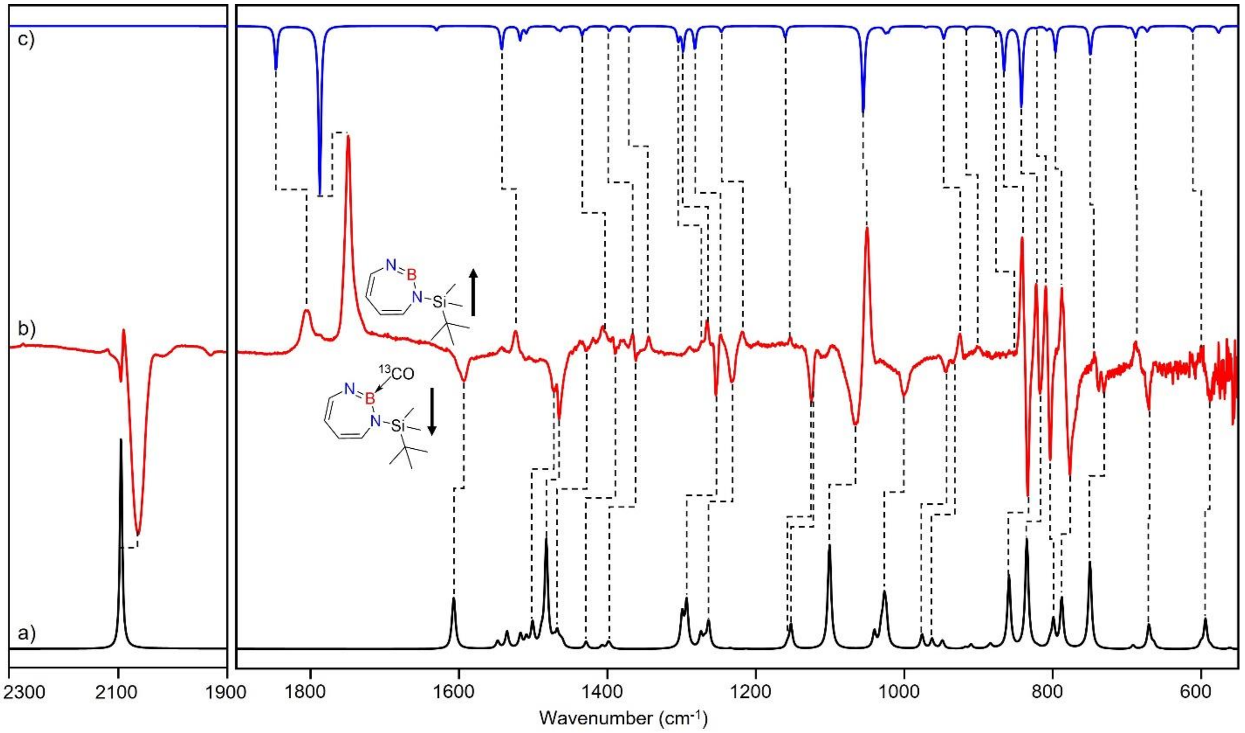

Figure S11. a) Spectrum for ^{11}B and ^{10}B (81:19) and ^{13}CO isotopologues of **4** calculated at the B3LYP-D3(BJ)/6–311+G(d,p) level of theory. b) Difference IR spectrum after irradiation with 435 nm > λ > 620 nm for 30 min (following the annealing step). c) Spectrum for ^{11}B and ^{10}B isotopologues (81:19) of **2** calculated at the B3LYP-D3(BJ)/6–311+G(d,p) level of theory.

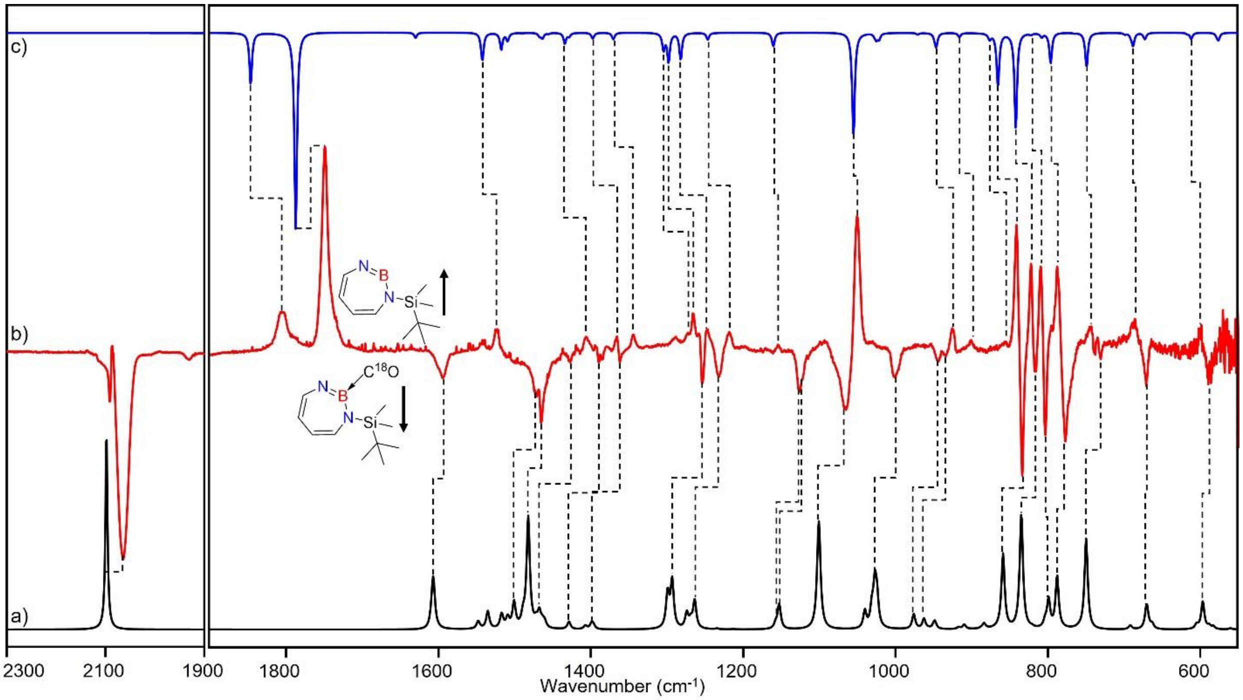

Figure S12. a) Spectrum for ^{11}B and ^{10}B (81:19) and $C^{18}O$ isotopologues of **4** calculated at the B3LYP-D3(BJ)/6–311+G(d,p) level of theory. b) Difference IR spectrum after irradiation with 435 nm > λ > 620 nm for 30 min (following the annealing step). c) Spectrum for ^{11}B and ^{10}B isotopologues (81:19) of **2** calculated at the B3LYP-D3(BJ)/6–311+G(d,p) level of theory.

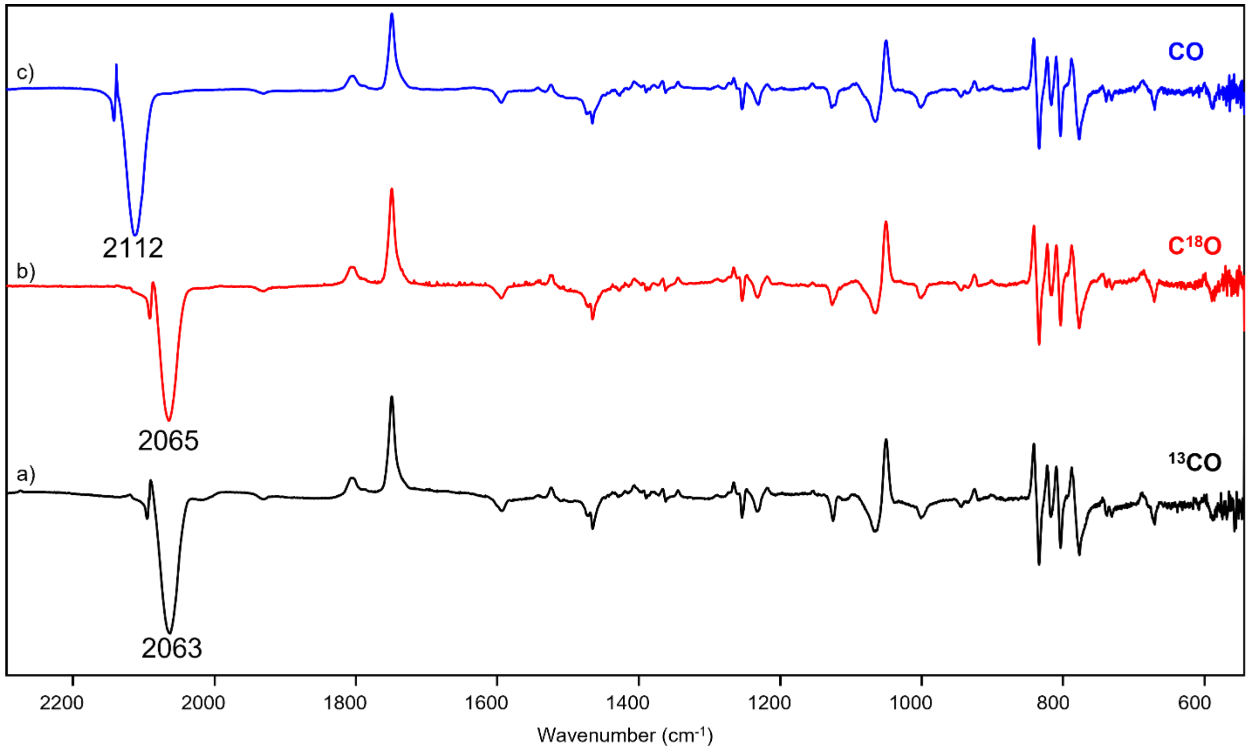

Figure S13. Difference IR spectrum after irradiation with 435 nm > λ > 620 nm for 30 min (following the annealing step) with a) $^{13}C^{16}O$ isotope, b) $^{12}C^{18}O$ isotope and c) $^{12}C^{16}O$ isotopes of **4** in Ar matrix.

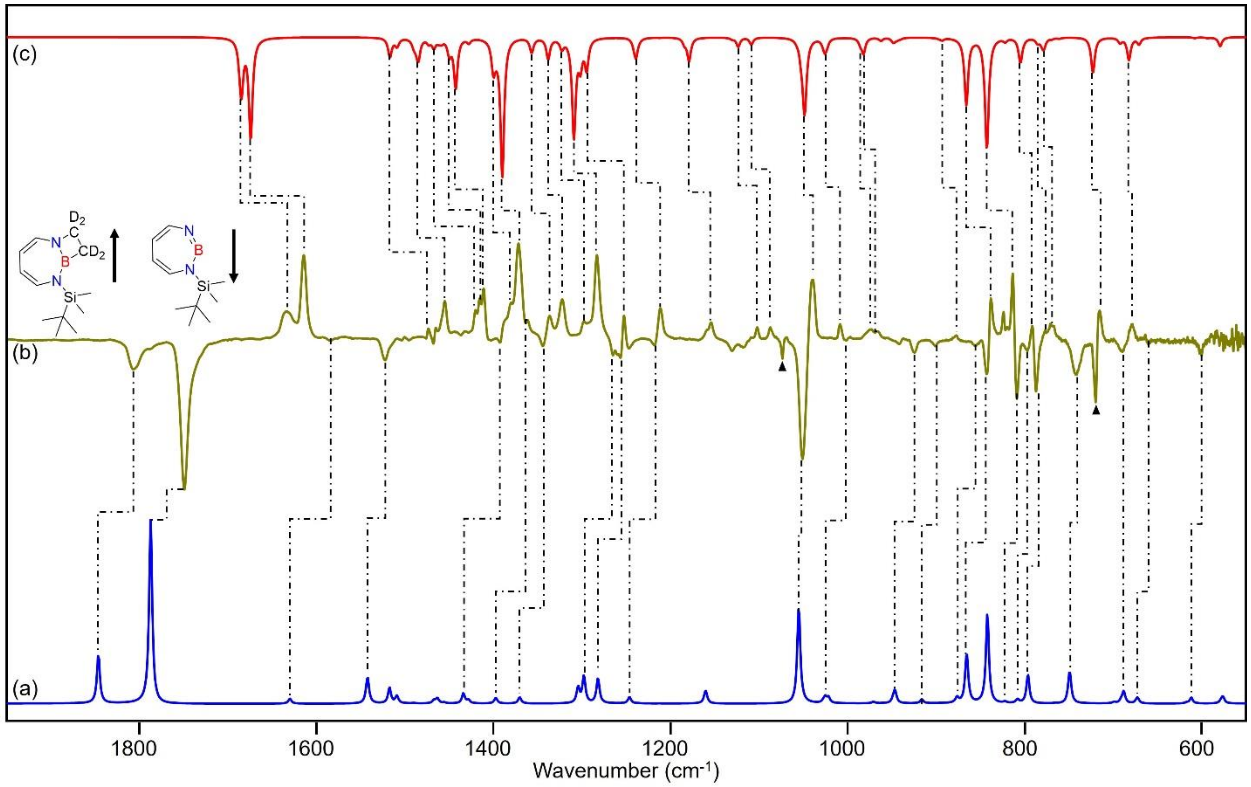

Figure S14. a) Spectrum for ^{11}B and ^{10}B isotopologues (81:19) of **2** calculated at the B3LYP-D3(BJ)/6–311+G(d,p) level of theory. b) Difference IR spectrum after annealing to 30 K for 60 min (following the irradiation step). c) Spectrum for ^{11}B and ^{10}B (81:19) and C_2D_4 isotopologues of **5** calculated at the B3LYP-D3(BJ)/6–311+G(d,p) level of theory. (▲ corresponds to the peaks of C_2D_4)

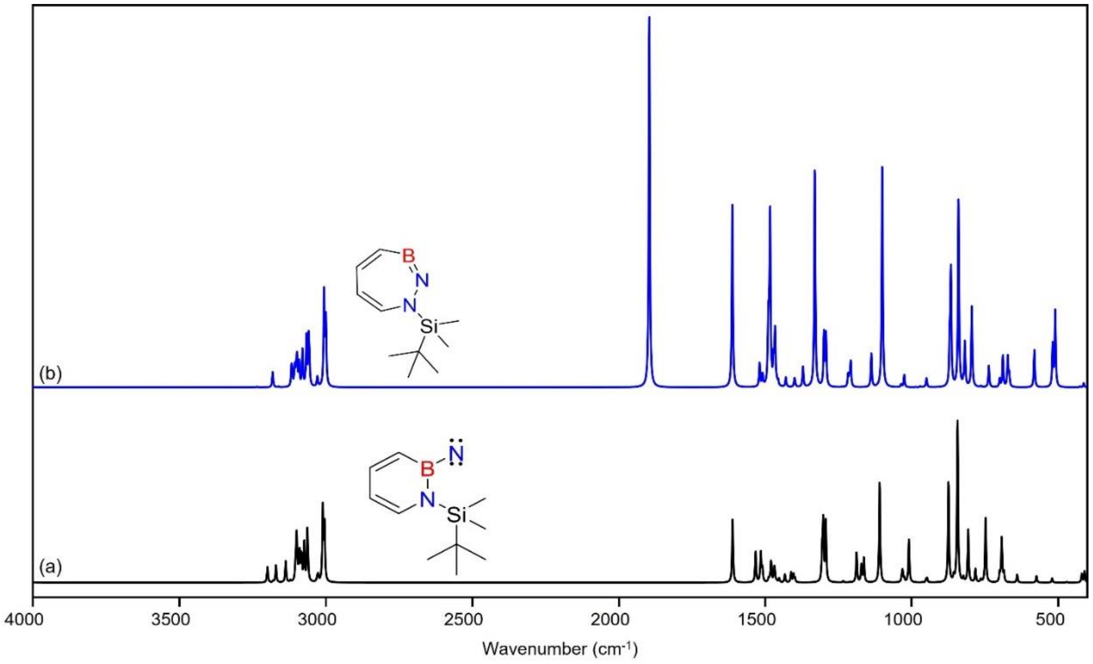

Figure S15. Spectrum for a) triplet boryl nitrene and b) 1*H*-1,2,3-diazaborepine calculated at the B3LYP-D3(BJ)/6–311+G(d,p) level of theory.

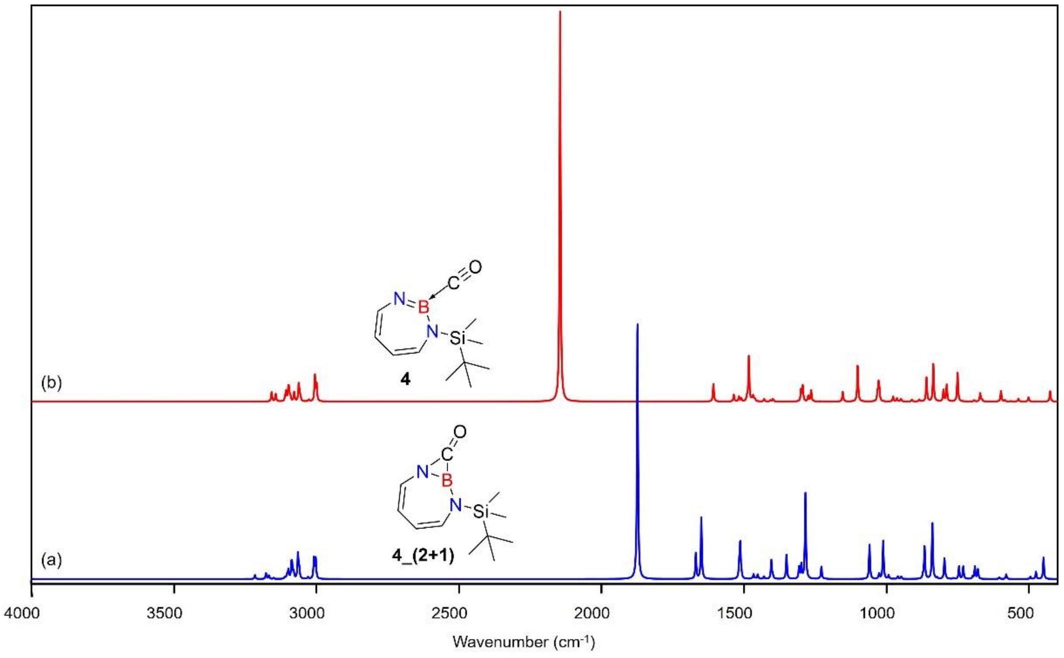

Figure S16. Spectrum for a) **4_(2 + 1)** and b) **4** calculated at the B3LYP-D3(BJ)/6–311+G(d,p) level of theory.

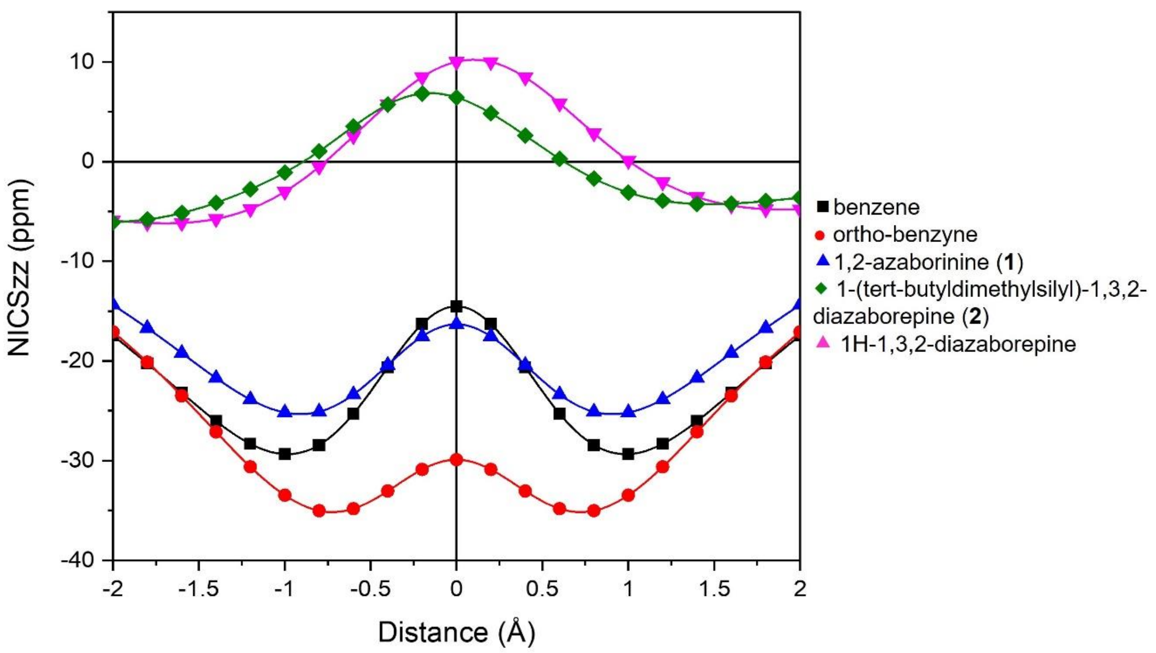

Figure S17. Plot of $NICS_{zz}$ value calculated along a line perpendicular to the molecular plane, starting at the ring center, at B3LYP/6-311+G(d,p)//M06-2X/6-311+G(d,p) level of theory.

Table S1. The relative ZPVE corrected energies in kcal/mol for the reaction of **2** with CO as computed at the different level of theories.

	**2 + CO**	**4_comp**	**4_comp'**	**$TS_{4comp'-4}$**	**4**	**$TS_{4-4\_(2+1)}$**	**4_(2+1)**	**$TS_{4\_comp-4\_(2+2)}$**	**4_(2+2)**
B3LYP-D3(BJ)/6-311+G(d,p) **[DLPNO-CCSD(T)/cc-pVTZ// B3LYP-D3(BJ)/6-311+G(d,p)]**	*0.0* **[0.0]**	*-2.0* **[-2.9]**	*-2.7* **[-3.2]**	*-2.4* **[-2.1]**	*-12.8* **[-7.0]**	*-4.6* **[-1.4]**	*-8.4* **[-4.6]**	*19.6* **[20.0]**	*-10.2* **[-11.1]**
M06-2X/6-311+G(d,p) **[DLPNO-CCSD(T)/cc-pVTZ// M06-2X/6-311+G(d,p)]**	*0.0* **[0.0]**	*-2.0* **[-2.9]**	*-2.5* **[-3.4]**	*-1.2* **[-2.1]**	*-6.3* **[-7.9]**	*0.1* **[-1.5]**	*-6.5* **[-4.4]**	*20.9* **[20.2]**	*-11.3* **[-11.2]**

Table S2. The relative free energies (ΔG at 298 K in kcal/mol) for the reaction of **2** with CO as computed at the different level of theories.

	**2+ CO**	**4_comp**	**4_comp'**	**$TS_{4comp'-4}$**	**4**	**$TS_{4-4\_(2+1)}$**	**4_(2+1)**	**$TS_{4\_comp-4\_(2+2)}$**	**4_(2+2)**
B3LYP-D3(BJ)/6-311+G(d,p) **[DLPNO-CCSD(T)/cc-pVTZ// B3LYP-D3(BJ)/6-311+G(d,p)]**	*0.0* **[0.0]**	*-2.0* **[-2.9]**	*-2.7* **[-3.2]**	*-2.4* **[-2.1]**	*-12.8* **[-7.0]**	*-4.6* **[-1.4]**	*-8.4* **[-4.6]**	*19.6* **[20.0]**	*-10.2* **[-11.1]**
M06-2X/6-311+G(d,p) **[DLPNO-CCSD(T)/cc-pVTZ// M06-2X/6-311+G(d,p)]**	*0.0* **[0.0]**	*-2.0* **[-2.9]**	*-2.5* **[-3.4]**	*-1.2* **[-2.1]**	*-6.3* **[-7.9]**	*0.1* **[-1.5]**	*-6.5* **[-4.4]**	*20.9* **[20.2]**	*-11.3* **[-11.2]**

Table S3. The relative ZPVE corrected energies in kcal/mol for the reaction of **2** with ethene (C_2H_4) as computed at the different level of theories.

	**2+ C_2H_4**	**5_comp'**	**$TS_{comp\_5'\text{-}comp\_5}$**	**5_comp**	**$TS_{comp\_5\text{-}5}$**	**5**	**$TS_{comp\_5'\text{-}6}$**	**6**
B3LYP-D3(BJ)/6-311+G(d,p) **[DLPNO-CCSD(T)/cc-pVTZ// B3LYP-D3(BJ)/6-311+G(d,p)]**	*0.0* **[0.0]**	*-3.8* **[-4.3]**	*-3.3* **[-3.3]**	*-7.8* **[-6.6]**	*-5.9* **[-5.7]**	*-41.6* **[-46.9]**	*15.8* **[18.6]**	*-49.6* **[53.1]**
M06-2X/6-311+G(d,p) **[DLPNO-CCSD(T)/cc-pVTZ// M06-2X/6-311+G(d,p)]**	*0.0* **[0.0]**	*-4.2* **[-4.6]**	*-2.8* **[-3.4]**	*-6.9* **[-6.7]**	*-5.5* **[-5.7]**	*-45.8* **[-47.0]**	*19.4* **[18.4]**	*-51.6* **[-57.1]**

Table S4. The relative free energies (ΔG at 298 K in kcal/mol) for the reaction of **2** with ethene (C_2H_4) as computed at the different level of theories.

	**2 + C_2H_4**	**5_comp'**	**$TS_{comp\_5'\text{-}comp\_5}$**	**5_comp**	**$TS_{comp\_5\text{-}5}$**	**5**	**$TS_{comp\_5'\text{-}6}$**	**6**
B3LYP-D3(BJ)/6-311+G(d,p) **[DLPNO-CCSD(T)/cc-pVTZ// B3LYP-D3(BJ)/6-311+G(d,p)]**	*0.0* **[0.0]**	*4.0* **[3.6]**	*6.6* **[6.7]**	*3.5* **[4.7]**	*5.5* **[5.7]**	*-30.1* **[-35.4]**	*26.7* **[29.5]**	*-38.0* **[-41.5]**
M06-2X/6-311+G(d,p) **[DLPNO-CCSD(T)/cc-pVTZ// M06-2X/6-311+G(d,p)]**	*0.0* **[0.0]**	*7.2* **[6.6]**	*4.7* **[4.9]**	*4.7* **[4.9]**	*6.6* **[6.4]**	*-34.9* **[-36.1]**	*30.4* **[29.5]**	*-39.9* **[-41.2]**

Isodesmic Reactions as Tool to Calculate Equilibrium Constants for determining Lewis Acidity

We determined the Lewis acidity of **1** and **2** using the computational protocol used by Ofial et al. by determining the Lewis acidity from equilibrium constants of Lewis acid-base adduct formation.[29] We used the following equations (Figure S17) for the computation of Gibbs energies of association of boranes and Lewis bases at the SMD(DCM)/MN15/def2-TZVP level of theory following Ofial et al.[29] ΔG^{exptl} values are taken from the paper by Ofial et al.[29]

$$\text{(I)} \quad \text{X} + \text{BAr}_3\text{-LB} \xrightarrow{\Delta G(\text{I}) \text{ by DFT}} \text{X-LB} + \text{BAr}_3$$

$$\text{(II)} \quad \text{BAr}_3 + \text{LB} \xrightarrow{\Delta G(\text{II})^{exptl}} \text{BAr}_3\text{-LB}$$

$$\text{(III)} \quad \text{X} + \text{LB} \xrightarrow{\Delta G_{iso}} \text{X-LB} \qquad \Delta G_{iso} = \Delta G(\text{I}) + \Delta G(\text{II})^{exptl}$$

Figure S17. Combining the isodesmic reaction [Eq. (I)] with an experimental reference reaction [Eq.(II)] allows one to determine the Lewis acidities of X from ΔG_{iso} [Eq.(III)].

To assess the Lewis acidity LA_B of 1,2-azaborinine **1** and 1,3,2-diazaborepine **2**, triaryl boranes (BAr_3) (Ar= C_6H_5 (**7a**), 4-Cl-C_6H_4 (**7b**), 3,4,5-F_3-C_6H_2 (**7c**)) were chosen as reference Lewis acids. We used three structurally diverse Lewis bases (pyridine (**8a**), acetonitrile (**8b**), and benzaldehyde (**8c**)) as reference to estimate the LA_B values. Based on the experimental ΔG for the formation of Lewis adducts of **7a•8a**, **7b•8b**, and **7c•8c**, we estimate LA_B values of azaborinine **1** or 1,3,2-diazaborepine **2**. As outlined in Figure S17, the Gibbs reaction energies, ΔG(I), for isodesmic Lewis base transfer reactions between the Lewis adduct Ar_3B–LB and X (**1** or **2**) were computed by using the

SMD(DCM)/MN15/def2-TZVP method. To determine LA_B of **1** and **2**, experimentally determined Gibbs energy, ΔG^{exptl} (Figure S17), for the formation of a Lewis adduct of Ar_3B and the investigated Lewis base was added to ΔG(I) to give ΔG_{iso} (Figure S17). The Gibbs energy, ΔG_{iso}, in Equation III (Figure S17) now describes the thermodynamics of Lewis adduct formation between X (X = **1** or **2**) and a Lewis base, which can be used to estimate the Lewis acidity, LA_B, of **1** or **2** if LB_B of the Lewis base is known according to the following equation:

$$\log K_B = LA_B + LB_B$$

Table S5. Energies of the reaction of equation (I), (II), and (III) with pyridine (**8a**) and BAr_3 (**7a**) as reference Lewis base and acid, respectively, at the SMD(DCM)/MN15/def2-TZVP level of theory and calculation of LA_B via an isodesmic reaction.

Species (X)	ΔG(I) (kJ/mol)	$\Delta G(II)_{exptl}$[a] (kJ/mol)	ΔG_{iso} (kJ/mol)	K_B (M^{-1})[b]	LA_B[b]
**1**	-139.8	-34.5	-174.3	$1.2 * 10^{31}$	25.0
**2**	-71.6	-34.5	-106.1	$8.3 * 10^{18}$	12.8

[a] Experimental K_B for the reaction of 7a with 8a (see SI of ref. [29]) converted to ΔG with $\Delta G = -RT \ln K_B$. [b] At 20 °C.

Table S6. Energies of the reaction of equation (I), (II), and (III) with acetonitrile (**8b**) and BAr_3 (**7b**) as reference Lewis base and acid, respectively, at the SMD(DCM)/MN15/def2-TZVP level of theory and calculation of LA_B via an isodesmic reaction.

Species (X)	ΔG(I) (kJ/mol)	$\Delta G(II)_{exptl}$[a] (kJ/mol)	ΔG_{iso} (kJ/mol)	K_B (M^{-1})[b]	LA_B[b]
**1**	-109.9	-3.9	-113.8	$1.9 * 10^{20}$	20.8
**2**	-36.2	-3.9	-40.1	$1.4 * 10^{7}$	7.6

[a] Experimental K_B for the reaction of **7b** with **8b** (see SI of ref. [29]) converted to ΔG with $\Delta G = –RT \ln K_B$. [b] At 20 °C.

Table S7. Energies of the reaction of equation (I), (II) and (III) with benzaldehyde (**8c**) and BAr_3 (**7c**) as reference Lewis base and acid, respectively, at the SMD(DCM)/MN15/def2-TZVP level of theory and calculation of LA_B via an isodesmic reaction.

Species (X)	ΔG(I) (kJ/mol)	$\Delta G(II)_{exptl}$[a] (kJ/mol)	ΔG_{iso} (kJ/mol)	K_B (M^{-1})[b]	LA_B[b]
**1**	-81.7	-13.2	-94.9	$8.4 * 10^{16}$	18.7
**2**	-15.8	-13.2	-29.0	$1.5 * 10^{5}$	6.9

[a] Experimental K_B for the reaction of **7c** with **8c** (see SI of ref. [29]) converted to ΔG with $\Delta G = –RT \ln K_B$. [b] At 20 °C.

Table S8. LA_B from the three reference reactions and averaged LA_B.

Lewis Acid	LA_B from 8a	LA_B from 8b	LA_B from 8c	LA_B Average
**1**	25.0	20.8	18.7	21.5 ± 2.6
**2**	12.8	7.6	6.9	9.1 ± 2.6

CASSCF Calculation to Understand the Ring Enlargement Process.

Complete active space self-consistent field (CASSCF) theory in combination with def2-SV(P)[28] basis set was used to compute the energies of nitrene electronic states. We considered a model system without the TBS group to interpret compound **2**. The active space consisted of 8 orbitals and 8 electrons (Figure S18). We also computed single point energies with fully internally contracted N-electron valence state perturbation theory (FIC-NEVPT2)[30] to include the effects of dynamic correlation. This results in singlet A'' state as the lowest singlet state which is energetically very close to the A' singlet state (Table S9).

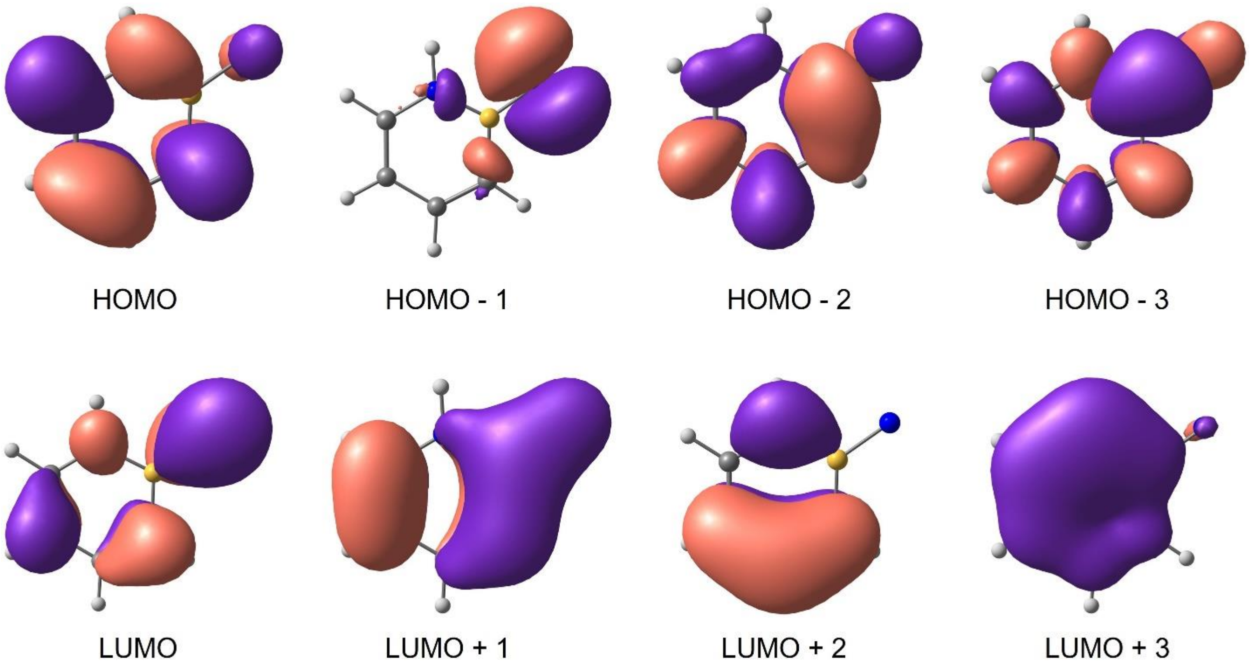

Figure S18. Active space orbitals used for the calculation of transition energies at CASSCF(8,8)/def2-SV(P) level of theory.

Table S9. Transition energies obtained with FIC-NEVPT2/def2-SV(P)//CASSCF(8,8)/def2-SV(P) (bold) and CASSCF(8,8)/def2-SV(P) (italics) level of theory.

State	Root	Multiplicity	IRREP	ΔE (kcal/mol)
0	0	3	A"	**0.0** / *0.0*
1	0	1	A"	**33.60** / *37.38*
2	0	1	A'	**34.89** / *37.54*
3	0	3	A'	**120.35** / *119.52*

We also scanned the potential energy surface by fixing the C-B-N angle in the $^1A'$ state to determine the barrier for the ring enlargement reaction (Figure 20). For this we considered an active space consisting of 10 orbitals and 10 electrons (Figure 19).

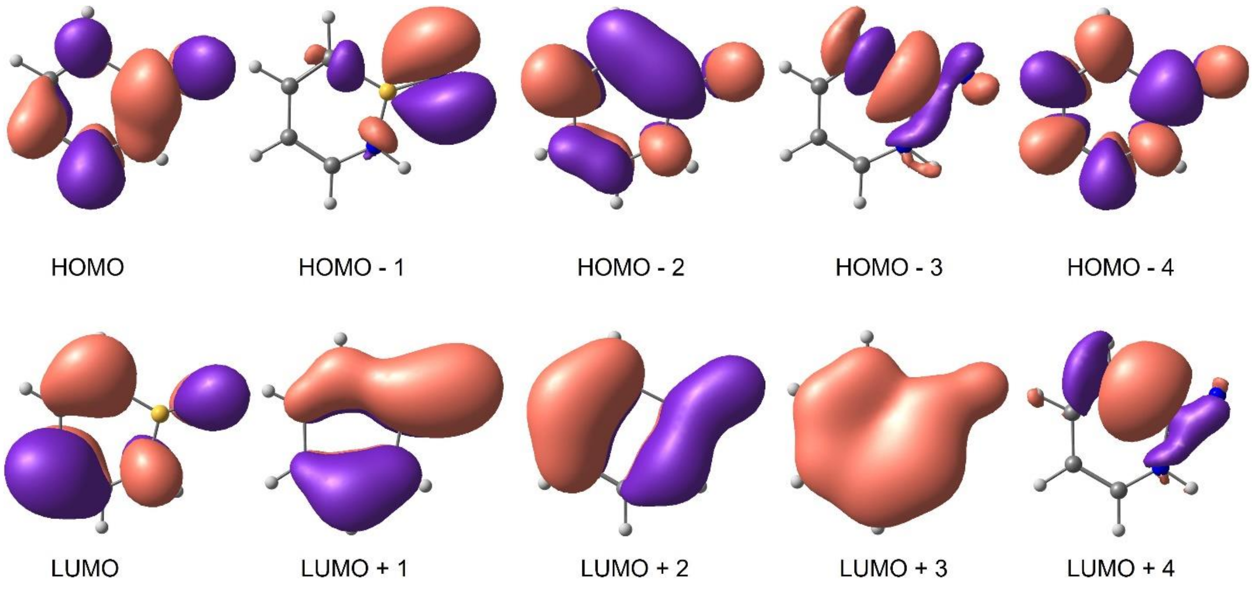

Figure S19. Active space orbitals used for the scan calculation of angle CBN at CASSCF(10,10)/def2-SV(P) level of theory.

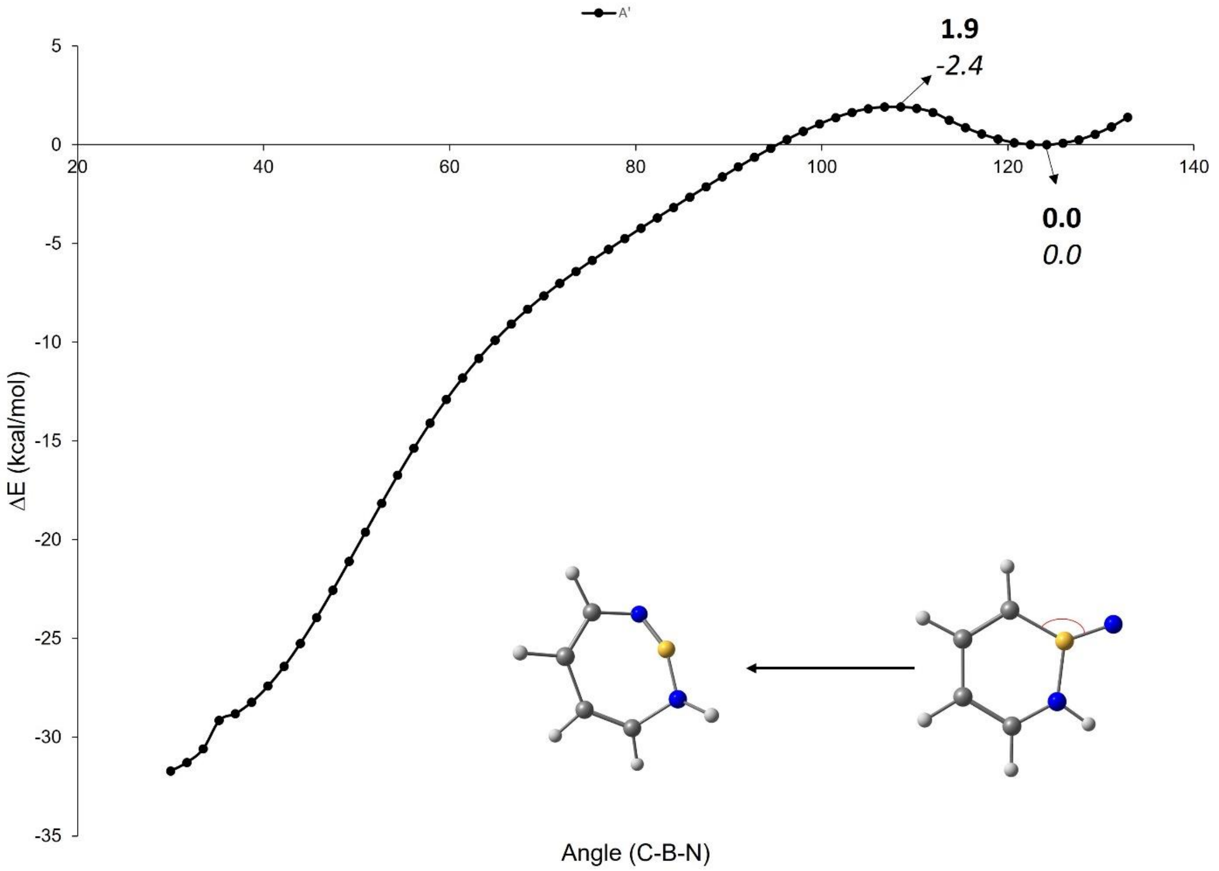

Figure S20. Scan calculation of angle CBN at CASSCF(10,10)/def2-SV(P) (bold) and single point at maximum and minimum at FIC-NEVPT2/def2-SV(P)//CASSCF(10,10)/def2-SV(P) (italics) level of theory.

The barrier is very small (1.9 kcal/mol) at the CASSCF level of theory, and it disappears entirely at NEVPT2//CASSCF. This implies that the singlet nitrene is not an observable species and N_2 extrusion and ring enlargement are concerted.

Table S10. Intrinsic reaction coordinate (IRC) path for the reactions of **2** with CO and C_2H_4 respectively at M06-2X/6-311+G(d,p) level of theory.

IRC for **TS$_{4_comp' - 4}$**
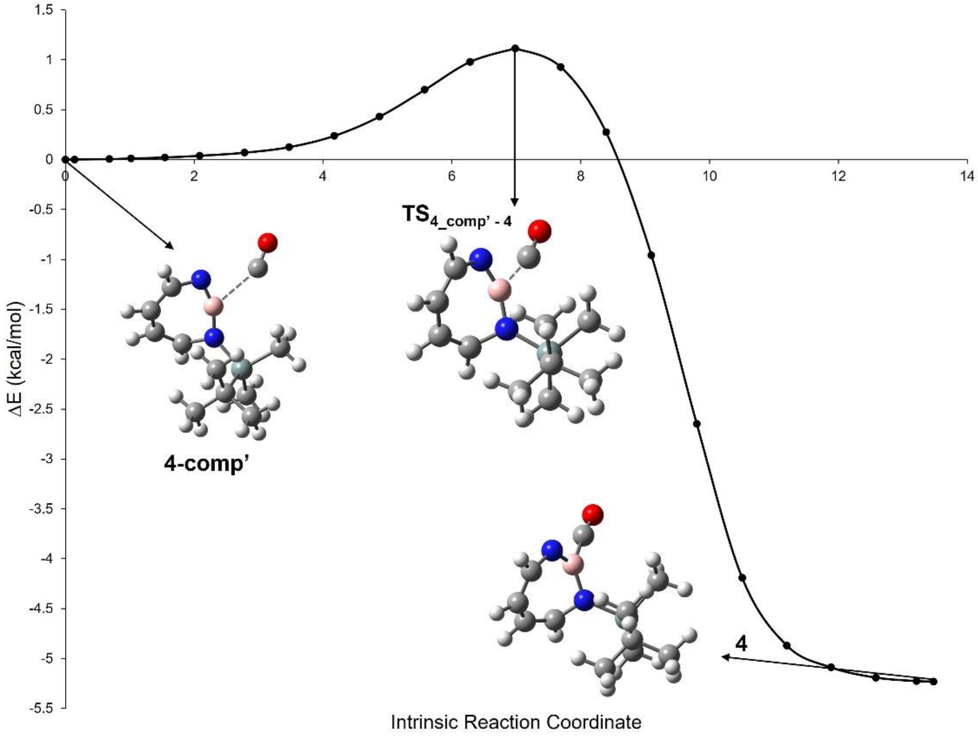
IRC for **TS$_{4 – 4_(2+1)}$**
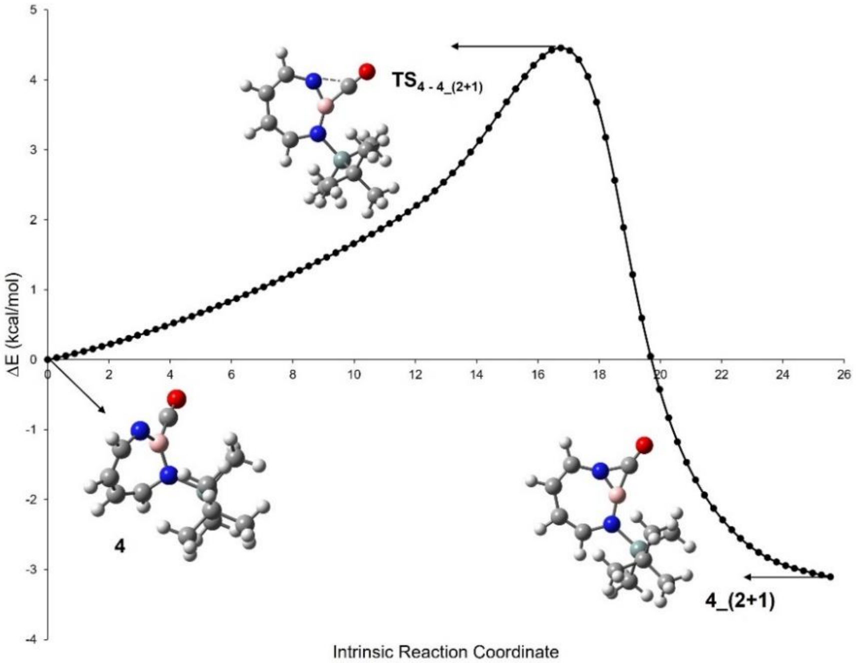

IRC for **TS$_{4_comp – 4_(2+2)}$**

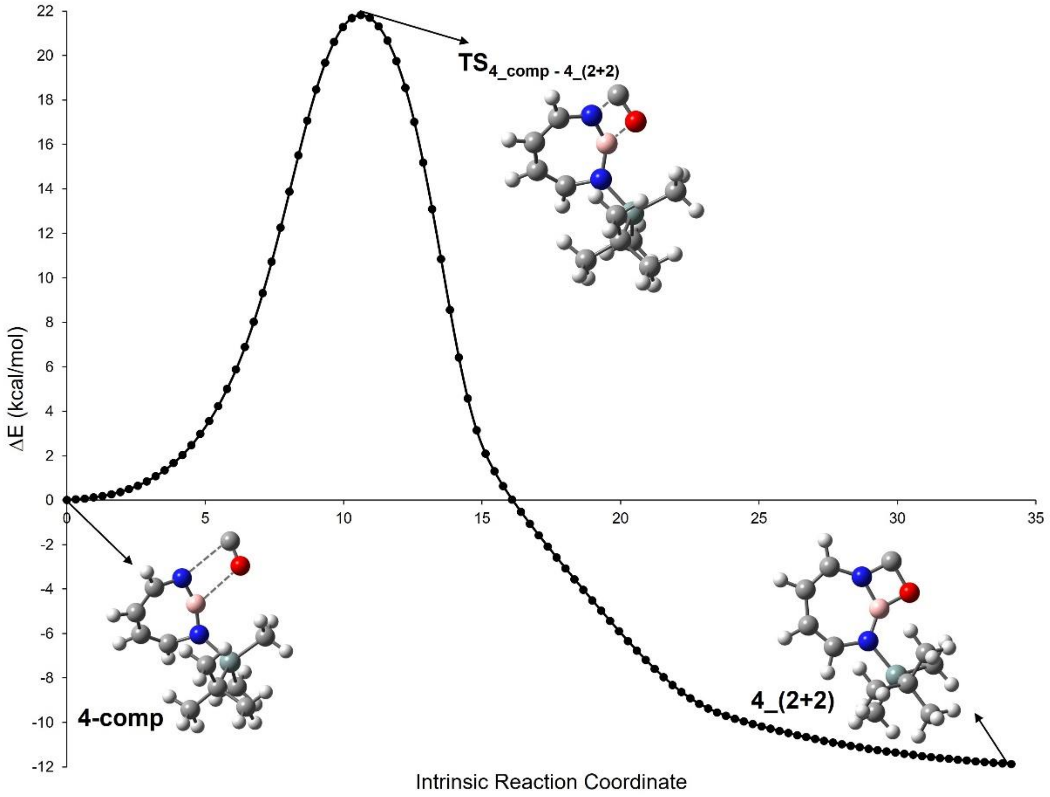

IRC for **TS$_{comp_5' – comp_5}$**

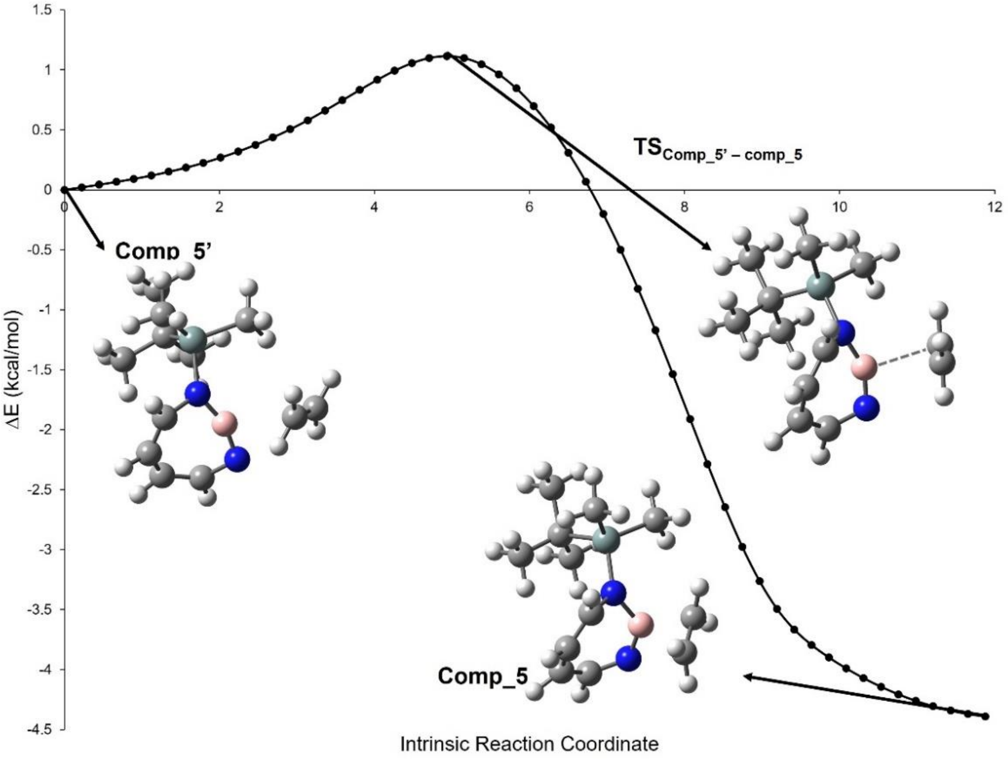

IRC for **TS$_{comp_5-5}$**

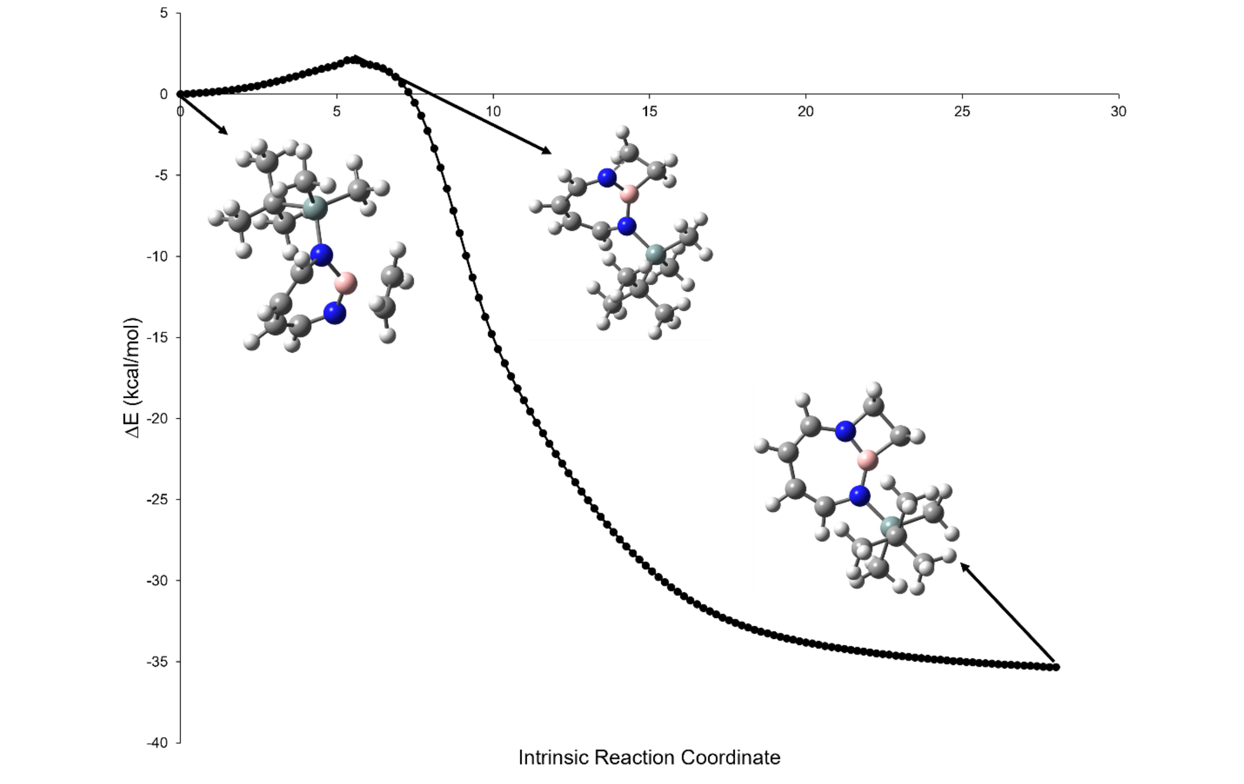

IRC for **TS$_{comp_5'-6}$**

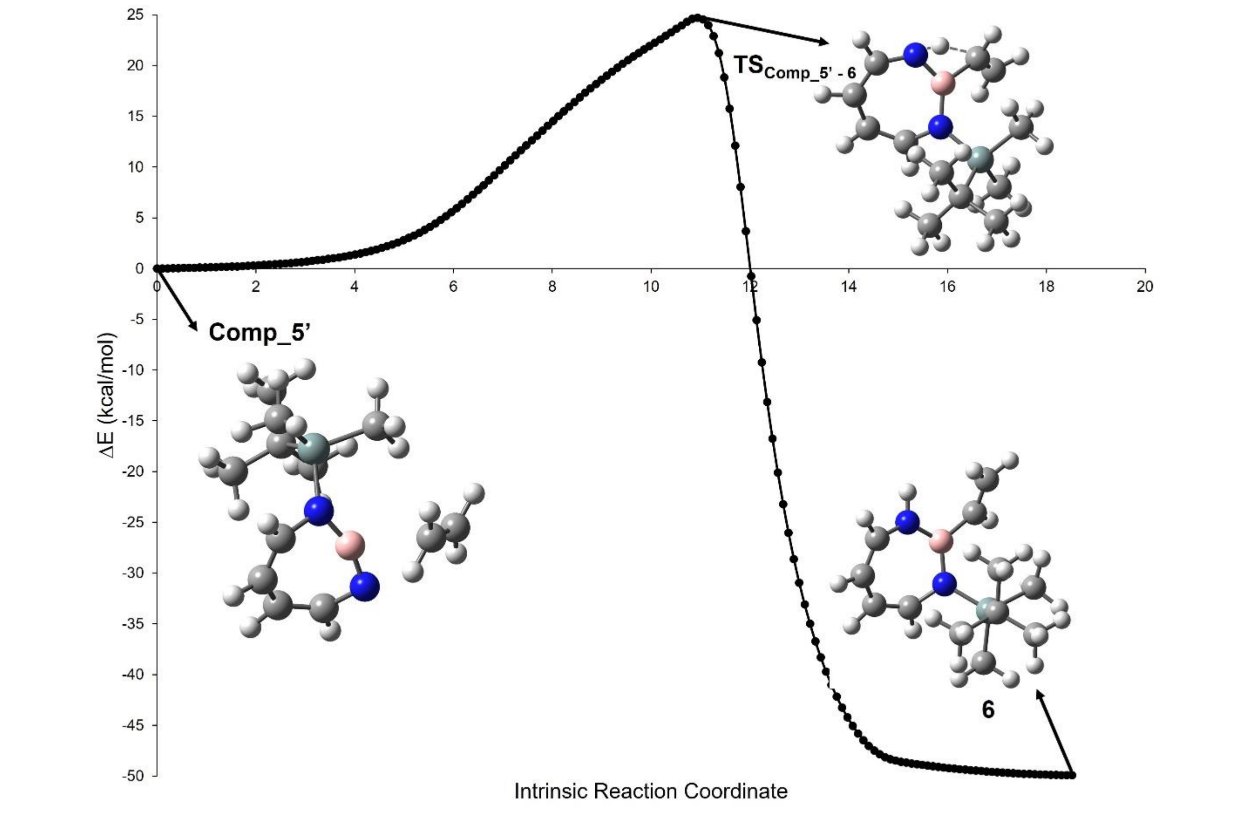

Table S11. NBO analysis of the C=C→B interaction for complex **5_comp** formed at the M06-2X/6-311+G(d,p) level of theory.

	**ΔE**[(a)]	**%(C=C)**[(b)]	**%B**[(b)]	**Occ. (C=C)**[(c)]	**Occ.B**[(c)]
**5_comp**	314.8	78.4	20.6	1.589	0.497

(a) NBO second order perturbation interaction energy associated with the R→B interaction, in kcal mol^{-1}. (b) Percentage of the donor and acceptor NBO in the corresponding NLMO. (c) Occupancy of the donor and acceptor NBO orbitals.

Table S12. NBO plots for the (a) donor NBO, (b) acceptor NBO and (c) corresponding NLMO associated with the C=C→B interaction for complex **5_comp** at the M06-2X/6-311+G(d,p) level of theory.

(a) (b) (c)

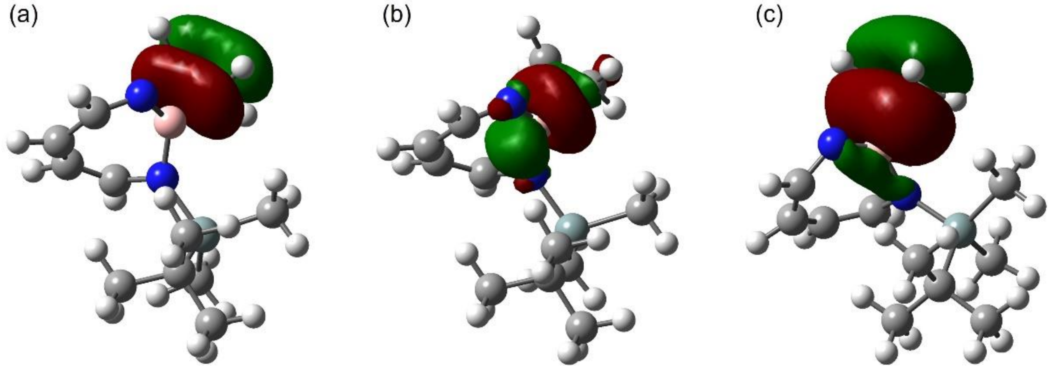

Table S13. Infrared spectroscopic data of experimental and computed (B3LYP-D3(BJ)/6-311+G(d,p)) vibrational frequencies of **3** in argon matrix.

Vibrational Mode[a]	Experimental		Computational		Assignments
	ν (cm^{-1})	I[b]	ν (cm^{-1})	I[b]	
99	3079	0.012	3246	0.007	CH str. (ring)
98	3043	0.008	3193	0.006	CH str. (ring)
97	3007	0.006	3155	0.017	CH str. (ring)
96			3135	0.016	CH str. (ring)
91	2961	0.070	3102	0.072	CH str. (tert-butyl grp)
89			3096	0.021	CH asym. str. (tert-butyl grp)
88	2939	0.028	3076	0.053	CH sym. str. (tert-butyl grp)
87	2935	0.050	3071	0.015	CH sym. str. (tert-butyl grp)
85	2903	0.016	3036	0.011	CH str. (methyl grp)
83	2888	0.012	3016	0.07	CH sym. str. (tert-butyl grp)
82	2864	0.054	3010	0.023	CH sym. str. (tert-butyl grp)
80	2141	1.000	2266	1.000[c]	N3 str.
79	1612	0.262	1647	0.117	ring str.
78	1513	0.431	1545	0.129	ring str.
77	1474	0.093	1520	0.009	CH scissor (tert-butyl grp)
75	1466	0.063	1511	0.016	CH scissor (tert-butyl grp)
71	1457	0.070	1483	0.083	ring breathing
66 [^{10}B]	1409	0.062	1447 [^{10}B]	0.055	CH bend (ring), BN str.
66	1402	0.102	1438	0.091	CH bend (ring), BN str.
65	1399	0.209	1433	0.166	CH wag (tert-butyl grp), CH bend (ring)
64	1363	0.020	1408	0.006	CH wag (tert-butyl grp)
62	1327	0.054	1396	0.176	CH bend (ring), N3 str.
61 [^{10}B]	1291	0.016	1310 [^{10}B]	0.021	CH bend (ring)
61	1283	0.206	1308	0.097	CH bend (ring)
60	1262	0.053	1299	0.013	CH wag (methyl grp)
59	1255	0.022	1293	0.046	CH wag (methyl grp)
58 [^{10}B]	1251	0.054	1260 [^{10}B]	0.016	CH wag (tert-butyl grp), BN str.
58	1232	0.086	1234	0.006	CH wag (tert-butyl grp), BN str.
57	1225	0.039	1226	0.039	CH wag (tert-butyl grp), ring str.

54 [^{10}B]	1174	0.016	1193 [^{10}B]	0.007	CH bend (ring)
54	1162	0.034	1186	0.006	CH bend (ring)
53 [^{10}B]	1151	0.043	1181 [^{10}B]	0.014	ring str., BN str
53	1142	0.257	1171	0.088	ring str., BN str
52 [^{10}B]	1086	0.016	1118 [^{10}B]	0.013	ring deformation, BN str
52	1072	0.074	1103	0.081	ring deformation, BN str
50	1016	0.068	1032	0.013	CH bend (ring)
49	1004	0.007	1025	0.003	CH wag (tert-butyl grp)
47	988	0.161	1011	0.065	ring str.
44	938	0.012	949	0.002	CH twist (tert-butyl grp)
43	935	0.004	939	0.006	CH wag (tert-butyl grp)
42	823	0.168	870	0.065	CH rock (methyl grp)
41	811	0.318	849	0.231	CH wag (methyl grp)
38	791	0.145	804	0.061	CH twist (methyl grp)
36	764	0.034	771	0.02	ring str., N3 bend
35	739	0.135	752	0.061	CH wag (ring)
34	701	0.060	706	0.001	CH wag (methyl grp)
32	684	0.054	695	0.019	ring breathing, N3 bend
31	669	0.017	669	0.003	ring breathing, N3 bend, CH wag(methyl grp)
30	614	0.028	630	0.008	ring deformation
[a] The numbering of the vibrational modes is according to calculated spectrum. [b] Intensity relative to the strongest band. [c] Computed absolute intensity: 880.3 km mol^{-1}.					

Table S14. Combination and overtone bands for **3** observed in experimental and computed (B3LYP-D3(BJ)/6-311+G(d,p)) spectra.

**Computational**		**Experimental**
**Combination/overtone**	**ν (cm-1)**	**ν (cm-1)**
ν30 + ν36	1379	1366
2ν32	1371	1341
ν31 + ν32	1344	1313
ν4 + ν57	1268	1255
2ν29	1176	1179
ν23 + ν32	1095	1079
ν15+ ν30	862	846

Table S15. Infrared spectroscopic data of experimental and computed (B3LYP-D3(BJ)/6-311+G(d,p)) vibrational frequencies of **2** in argon matrix.

Vibrational Mode	Experimental		Computational		Assignments
	ν (cm^{-1})	I[b]	ν (cm^{-1})	I[b]	
74 [^{10}B]	1809	0.161	1848 [^{10}B]	0.248	BN str.
74	1751	1.000	1788	1.000[c]	BN str.
73[e]	1587	0.014	1631	0.022	C=C str. (ring)
72	1526	0.123	1544	0.116	C=C str. (ring)
71[e]	1476	-	1518	0.016	CH scissor (tert-butyl grp)
69[e]	1468	-	1519	0.065	CH scissor (tert-butyl grp)
61[d]	1393	0.123	1436	0.043	CH bend (ring)
60	1378	0.018	1430	0.014	CH wag (tert-butyl grp)
59[d]	1365	0.006	1399	0.011	CH str.(tert-butyl grp)
58[d]	1364	0.008	1398	0.013	CH scissor (tert-butyl grp)
57[d]	1345	0.008	1372	0.029	CH bend (ring)
56	1293	0.006	1306	0.070	CH wag (methyl grp)
55	1265	0.017	1299	0.121	CH wag (methyl grp)
54	1257	0.132	1283	0.108	CH bend (ring)
53	1219	0.031	1248	0.029	CH bend (ring)
49	1155	0.006	1162	0.060	ring str.
48	1050	0.567	1057	0.417	ring str.
46	1008	0.009	1027	0.025	CH twist (tert-butyl grp, methyl grp)
45	1005	0.009	1023	0.023	ring breathing
40	924	0.013	948	0.055	CH wag (ring), CH twist (tert-butyl grp)
39	900	0.005	918	0.017	CH bend (ring)
38	857	0.005	878	0.025	ring deformation
37	840	0.385	867	0.226	CH twist (methyl grp)
36	809	0.232	844	0.376	CH rock (methyl grp)
34	796	0.056	810	0.016	CH wag (ring)
33	787	0.005	798	0.123	CH wag (methyl grp)
31	736	0.101	751	0.144	CH wag (ring)
30	688	0.120	701	0.006	CH twist (methyl grp)

29	684	0.010	690	0.064	CH wag (ring), CH wag (methyl grp)
28	662	0.011	674	0.026	ring str., CH wag (methyl grp)
27	600	0.031	614	0.030	ring breathing, CH twist (tert-butyl grp, methyl grp)
25	504	0.077	522	0.110	ring str., CH str. (tert-butyl grp)
[a] The numbering of the vibrational modes is according to calculated spectrum. [b] Intensity relative to the strongest band. [c] Computed absolute intensity: 352.9 km mol^{-1}. [d] these peaks are observed in the irradiation spectrum see Figure1 but are not visible in the difference spectrum as their intensity is low. [e] these peaks are only observed in the experiment with C_2H_4 but not observed in with CO and Ar due to the overlapping of peaks of **3** and **4** in the same region.					

Table S16. Infrared spectroscopic data of experimental and computed (B3LYP-D3(BJ)/6-311+G(d,p)) vibrational frequencies of **4** in argon matrix.

Vibrational Mode	Experimental		Computational		Assignments
	ν (cm^{-1})	I^{b}	ν (cm^{-1})	I^{b}	
80	2112	1.000	2146	1.000^{c}	CO str.
79	1595	0.029	1608	0.045	C=C str. (ring)
74 [^{10}B]	1474	0.030	1502 [^{10}B]	0.025	BN str., C=C str. (ring)
71	1467	0.074	1484	0.113	BN str., C=C str. (ring)
69	1426	0.014	1469	0.013	CH Scissor (methyl grp)
65	1391	0.003	1430	0.006	CH wag (tert-butyl grp)
63	1363	0.002	1400	0.005	CH wag (tert-butyl grp)
60	1254	0.028	1294	0.039	CH wag (methyl grp)
58	1232	0.068	1265	0.028	CH bend (ring)
54 [^{10}B]	1128	0.006	1159 [^{10}B]	0.005	ring str.
54	1122	0.014	1154	0.024	ring str.
53	1063	0.068	1102	0.093	ring str.
51	1005	0.018	1029	0.041	ring str. ,CH wag (tert-butyl grp)
52 [^{10}B]			1042 [^{10}B]	0.017	ring str. ,CH wag (tert-butyl grp)
49	945	0.007	977	0.012	CH twist (ring)
48	934	-	964	0.008	CH twist (ring)
41	835	0.034	860	0.060	CH twist (methyl grp)
40	822	-	836	0.097	CH wag (ring), CH twist (methyl grp)

38	805	0.026	801	0.028	CH wag (ring)
37	778	0.016	790	0.042	CH wag (methyl grp))
35	733	0.025	751	0.073	CH wag (ring)
33	671	0.015	672	0.021	CH wag (methyl grp)
31	588	0.014	599	0.027	ring str., CH twist (methyl grp, tert-butyl grp)
[a] The numbering of the vibrational modes is according to calculated spectrum. [b] Intensity relative to the strongest band. [c] Computed absolute intensity: 1064.2 km mol^{-1}.					

Table S17. Infrared spectroscopic data of experimental and computed (B3LYP-D3(BJ)/6-311+G(d,p)) vibrational frequencies of **4** ($C^{18}O$) in argon matrix.

Vibrational Mode	Experimental		Computational		Assignments
	ν (cm^{-1})	I[b]	ν (cm^{-1})	I[b]	
80	2065	1.000	2099	1.000[c]	CO str.
79	1595	0.047	1608	0.046	C=C str. (ring)
74 [^{10}B]	1474	0.045	1502 [^{10}B]	0.027	BN str., C=C str. (ring)
71	1467	0.112	1483	0.120	BN str., C=C str. (ring)
69	1424	0.011	1469	0.014	CH Scissor (methyl grp)
65	1391	0.002	1430	0.006	CH wag (tert-butyl grp)
63	1363	0.003	1400	0.005	CH wag (tert-butyl grp)
60	1254	0.031	1294	0.041	CH wag (methyl grp)
58	1233	0.053	1265	0.029	CH bend (ring)
54 [^{10}B]	1126	0.008	1159 [^{10}B]	0.005	ring str.
54	1121	0.014	1154	0.025	ring str.
53	1063	0.074	1102	0.097	ring str.
51	1005	0.024	1029	0.043	ring str. ,CH wag (tert-butyl grp)
52 [^{10}B]			1042 [^{10}B]	0.018	ring str. ,CH wag (tert-butyl grp)
49	945	0.006	977	0.012	CH twist (ring)
48	938	0.005	964	0.008	CH twist (ring)
41	835	0.047	860	0.063	CH twist (methyl grp)
40	821	-	836	0.100	CH wag (ring), CH twist (methyl grp)
38	805	0.036	801	0.029	CH wag (ring)
37	778	0.030	790	0.044	CH wag (methyl grp))

35	732	0.025	751	0.076	CH wag (ring)
33	671	0.023	672	0.022	CH wag (methyl grp)
31	590	0.011	598	0.029	ring str., CH twist (methyl grp, tert-butyl grp)
[a] The numbering of the vibrational modes is according to calculated spectrum. [b] Intensity relative to the strongest band. [c] Computed absolute intensity: 1024.2 km mol^{-1}					

Table S18. Infrared spectroscopic data of experimental and computed (B3LYP-D3(BJ)/6-311+G(d,p)) vibrational frequencies of **4** (^{13}CO) in argon matrix.

Vibrational Mode	Experimental		Computational		Assignments
	ν (cm^{-1})	I^b	ν (cm^{-1})	I^b	
80	2064	1.000	2096	1.000[c]	CO str.
79	1595	0.046	1608	0.047	C=C str. (ring)
74 [^{10}B]	1474	0.052	1502 [^{10}B]	0.027	BN str., C=C str. (ring)
71	1467	0.128	1483	0.121	BN str., C=C str. (ring)
69	1425	0.012	1469	0.014	CH Scissor (methyl grp)
65	1392	0.004	1430	0.006	CH wag (tert-butyl grp)
63	1363	0.006	1400	0.005	CH wag (tert-butyl grp)
60	1255	0.044	1294	0.041	CH wag (methyl grp)
58	1231	0.057	1265	0.030	CH bend (ring)
54 [^{10}B]	1128	0.012	1159 [^{10}B]	0.005	ring str.
54	1125	0.019	1154	0.026	ring str.
53	1063	0.017	1102	0.099	ring str.
51	1004	0.036	1029	0.044	ring str. ,CH wag (tert-butyl grp)
52 [^{10}B]			1042 [^{10}B]	0.018	ring str. ,CH wag (tert-butyl grp)
49	944	0.007	977	0.013	CH twist (ring)
48	939	0.004	964	0.008	CH twist (ring)
41	837	0.057	860	0.064	CH twist (methyl grp)
40	822	-	836	0.102	CH wag (ring), CH twist (methyl grp)
38	805	0.046	801	0.030	CH wag (ring)
37	777	0.033	790	0.045	CH wag (methyl grp))
35	733	0.044	751	0.078	CH wag (ring)
33	671	0.026	672	0.023	CH wag (methyl grp)

31	590	0.010	596	0.032	ring str., CH twist (methyl grp, tert-butyl grp)
[a] The numbering of the vibrational modes is according to calculated spectrum. [b] Intensity relative to the strongest band. [c] Computed absolute intensity: 1004.0 km mol^{-1}					

Table S19. Isotopic shifts observed in experimental and computed (B3LYP-D3(BJ)/6-311+G(d,p)) vibrational frequencies of **4** in argon matrix.

**Experimental**	**Computational**	**Experimental**	**Computational**	**Experimental**	**Computational**
**ν (cm^{-1}) (CO)**	**ν (cm^{-1}) (CO)**	**ν (cm^{-1}) (C^{18}O)**	**ν (cm^{-1}) (C^{18}O)**	**ν (cm^{-1}) (^{13}CO)**	**ν (cm^{-1}) (^{13}CO)**
2112	2146	2065	2099	2064	2096
**Isotopic Difference**					

Table S20. Infrared spectroscopic data of experimental and computed (B3LYP-D3(BJ)/6-311+G(d,p)) vibrational frequencies of **5** in argon matrix.

**Vibrational Mode**	**Experimental**		**Computational**		**Assignments**
	**ν (cm^{-1})**	**I^{b}**	**ν (cm^{-1})**	**I^{b}**	
88	1637	0.402	1687	0.522	C=C str. (ring)
87	1615	0.982	1675	0.878	C=C str. (ring)
85	1481	0.049	1518	0.178	CH scissor (ter-butyl grp)
79	1457	0.144	1486	0.191	CH rock (ring), CH scissor (tert-butyl grp)
77	1433	0.022	1469	0.081	CH scissor (methyl grp)
74	1417	0.336	1449	0.139	CH scissor (C_2H_4)
73	1411	0.445	1443	0.492	CH rock (ring)
71 [^{10}B]	1383	0.441	1410	0.338	BN str., CH wag (C_2H_4), CH bend (ring)
71	1376	0.985	1402	1.267	BN str., CH wag (C_2H_4), CH bend (ring)
68 [^{10}B]	1337	0.151	1358	0.179	BN str., CH bend (ring)
68	1321	0.331	1338	0.137	BN str., CH bend (ring)
67 [^{10}B]	1297	0.041	1332	0.054	BN str., CH wag (C_2H_4), CH bend (ring)
67	1290	0.363	1320	0.985	BN str., CH wag (C_2H_4), CH bend (ring)
65	1254	0.333	1296	0.269	CH wag (methyl grp)

63	1207	0.297	1237	0.192	CH wag (C_2H_4), CH bend (ring), CH twist (tert-butyl grp)
58	1145	0.115	1173	0.143	CH wag (C_2H_4), CH bend (ring)
56	1105	0.066	1129	0.105	BN str., NC str., CH wag (C_2H_4)
55 [^{10}B]	1059	0.041	1076	0.107	Ring breathing, CH wag (C_2H_4)
54	1035	0.010	1040	0.063	Ring str., CH wag (C_2H_4)
52	1026	0.023	1027	0.087	CH twist (tert-butyl grp)
51	974	0.069	986	0.23	Ring str., CC str. (C_2H_4)
47	930	0.016	950	0.037	CH twist (C_2H_4), CH twist (tert-butyl grp), CH wag (ring)
43	890	0.038	908	0.045	Ring breathing, CH wag (C_2H_4), CC str. (C_2H_4)
42	839	0.578	867	0.555	CH twist (methyl grp)
41	828	1	848	1[c]	Ring str., CH twist (methyl grp), CH wag (C_2H_4)
39	798	0.469	819	0.14	Ring str., CH twist (methyl grp), CH wag (tert-butyl grp), CH wag (C_2H_4)
37	777	0.044	790	0.196	CH twist (methyl grp), CH wag (C_2H_4)
34	720	0.073	730	0.188	CH wag (ring), CH wag (C_2H_4)
33	718	0.073	722	0.223	Ring str., CH wag (ring), CH wag (C_2H_4)
31	679	0.060	679	0.160	CH str. (methyl grp)

[a] The numbering of the vibrational modes is according to calculated spectrum. [b] Intensity relative to the strongest band. [c] Computed absolute intensity: 1064.2 km mol^{-1}.

Table S21. Infrared spectroscopic data of experimental and computed (B3LYP-D3(BJ)/6-311+G(d,p)) vibrational frequencies of **5** (C_2D_4) in argon matrix.

Vibrational Mode	Experimental		Computational		Assignments
	ν (cm^{-1})	I[b]	ν (cm^{-1})	I[b]	
88	1636	0.308	1686	0.369	C=C str. (ring)
87	1615	0.930	1675	0.588	C=C str. (ring)
86	1474	0.092	1518	0.122	CH scissor (tert-butyl grp)
80	1455	0.135	1486	0.149	CH rock (ring), CH scissor (tert-butyl grp)
78	1421	0.040	1469	0.055	CH scissor (methyl grp)

75 [^{10}B]	1416	0.148	1451	0.111	CH rock (ring)
75	1411	0.190	1443	0.372	CH rock (ring)
73 [^{10}B]	1380	0.127	1401	0.228	BN str., CH bend (ring), CH wag (C_2H_4)
71	1372	1	1391	1[c]	BN str., CH bend (ring), CH wag (C_2H_4)
70 [^{10}B]	1337	0.102	1358	0.112	BN str., CH bend (ring)
70	1322	0.321	1340	0.152	BN str., CH bend (ring)
69 [^{10}B]	1298	0.098	1324	0.074	BN str., CH bend (ring)
69	1283	0.765	1311	0.709	BN str., CH bend (ring)
67	1253	0.134	1296	0.189	CH wag (methyl grp)
66	1212	0.120	1241	0.148	CH bend (ring), CH twist (tert-butyl grp)
62	1155	0.088	1181	0.171	BN str., CH wag (C_2H_4), CH bend (ring)
61	1102	0.062	1125	0.068	CH wag (C_2H_4), CH bend (ring)
60	1088	0.058	1110	0.046	BN str., NC str., CH wag (C_2H_4)
58	1041	0.325	1050	0.488	Ring breathing, CH wag (C_2H_4)
56	1008	0.014	1027	0.08	CH twist (tert-butyl grp)
54 [^{10}B]	975	0.008	987	0.038	Ring str., CC str. (C_2H_4)
54	966	0.011	983	0.112	Ring str., CC str. (C_2H_4)
46	878	0.010	895	0.017	Ring breathing, CH wag (C_2H_4), CC str. (C_2H_4)
45	840	0.330	867	0.401	CH twist (methyl grp)
43	813	0.384	844	0.709	Ring str., CH twist (methyl grp), CH wag (C_2H_4)
41	790	0.154	806	0.157	Ring str., CH twist (methyl grp), CH wag (tert-butyl grp), CH wag (C_2H_4)
38 [^{10}B]	778	0.013	782	0.024	CH twist (methyl grp), CH wag (C_2H_4)
38	767	0.099	780	0.081	CH twist (methyl grp), CH wag (C_2H_4)
35	716	-	725	0.222	CH wag (ring), CH wag (C_2H_4)
33	680	0.030	684	0.133	Ring str., CH wag (ring), CH wag (C_2H_4)

[a] The numbering of the vibrational modes is according to calculated spectrum. [b] Intensity relative to the strongest band. [c] Computed absolute intensity: 1024.2 km mol^{-1}

Table S22. Isotopic shifts observed in experimental and computed (B3LYP-D3(BJ)/6-311+G(d,p)) vibrational frequencies of **5** in argon matrix.

**Experimental**		**Computational**		**Isotopic Difference**	
**ν (cm^{-1}) (C_2H_4)**	**ν (cm^{-1}) (C_2D_4)**	**ν (cm^{-1}) (C_2H_4)**	**ν (cm^{-1}) (C_2D_4)**	**Experimental**	**Computational**
718	680	(ν33) 721	(ν33) 684	38	37
777	767	(ν37) 790	(ν38) 780	10	10
798	790	(ν39) 819	(ν41) 806	8	13
828	813	(ν41) 848	(ν43) 844	15	4
890	878	(ν43) 908	(ν46) 895	12	13
974	966	(ν51) 986	(ν54) 984	8	2
-	1041	(ν55) 1065	(ν58) 1050	-	-
1059	-	(ν55) 1076 [^{10}B]	(ν58) 1051 [^{10}B]	-	-
1145	1102	(ν58) 1173	(ν61) 1125	43	48
1290	1283	(ν67) 1320	(ν69) 1311	7	9
1376	1372	(ν71) 1401	(ν71) 1391	4	10

Table S23. Cartesian coordinates of stationary points at different level of theories.

CO							
B3LYP-D3(BJ)/6-311+G(d,p)				M06-2X/6-311+G(d,p)			
C	0.00000000	0.00000000	-0.64435700	C	0.00000000	0.00000000	-0.64123200
O	0.00000000	0.00000000	0.48326800	O	0.00000000	0.00000000	0.48092400

C_2H_4							
B3LYP-D3(BJ)/6-311+G(d,p)				M06-2X/6-311+G(d,p)			
C	0.00000000	0.00000000	0.66432900	C	0.00000000	0.00000000	0.66289000
C	0.00000000	0.00000000	-0.66432900	C	0.00000000	0.00000000	-0.66289000
H	0.00000000	0.92290900	-1.23478300	H	0.00000000	0.92339000	-1.23097700
H	0.00000000	-0.92290900	-1.23478300	H	0.00000000	-0.92339000	-1.23097700
H	0.00000000	-0.92290900	1.23478300	H	0.00000000	-0.92339000	1.23097700
H	0.00000000	0.92290900	1.23478300	H	0.00000000	0.92339000	1.23097700

2							
B3LYP-D3(BJ)/6-311+G(d,p)				M06-2X/6-311+G(d,p)			
C	-3.23970600	0.32027300	-0.94728100	C	-3.25472900	0.16390800	-0.94876400
C	-1.32800700	-0.30197100	1.51853300	C	-1.28348600	0.00504600	1.52767600
C	-3.04451500	1.03071500	0.20190200	C	-3.02134300	1.07870000	0.02942000
C	-2.35401000	0.56706800	1.40211500	C	-2.30797600	0.84334300	1.28639200
H	-3.84158400	0.76068500	-1.73988100	H	-3.85512200	0.46248300	-1.80556500
H	-0.97669100	-0.63346000	2.48949100	H	-0.89209500	-0.13317400	2.53073600
H	-3.55573000	1.98396600	0.28427900	H	-3.49540100	2.04779900	-0.07716400
H	-2.73484800	0.95588200	2.34328900	H	-2.66028000	1.40583900	2.14642300
N	-2.78462400	-0.98558200	-1.17069300	N	-2.84953600	-1.17901400	-0.92466000
N	-0.65047900	-0.88964700	0.37197300	N	-0.65802400	-0.79597400	0.49600300
B	-1.64712500	-1.11857000	-0.54373800	B	-1.69541800	-1.17045200	-0.32022200
C	1.58877700	1.01743700	-0.27573300	C	1.61387800	0.95567400	-0.36974400
C	1.94909700	-1.41546800	1.65870600	C	1.95997500	-1.30522500	1.73641900
H	1.67437500	-2.45641500	1.84910500	H	1.70106900	-2.33371300	1.99823700
H	3.03726500	-1.36839100	1.56093000	H	3.04546300	-1.24988100	1.61730900
H	1.67255800	-0.82641200	2.53648700	H	1.68957100	-0.66180600	2.57736100
C	1.43584400	-1.91179500	-1.36136100	C	1.31937500	-2.02872900	-1.21175000
H	1.13363400	-2.93811100	-1.13621400	H	0.92304100	-3.00565900	-0.92375800
H	0.88687500	-1.58710400	-2.24978400	H	0.81063900	-1.71488200	-2.12762300
H	2.49873400	-1.92585600	-1.61587500	H	2.37895300	-2.15566100	-1.44646800
C	3.06182100	1.06500700	-0.72744400	C	2.99826500	0.87559400	-1.03343600
H	3.22628500	0.49838900	-1.64819700	H	2.97434100	0.27981900	-1.95020200
H	3.35952600	2.10179600	-0.92391100	H	3.34033500	1.88161600	-1.30193800
H	3.73952700	0.67258100	0.03728700	H	3.75104500	0.44298000	-0.36591800
C	0.68942000	1.56103800	-1.40159600	C	0.59573700	1.51108100	-1.37605300
H	0.78018000	0.97182500	-2.31902100	H	0.48625900	0.85932600	-2.24916600
H	-0.36358800	1.57245900	-1.10929100	H	-0.39212200	1.63514800	-0.92326200
H	0.97671500	2.59059800	-1.64617400	H	0.92644700	2.49135600	-1.73855700
C	1.41670500	1.89546600	0.97845700	C	1.69266500	1.89447500	0.84373600
H	1.68563000	2.93312300	0.74654300	H	1.97703500	2.90119100	0.51577900
H	0.38478600	1.89306000	1.33657000	H	0.73032200	1.97458900	1.35741600
H	2.06237200	1.56655900	1.79779900	H	2.44186500	1.55957200	1.56712100
Si	1.11305600	-0.78659500	0.10275100	Si	1.09103400	-0.78378400	0.16411200

3							
B3LYP-D3(BJ)/6-311+G(d,p)				M06-2X/6-311+G(d,p)			
C	1.68701500	2.80708300	-0.28495200	C	1.66622800	2.79807500	-0.26797000
C	0.54357600	2.17273600	0.10585100	C	0.52822400	2.16082800	0.11932000
C	2.91807800	0.71402300	-0.33578700	C	2.90597500	0.70825000	-0.32504100
C	2.88436800	2.07181400	-0.50659300	C	2.86662100	2.06158000	-0.49016500
H	1.66671400	3.88066000	-0.42004900	H	1.64465200	3.87131500	-0.39997100
H	-0.35727700	2.74797600	0.27291000	H	-0.37635400	2.73348600	0.28838400
H	3.85151700	0.18862600	-0.51528400	H	3.84286600	0.18898900	-0.50563700
H	3.77166800	2.61868500	-0.81492600	H	3.75268500	2.61177600	-0.79528500
B	1.64646600	0.01916500	0.09147800	B	1.63020400	0.01017400	0.09971100
N	0.45739300	0.81364200	0.31018700	N	0.44512500	0.80234900	0.31932400
Si	-1.16579100	0.11757300	0.74469500	Si	-1.16759400	0.09544200	0.74400000
C	-0.98906900	-1.25577200	2.00573300	C	-0.99118300	-1.31410200	1.95942200
H	-1.96732200	-1.46410700	2.44961500	H	-1.93798100	-1.44943500	2.48992600
H	-0.59576000	-2.17318000	1.57123000	H	-0.72227900	-2.24991500	1.47056700
H	-0.31445700	-0.95248500	2.81072400	H	-0.21693700	-1.09304300	2.69772800
C	-2.15958400	1.50051900	1.54961300	C	-2.15538000	1.45533600	1.58565200
H	-2.46228100	2.29147700	0.86049400	H	-2.47597100	2.25743100	0.91835700
H	-3.07221500	1.07393100	1.97518000	H	-3.05588400	1.01401800	2.02135800
H	-1.60072900	1.95881800	2.37039900	H	-1.58253500	1.90063500	2.40377000
C	-2.01844500	-0.45352000	-0.86158100	C	-2.01321400	-0.43505900	-0.86589400
C	-1.23795500	-1.59354700	-1.53901600	C	-1.18106300	-1.49126300	-1.60591600
H	-1.12354100	-2.45955500	-0.88280000	H	-0.99225700	-2.37333600	-0.98849800
H	-1.77207300	-1.92366900	-2.43822600	H	-1.71640400	-1.81644500	-2.50578700
H	-0.23875700	-1.27566600	-1.84328000	H	-0.21271400	-1.09175000	-1.91868500
C	-2.12516300	0.72944400	-1.84195400	C	-2.19980600	0.78113600	-1.78469900
H	-2.62001200	0.40701700	-2.76569400	H	-2.67581500	0.47014600	-2.72187200
H	-2.71343500	1.55412600	-1.42942000	H	-2.83968700	1.54352400	-1.33111000
H	-1.14045500	1.11846000	-2.11474000	H	-1.24144200	1.24406400	-2.03997400
C	-3.43366500	-0.95309200	-0.50664900	C	-3.38743800	-1.02764700	-0.51453200
H	-3.95205500	-1.28456700	-1.41404200	H	-3.90676900	-1.33420800	-1.42972700
H	-3.40444400	-1.80329200	0.18117100	H	-3.29515400	-1.91192600	0.12340300
H	-4.04424400	-0.16868500	-0.05019600	H	-4.02674300	-0.30204300	-0.00213300
N	1.51590000	-1.43072400	0.29162000	N	1.49515000	-1.44282600	0.29724100
N	3.25425900	-2.97921200	-0.04659200	N	3.30631700	-2.88452300	-0.07378800
N	2.45287300	-2.19149900	0.10275800	N	2.46715500	-2.15830500	0.09287600

4_comp							
B3LYP-D3(BJ)/6-311+G(d,p)				M06-2X/6-311+G(d,p)			
C	-2.34416700	2.03077200	-0.94334700	C	-2.39978700	1.95309200	-0.93564700
C	-0.64739600	1.02342100	1.55313500	C	-0.61653500	1.02656100	1.51424700
C	-1.68404300	2.75318100	0.00764000	C	-1.71365000	2.70311400	-0.03372700
C	-1.14260200	2.24558200	1.26563300	C	-1.13510100	2.23460100	1.22744100
H	-2.75831700	2.55187700	-1.80430400	H	-2.82393400	2.44841300	-1.80651600
H	-0.36273900	0.75400700	2.56438900	H	-0.28074100	0.78062300	2.51694300
H	-1.65884300	3.83003900	-0.12142200	H	-1.67195500	3.77197000	-0.21035000
H	-1.15899100	2.94239600	2.09988500	H	-1.13393500	2.94849200	2.04614800
N	-2.60533000	0.65480700	-0.87037200	N	-2.68371300	0.58300400	-0.81391500
N	-0.49926100	-0.02691300	0.55913400	N	-0.50156600	-0.03355700	0.53775500
B	-1.59366400	0.09849300	-0.25831700	B	-1.63134400	0.07733600	-0.23143000
C	2.30718400	0.28883100	-0.47409300	C	2.31263300	0.31792100	-0.41290100
C	1.58730100	-1.62781500	1.89354200	C	1.54102300	-1.74820800	1.78119800
H	0.84224400	-2.32673100	2.28296700	H	0.80850500	-2.50045800	2.08285500
H	2.52885400	-2.17160700	1.77655700	H	2.50588200	-2.24680700	1.65485000
H	1.74804600	-0.85087300	2.64493800	H	1.64726500	-1.03042400	2.59842800
C	0.55214300	-2.25566700	-0.96354200	C	0.54167600	-2.14715300	-1.12813400
H	-0.22123200	-2.90462000	-0.54517600	H	-0.28573100	-2.77795800	-0.79410700
H	0.16857300	-1.83705400	-1.89826000	H	0.23508100	-1.64984700	-2.05277700
H	1.41638500	-2.87808500	-1.20934500	H	1.38623800	-2.79902200	-1.36434000
C	3.53419800	-0.51393700	-0.94918800	C	3.45686600	-0.46149600	-1.08116500
H	3.27773100	-1.22228900	-1.74186400	H	3.11658600	-0.99727100	-1.97176900
H	4.29415700	0.16640800	-1.35143100	H	4.24598100	0.23181000	-1.39402700
H	4.00028700	-1.07411100	-0.13243500	H	3.91276000	-1.18822000	-0.40040100
C	1.68813100	1.03629100	-1.66992800	C	1.67870700	1.27282100	-1.43478000
H	1.36154100	0.34979700	-2.45677700	H	1.23985200	0.73310300	-2.28036800
H	0.82773000	1.63961400	-1.36946400	H	0.89256200	1.88407100	-0.98218100
H	2.42891000	1.71192100	-2.11391400	H	2.44230600	1.94826600	-1.83779600
C	2.75323900	1.31252300	0.58728700	C	2.87861000	1.13215000	0.76030300
H	3.48649900	2.00356300	0.15408600	H	3.61776100	1.85212200	0.38985200
H	1.91401200	1.90829700	0.95359100	H	2.09700300	1.69820400	1.27514400
H	3.22829600	0.82886300	1.44564200	H	3.37966900	0.49262300	1.49287500
Si	1.02267400	-0.91203400	0.25517100	Si	1.00810000	-0.91225800	0.19485400
C	-4.16904200	-2.25609300	-0.18014400	C	-4.07677000	-2.24604100	-0.09845900
O	-3.07372500	-2.47039800	-0.00522100	O	-2.97461600	-2.41063100	0.05620000

4_comp'							
B3LYP-D3(BJ)/6-311+G(d,p)				M06-2X/6-311+G(d,p)			
C	2.65479200	-1.46222200	-1.05956800	C	2.45673000	-1.73921500	-0.84256900
C	0.90509000	-0.91266300	1.53692600	C	0.91506400	-0.71371300	1.72385700
C	2.17342300	-2.34237200	-0.13504600	C	2.00267300	-2.43866900	0.23122900
C	1.59904700	-2.00840800	1.16505100	C	1.55426100	-1.87643700	1.50656000
H	3.12988300	-1.85372000	-1.95692100	H	2.82519800	-2.29366600	-1.70319400
H	0.61788300	-0.75364100	2.57079000	H	0.69132300	-0.36880100	2.72822800
H	2.34241700	-3.39714100	-0.32472200	H	2.08285000	-3.51900700	0.18589900
H	1.77445900	-2.73230900	1.95702000	H	1.77197300	-2.46498800	2.39315900
N	2.66049600	-0.07035500	-0.91770100	N	2.56862800	-0.34382700	-0.92380500
N	0.52958500	0.14395500	0.61223400	N	0.51853400	0.17672800	0.65333100
B	1.60784600	0.29026000	-0.22841000	B	1.55909400	0.12634300	-0.24109900
C	-2.17129700	-0.65659500	-0.44702400	C	-2.10446200	-0.59468500	-0.55688400
C	-1.81960400	1.15016200	2.08256100	C	-1.88049400	1.03543000	2.09200900
H	-1.22576000	1.94728700	2.53787100	H	-1.24768800	1.71613400	2.66725200
H	-2.85317800	1.50032800	2.01187700	H	-2.86975000	1.49203900	2.00695700
H	-1.80918800	0.29201600	2.75895200	H	-1.98957000	0.10894100	2.66093300
C	-0.99951800	2.22999700	-0.70574600	C	-1.00071200	2.30816000	-0.60058400
H	-0.39425600	2.99928900	-0.22015200	H	-0.52465800	3.09362200	-0.00906600
H	-0.53349800	1.99317000	-1.66629000	H	-0.39265500	2.14972400	-1.49599300
H	-1.98441400	2.65709200	-0.91120600	H	-1.97980200	2.67076500	-0.92365900
C	-3.55186400	-0.09160500	-0.83451100	C	-3.58207500	-0.18917100	-0.66382900
H	-3.47172400	0.72398900	-1.55860600	H	-3.70910800	0.77392900	-1.16820300
H	-4.16284800	-0.87774800	-1.29383900	H	-4.13337700	-0.93743300	-1.24488000
H	-4.10276100	0.28059500	0.03503200	H	-4.05620000	-0.12386300	0.32006500
C	-1.44158300	-1.14220400	-1.71335700	C	-1.50612500	-0.73466600	-1.96469600
H	-1.28755900	-0.33259500	-2.43305400	H	-1.60356300	0.18837400	-2.54353000
H	-0.46629900	-1.57517700	-1.47728600	H	-0.44581900	-1.00832400	-1.92825800
H	-2.03556700	-1.91542000	-2.21516600	H	-2.02811500	-1.52605400	-2.51491400
C	-2.36345700	-1.84579100	0.51336900	C	-1.99482200	-1.94540600	0.16559800
H	-2.94624300	-2.63322800	0.02014800	H	-2.57733700	-2.70146900	-0.37405400
H	-1.40817800	-2.28081800	0.81553400	H	-0.95850600	-2.29110400	0.21124100
H	-2.90672600	-1.55571300	1.41734400	H	-2.38503000	-1.89850500	1.18703100
Si	-1.14584300	0.71388200	0.38749700	Si	-1.15175100	0.73310200	0.39604700
C	2.70398700	2.77504100	-0.13645200	C	2.73568200	2.63614800	-0.64054600
O	3.54221100	3.45270100	-0.46571300	O	3.59936100	3.20960600	-1.06819400

4							
B3LYP-D3(BJ)/6-311+G(d,p)				M06-2X/6-311+G(d,p)			
C	2.42502200	-1.71401100	-0.99516600	C	2.42027100	-1.65526500	-1.00224500
C	1.01210400	-0.66239000	1.60510100	C	0.97431200	-0.77341200	1.63890700
C	2.08916100	-2.40257800	0.15098800	C	2.04047200	-2.41153800	0.06569100
C	1.66288000	-1.84003100	1.40163900	C	1.61677500	-1.92686300	1.36754400
H	2.81869900	-2.29166700	-1.83046000	H	2.80245800	-2.17212900	-1.88007700
H	0.77570300	-0.36356600	2.62493800	H	0.73934800	-0.52215500	2.67178000
H	2.30141000	-3.46777000	0.15245900	H	2.18444300	-3.48390900	-0.02194100
H	1.86472300	-2.42806700	2.29248200	H	1.83061400	-2.56741900	2.21788400
N	2.49316400	-0.36771300	-1.10078800	N	2.51287000	-0.28716300	-1.02110400
N	0.58928000	0.19945200	0.57190400	N	0.57331500	0.17019700	0.65428200
B	1.74116000	0.44225300	-0.30611800	B	1.70117800	0.43747400	-0.22314400
C	-2.08821300	-0.58410500	-0.54476900	C	-2.03581800	-0.52056200	-0.64957700
C	-1.79574600	1.10223100	2.06007600	C	-1.87384800	0.82933200	2.14421300
H	-1.19562500	1.84721100	2.59038600	H	-1.28283800	1.47914700	2.79505300
H	-2.81255600	1.49475500	1.97280100	H	-2.87865500	1.25329500	2.07221700
H	-1.84276700	0.20403800	2.68096000	H	-1.95971900	-0.14611800	2.62952300
C	-0.98593400	2.32400300	-0.66306300	C	-0.99612900	2.38448300	-0.37990300
H	-0.47270900	3.12403100	-0.12207500	H	-0.51454500	3.11815000	0.27296700
H	-0.46629200	2.16759000	-1.61232100	H	-0.44296000	2.34697700	-1.32320200
H	-1.99206500	2.68178600	-0.89823600	H	-1.99691900	2.75939500	-0.60995600
C	-3.54260400	-0.10991800	-0.72104400	C	-3.51278500	-0.11426700	-0.74691700
H	-3.60579300	0.80925500	-1.31120000	H	-3.63582800	0.89044500	-1.16358800
H	-4.12772700	-0.87457100	-1.24652300	H	-4.05031600	-0.80812400	-1.40410600
H	-4.03315300	0.07137400	0.23994800	H	-4.00539800	-0.14037600	0.22973600
C	-1.46371300	-0.84916500	-1.92700000	C	-1.40948200	-0.52825200	-2.05169800
H	-1.48580500	0.04119400	-2.56227300	H	-1.50893100	0.44224700	-2.54727400
H	-0.42565000	-1.17998400	-1.84176500	H	-0.34662900	-0.78958200	-2.01367800
H	-2.02174500	-1.63640500	-2.44852500	H	-1.91069500	-1.27199000	-2.68247600
C	-2.07287100	-1.89087800	0.27025400	C	-1.93092700	-1.93223300	-0.05375000
H	-2.65379000	-2.66363700	-0.24834600	H	-2.48594100	-2.64230300	-0.67867900
H	-1.05756200	-2.27103800	0.40193200	H	-0.89102900	-2.26728600	-0.00714300
H	-2.51763400	-1.75982900	1.26132300	H	-2.35315500	-1.98377900	0.95479000
Si	-1.06914100	0.74944900	0.36197600	Si	-1.08610300	0.69928800	0.44754800
C	2.36473600	1.86965700	-0.27597900	C	2.32472300	1.92778100	-0.25113600
O	2.75080900	2.93908700	-0.26207600	O	2.76219600	2.95895700	-0.32260100

4_(2+1)							
B3LYP-D3(BJ)/6-311+G(d,p)				M06-2X/6-311+G(d,p)			
C	3.66899500	-0.43420100	-0.42701600	C	3.67512500	-0.48656700	-0.41110700
C	0.79603100	-1.75371500	0.33086200	C	0.76454400	-1.72839900	0.33285900
C	3.29689800	-1.72317000	-0.39062200	C	3.28050100	-1.76176500	-0.34919800
C	1.98871600	-2.28664000	-0.01891700	C	1.94786100	-2.28875800	0.01105700
H	4.65685100	-0.09905800	-0.71085300	H	4.67339100	-0.17276000	-0.68154500
H	0.00967900	-2.46813900	0.54836900	H	-0.04026700	-2.42590500	0.54859900
H	4.04909900	-2.45559100	-0.66303500	H	4.02226100	-2.51583400	-0.58660900
H	1.97519700	-3.37118700	-0.02795500	H	1.91036200	-3.37218800	0.01920200
N	2.71692500	0.53679900	-0.07830400	N	2.72871500	0.50789900	-0.10830300
N	0.37283100	-0.40458800	0.47719700	N	0.36368800	-0.37144300	0.45306400
B	1.36123400	0.57723600	0.26799200	B	1.37414800	0.58364400	0.22862400
C	-2.24954400	0.06225600	-0.90347100	C	-2.27787300	0.03908200	-0.88304500
C	-2.07672300	-1.26981200	1.91653100	C	-2.05241700	-1.17761800	1.96258200
H	-1.45336300	-1.39692400	2.80585200	H	-1.37021000	-1.34370500	2.80015400
H	-3.06747400	-0.94772400	2.24923200	H	-2.99305100	-0.79682000	2.36945700
H	-2.19477600	-2.24730500	1.44316400	H	-2.26603300	-2.14435900	1.50027100
C	-1.32780600	1.70628500	1.58966700	C	-1.30468800	1.78415600	1.50135000
H	-0.83318800	1.65690700	2.56362700	H	-0.82974600	1.77033000	2.48558300
H	-0.80582800	2.45513500	0.99009000	H	-0.75381400	2.49025300	0.87533500
H	-2.35002500	2.05899600	1.75285100	H	-2.32118000	2.16711300	1.62600700
C	-3.73938600	0.37910500	-0.67261400	C	-3.78180600	0.20081100	-0.61617200
H	-3.88080300	1.34899300	-0.18673100	H	-4.00578500	1.13398400	-0.08983000
H	-4.27094300	0.41431600	-1.63099900	H	-4.33151700	0.22037000	-1.56424400
H	-4.22770700	-0.38187000	-0.05669000	H	-4.17895200	-0.62783400	-0.02230600
C	-1.62760600	1.14894200	-1.79975300	C	-1.78843100	1.19036600	-1.77309000
H	-1.71647800	2.14515500	-1.35858600	H	-2.00039600	2.16623700	-1.32765600
H	-0.56723900	0.95988000	-1.99059000	H	-0.71090700	1.13045100	-1.95853700
H	-2.13491000	1.17275500	-2.77148300	H	-2.29106900	1.15355600	-2.74643500
C	-2.12139200	-1.30203100	-1.60639800	C	-2.02984000	-1.29365900	-1.60470400
H	-2.64298800	-1.27856100	-2.57069000	H	-2.59216800	-1.31931600	-2.54535900
H	-1.07771400	-1.56011500	-1.80523500	H	-0.97203300	-1.43069900	-1.84755200
H	-2.56496600	-2.11037900	-1.01746400	H	-2.35671300	-2.15114500	-1.00747000
Si	-1.33960900	0.02235900	0.76915800	Si	-1.33655000	0.06953100	0.75959600
C	2.19234900	1.84194900	0.03619800	C	2.25509800	1.82140500	-0.00251900
O	2.57680500	2.97077800	-0.09876500	O	2.68419000	2.92838900	-0.13074000

4_(2+2)							
B3LYP-D3(BJ)/6-311+G(d,p)				M06-2X/6-311+G(d,p)			
C	3.72828000	-0.10805200	-0.43733500	C	3.72550000	-0.12156500	-0.43849200
C	0.96980300	-1.59833100	0.35444600	C	0.96287200	-1.58496300	0.37861100
C	3.45644600	-1.42429600	-0.33688600	C	3.45066300	-1.42948500	-0.33258300
C	2.20094800	-2.06190000	0.03230600	C	2.18698100	-2.05532500	0.05977200
H	4.69876300	0.27429000	-0.72881900	H	4.68904700	0.26353500	-0.74871300
H	0.22711600	-2.35130600	0.58437900	H	0.21856600	-2.33542600	0.62254700
H	4.27135200	-2.10298600	-0.55905800	H	4.25458600	-2.11645000	-0.56637500
H	2.25629100	-3.14516100	0.05432700	H	2.23766500	-3.13773600	0.09963100
N	2.75213500	0.82832000	-0.16810000	N	2.75136800	0.81610700	-0.14833600
N	0.46592500	-0.27649000	0.45394900	N	0.45762600	-0.26273600	0.45996400
B	1.33194900	0.78713200	0.21442500	B	1.33352200	0.79210600	0.22105100
C	-2.19255400	-0.05019300	-0.91785400	C	-2.18598500	-0.07195900	-0.91249400
C	-1.88711800	-1.40192000	1.88411000	C	-1.88941200	-1.35352300	1.90245600
H	-1.22883300	-1.50692700	2.75080600	H	-1.19430000	-1.49282900	2.73437800
H	-2.88728300	-1.16313300	2.25693900	H	-2.85779300	-1.07012900	2.32381300
H	-1.94910800	-2.37084900	1.38410700	H	-2.02113500	-2.31528200	1.40145500
C	-1.48432200	1.63228500	1.62896500	C	-1.47881900	1.67562500	1.57041700
H	-0.98365000	1.61332700	2.60073400	H	-0.98758900	1.68245400	2.54660000
H	-1.05799600	2.45399400	1.05257200	H	-1.04203600	2.47626100	0.97156200
H	-2.54349200	1.84212800	1.80395200	H	-2.53803900	1.89770100	1.72800100
C	-3.70897100	0.09544300	-0.68287300	C	-3.70409100	-0.07009400	-0.67514400
H	-3.95716000	1.03837200	-0.18712400	H	-4.03223500	0.82374000	-0.13554200
H	-4.24033000	0.08184800	-1.64167800	H	-4.23262700	-0.08477400	-1.63510700
H	-4.10986400	-0.72213900	-0.07635700	H	-4.02721600	-0.94831600	-0.10807300
C	-1.69764500	1.11344400	-1.79620800	C	-1.79824000	1.14806800	-1.76012800
H	-1.88606200	2.08578700	-1.33437500	H	-2.11085700	2.08453800	-1.29046700
H	-0.62549200	1.04480500	-1.99827600	H	-0.71801200	1.20302300	-1.92704800
H	-2.21505400	1.09934100	-2.76268600	H	-2.28136100	1.08986400	-2.74218900
C	-1.91478200	-1.38142800	-1.64048000	C	-1.78995300	-1.35327200	-1.66051300
H	-2.43449600	-1.39875300	-2.60572200	H	-2.32522500	-1.40731500	-2.61551000
H	-0.84907800	-1.52174600	-1.83927300	H	-0.71856800	-1.37948000	-1.87914000
H	-2.26883600	-2.24258400	-1.06605300	H	-2.04384700	-2.25498800	-1.09388600
Si	-1.30050300	-0.01408000	0.76244600	Si	-1.29920000	0.00707700	0.75613900
O	1.25519900	2.17787200	0.17730200	O	1.26192800	2.17936100	0.15599000
C	2.65699600	2.17950800	-0.23054700	C	2.63611600	2.15874600	-0.26631200

$TS_{4\_comp - 4\_(2+2)}$							
**B3LYP-D3(BJ)/6-311+G(d,p)**				**M06-2X/6-311+G(d,p)**			
C	3.02606500	-1.15947000	-0.88604200	C	2.90395400	-1.24595600	-0.89427000
C	0.85095300	-0.88258700	1.40622500	C	0.81930000	-0.81932400	1.45366500
C	2.60496600	-2.08129500	0.01770200	C	2.44597100	-2.13090600	0.01989300
C	1.75603200	-1.85626200	1.17469800	C	1.65892000	-1.84195200	1.21453700
H	3.71698100	-1.46901500	-1.66564600	H	3.54039300	-1.60887000	-1.69631300
H	0.34418800	-0.85702300	2.36636700	H	0.34173700	-0.74251000	2.42746600
H	2.99754300	-3.08768900	-0.09047600	H	2.75667900	-3.16357200	-0.10015900
H	1.86697400	-2.57455900	1.98168300	H	1.76716700	-2.54950500	2.03070500
N	2.69645800	0.18355300	-0.87093000	N	2.68673100	0.12575700	-0.87338400
N	0.48674500	0.16622100	0.51080100	N	0.49293600	0.22548700	0.54065600
B	1.59490000	0.63894800	-0.21383900	B	1.61207600	0.61590800	-0.21627800
C	-2.18950000	-0.66476100	-0.53559100	C	-2.14881700	-0.67227800	-0.49513100
C	-1.89024100	1.09572500	2.03153100	C	-1.91440400	1.26125800	1.92830000
H	-1.30306700	1.88098900	2.51533400	H	-1.38445000	2.12372400	2.33933700
H	-2.92296400	1.44804600	1.95691400	H	-2.96667100	1.53575000	1.81260100
H	-1.89000000	0.22142900	2.68679100	H	-1.86719500	0.45229500	2.66127100
C	-1.12231500	2.26333200	-0.72631100	C	-1.05392200	2.21631200	-0.88663500
H	-0.54635100	3.05014200	-0.23418800	H	-0.44014400	3.01274500	-0.45975400
H	-0.66687700	2.07476200	-1.70237500	H	-0.61611800	1.93092100	-1.84785900
H	-2.13043700	2.64740900	-0.90279900	H	-2.04626500	2.62846400	-1.08531700
C	-3.60174300	-0.14701100	-0.86999900	C	-3.44160900	-0.12669900	-1.12149000
H	-3.57405000	0.70357100	-1.55679000	H	-3.23281500	0.56349000	-1.94363000
H	-4.18706800	-0.93828500	-1.35336900	H	-4.03781200	-0.95252500	-1.52647100
H	-4.14921000	0.15980500	0.02663000	H	-4.06495600	0.39656100	-0.38854500
C	-1.46873600	-1.06866100	-1.83513900	C	-1.29112000	-1.33395500	-1.58384600
H	-1.38051600	-0.22916300	-2.53112900	H	-0.98452100	-0.61810600	-2.35363900
H	-0.46340100	-1.44869900	-1.63663000	H	-0.38586900	-1.78281800	-1.16452000
H	-2.02996000	-1.85934100	-2.34726700	H	-1.86355300	-2.12576000	-2.08107400
C	-2.30826300	-1.89841800	0.37934400	C	-2.51009300	-1.71970500	0.56903500
H	-2.87947000	-2.68565100	-0.12760700	H	-3.04778100	-2.55353000	0.10234500
H	-1.32918500	-2.31277600	0.63121900	H	-1.61881100	-2.13250900	1.05079700
H	-2.83035000	-1.66632000	1.31211500	H	-3.15843500	-1.30237800	1.34509200
Si	-1.19808200	0.71427400	0.32847400	Si	-1.17769100	0.75919400	0.28146400
C	3.24115000	1.96719700	-0.13054900	C	3.27270200	1.88847400	-0.05191300
O	2.06040400	2.23658200	-0.06156700	O	2.12921900	2.23369300	-0.07065200

TS$_{4_comp' - 4}$							
B3LYP-D3(BJ)/6-311+G(d,p)				M06-2X/6-311+G(d,p)			
C	2.58305100	-1.58367600	-0.99747900	C	2.42246900	-1.78333600	-0.85645300
C	0.93978700	-0.83673600	1.60793900	C	0.96070100	-0.68714500	1.71516200
C	2.10754700	-2.39787100	-0.01200600	C	1.97195400	-2.46031900	0.23178500
C	1.58609800	-1.97478300	1.28233300	C	1.55907500	-1.87150100	1.50516500
H	3.02569300	-2.04003600	-1.88072700	H	2.77906000	-2.35874900	-1.70832500
H	0.68910800	-0.61408900	2.63994300	H	0.76399400	-0.33013600	2.72170400
H	2.24608700	-3.46569700	-0.14589600	H	2.03743700	-3.54239800	0.20200300
H	1.76400100	-2.65757000	2.10957500	H	1.77358000	-2.45466000	2.39634800
N	2.63145000	-0.19332700	-0.93818500	N	2.55523100	-0.40100100	-0.96270800
N	0.56663000	0.17779900	0.64019200	N	0.57185300	0.20696600	0.64852900
B	1.64285800	0.29541100	-0.22331900	B	1.62437100	0.20152700	-0.25816200
C	-2.11229100	-0.61672600	-0.48868900	C	-2.05792300	-0.57123800	-0.56177700
C	-1.81524300	1.09226700	2.11274200	C	-1.83329300	1.03264200	2.10020700
H	-1.22913700	1.86639700	2.61561900	H	-1.21387800	1.72519000	2.67601600
H	-2.84596600	1.44942000	2.03650300	H	-2.83197500	1.46860100	2.01672300
H	-1.82095600	0.20488100	2.75015400	H	-1.92201400	0.10387300	2.66890300
C	-0.99469600	2.29273900	-0.61978200	C	-0.98696400	2.33860400	-0.57558900
H	-0.44137800	3.07091300	-0.08791400	H	-0.47914400	3.11496800	0.00256500
H	-0.49501000	2.11492300	-1.57599500	H	-0.44122300	2.19925300	-1.51317300
H	-1.99170000	2.68456300	-0.83721700	H	-1.98289400	2.71055000	-0.82966700
C	-3.51309100	-0.07118600	-0.82737800	C	-3.52958100	-0.15065500	-0.68827900
H	-3.46635000	0.78720900	-1.50349100	H	-3.64061300	0.80916200	-1.20248900
H	-4.10686700	-0.84686400	-1.32559100	H	-4.08263700	-0.89799000	-1.26905600
H	-4.06341800	0.23302200	0.06845900	H	-4.01434300	-0.07185000	0.28948200
C	-1.38700200	-1.01072300	-1.78886700	C	-1.44369500	-0.72160800	-1.96164700
H	-1.26701900	-0.15929400	-2.46550200	H	-1.52520300	0.20066200	-2.54449500
H	-0.39705300	-1.42770900	-1.58799700	H	-0.38711000	-1.00596400	-1.91010900
H	-1.96506400	-1.77333500	-2.32447200	H	-1.96785800	-1.50848700	-2.51645300
C	-2.25706500	-1.86309200	0.40518200	C	-1.97238300	-1.92195600	0.16413800
H	-2.82558100	-2.63763100	-0.12395200	H	-2.55193900	-2.67386500	-0.38461800
H	-1.28581900	-2.28637100	0.66980600	H	-0.93983000	-2.27560200	0.22749700
H	-2.79433500	-1.63991100	1.33153700	H	-2.37997600	-1.86990500	1.17848200
Si	-1.10978000	0.72909200	0.41249600	Si	-1.09798600	0.74402200	0.40338400
C	2.48521600	2.48123700	-0.37708600	C	2.42048900	2.27682100	-0.63049600
O	3.07704800	3.32679800	-0.82949200	O	3.00200400	3.13556800	-1.05315700

TS$_{4-4_(2+1)}$							
B3LYP-D3(BJ)/6-311+G(d,p)				M06-2X/6-311+G(d,p)			
C	3.66975800	-0.32113300	-0.26074600	C	3.68290400	-0.31374600	-0.04103800
C	0.81104800	-1.74568900	0.24963100	C	0.80192400	-1.74719000	0.19487900
C	3.35233400	-1.62915800	-0.20999700	C	3.37392900	-1.61777600	0.02832000
C	2.03618600	-2.25063300	-0.00812600	C	2.04114100	-2.24728300	0.04439500
H	4.69067200	0.00003200	-0.43044200	H	4.71700500	0.00846700	-0.07410100
H	0.00896800	-2.46936700	0.34396300	H	-0.01557600	-2.46235800	0.16103100
H	4.17025200	-2.32994200	-0.34744000	H	4.20722500	-2.31278700	0.04744900
H	2.04814600	-3.33279900	-0.07990100	H	2.06065200	-3.32354700	-0.08567500
N	2.70767600	0.68305500	-0.11403600	N	2.71145700	0.69523400	-0.09653700
N	0.38278500	-0.40468900	0.42351800	N	0.37882400	-0.40742000	0.39003700
B	1.36421800	0.59372100	0.14670400	B	1.36532700	0.57739100	0.08011100
C	-2.28435500	0.07313400	-0.88755000	C	-2.28833100	0.07389500	-0.89923200
C	-2.02078500	-1.33691600	1.88749200	C	-2.00598000	-1.33536300	1.85954900
H	-1.39405400	-1.45169100	2.77606500	H	-1.34432800	-1.48122400	2.71714100
H	-3.02286300	-1.05246300	2.22065600	H	-2.98460200	-1.03042500	2.23955100
H	-2.10256800	-2.31352500	1.40524900	H	-2.13195700	-2.30071400	1.36449600
C	-1.33704000	1.65046100	1.63039100	C	-1.31587800	1.64961500	1.59246600
H	-0.70249100	1.62420100	2.52062900	H	-0.66681700	1.62216200	2.47170000
H	-0.99266900	2.47017200	0.99645200	H	-0.97962300	2.46723900	0.95044400
H	-2.35276800	1.89185000	1.95586500	H	-2.32474200	1.89588400	1.93406400
C	-3.75053300	0.45513700	-0.61067100	C	-3.75488500	0.42052200	-0.60383800
H	-3.83332800	1.43492700	-0.13135300	H	-3.85132600	1.39061600	-0.10691200
H	-4.31348900	0.50275500	-1.55056200	H	-4.32526500	0.47252000	-1.53853700
H	-4.24902900	-0.27786000	0.03068000	H	-4.23058800	-0.33503000	0.02889900
C	-1.64453300	1.13072300	-1.80593500	C	-1.68052600	1.15218200	-1.80799900
H	-1.65841200	2.12832100	-1.35836300	H	-1.70639700	2.14378200	-1.34640200
H	-0.60709000	0.88164800	-2.04679300	H	-0.64232500	0.92002300	-2.06642700
H	-2.19298100	1.19006300	-2.75356500	H	-2.24280900	1.21323400	-2.74704500
C	-2.23698500	-1.29411500	-1.59484200	C	-2.21923300	-1.27742900	-1.62575300
H	-2.77886100	-1.24515300	-2.54704200	H	-2.78058500	-1.22645800	-2.56612800
H	-1.21104900	-1.59959500	-1.81831400	H	-1.18841700	-1.54866200	-1.87347700
H	-2.70362400	-2.08145400	-0.99574700	H	-2.65416300	-2.08568300	-1.02995300
Si	-1.32077100	-0.01077600	0.75562100	Si	-1.31438700	-0.01275100	0.72616600
C	1.61839700	2.06004400	-0.13717300	C	1.53736600	2.04195400	-0.35264800
O	1.78118600	3.19219900	-0.33697800	O	1.70233500	3.13660800	-0.65165400

5_Comp'							
**B3LYP-D3(BJ)/6-311+G(d,p)**				**M06-2X/6-311+G(d,p)**			
C	-2.23111000	2.10853400	-0.97404600	C	2.17437800	-2.04386100	-1.01458100
C	-0.69868200	1.00435900	1.57458900	C	0.76285400	-1.02676200	1.62404900
C	-1.59827200	2.79589000	0.02068300	C	1.61048600	-2.75982000	-0.00568500
C	-1.14030200	2.24766900	1.29279900	C	1.23204000	-2.24751100	1.31206600
H	-2.59367200	2.66065400	-1.83928000	H	2.47218500	-2.57166500	-1.91845500
H	-0.47839800	0.70402800	2.59344100	H	0.57699000	-0.73790000	2.65366600
H	-1.54184300	3.87431400	-0.08360700	H	1.53648500	-3.83281300	-0.14296200
H	-1.18250700	2.92528500	2.14214400	H	1.35257400	-2.93204300	2.14685900
N	-2.53432100	0.74535600	-0.94468400	N	2.48814100	-0.67936500	-0.97523700
N	-0.52314900	-0.02628500	0.56449400	N	0.50930800	-0.00486900	0.63153200
B	-1.60122100	0.10487400	-0.28451000	B	1.55076200	-0.11496900	-0.25983200
C	2.27060000	0.31602400	-0.50480700	C	-2.18563800	-0.28333100	-0.62935700
C	1.61814000	-1.47263000	1.97670200	C	-1.75401200	1.05394500	2.15358300
H	0.88973600	-2.15531300	2.42278200	H	-1.03493200	1.58166700	2.78551700
H	2.56596800	-2.00975700	1.88209400	H	-2.66618000	1.65460300	2.11397100
H	1.77712900	-0.64540600	2.67254800	H	-2.00081600	0.10571100	2.63722200
C	0.62018400	-2.31522700	-0.83056500	C	-0.68953200	2.42941300	-0.41522500
H	-0.07743800	-3.00188600	-0.34624000	H	-0.09534600	3.07575600	0.23518400
H	0.16417800	-1.98139100	-1.76647300	H	-0.11894100	2.27098800	-1.33479700
H	1.52553700	-2.87397300	-1.08135900	H	-1.60936000	2.95826700	-0.67795400
C	3.53784300	-0.46658600	-0.90036800	C	-3.59283000	0.32963800	-0.68385200
H	3.32504600	-1.24412800	-1.63934100	H	-3.58402300	1.34468300	-1.09349300
H	4.27467300	0.21334000	-1.34440400	H	-4.23991000	-0.27702700	-1.32788600
H	4.01409000	-0.94151500	-0.03683800	H	-4.05692000	0.36577300	0.30631900
C	1.64061500	0.94223700	-1.76295000	C	-1.60749000	-0.37255800	-2.04957700
H	1.35332600	0.18437100	-2.49798300	H	-1.57823200	0.60460800	-2.54028800
H	0.75351800	1.53214400	-1.51916100	H	-0.59356100	-0.78789300	-2.04785100
H	2.36039500	1.61157100	-2.24926500	H	-2.22935000	-1.03285100	-2.66510300
C	2.65543000	1.43763000	0.47857000	C	-2.26504000	-1.69771700	-0.03635300
H	3.36628500	2.12382400	0.00221100	H	-2.94515900	-2.31293700	-0.63733900
H	1.78570000	2.02184800	0.78657600	H	-1.28617800	-2.18491600	-0.03410700
H	3.13615600	1.04311100	1.37846800	H	-2.64694500	-1.69307000	0.98931800
Si	1.02186900	-0.87548600	0.30100400	Si	-1.06066100	0.80522300	0.43360200
C	-3.10692900	-2.44551000	-0.76475200	C	2.92607300	2.53340700	-0.84634500
C	-3.18870300	-2.17231300	0.53554200	C	3.16628500	2.24331200	0.42805300
H	-2.71913000	-2.80254300	1.28367700	H	2.85474300	2.90222400	1.23177500
H	-2.57128300	-3.31519800	-1.13064800	H	2.41255700	3.44554700	-1.13201600
H	-3.73261200	-1.30586000	0.89540800	H	3.69112900	1.33449300	0.70483900
H	-3.56552400	-1.79904300	-1.50330800	H	3.23703800	1.86121800	-1.63840400

5_Comp							
B3LYP-D3(BJ)/6-311+G(d,p)				M06-2X/6-311+G(d,p)			
C	2.26659100	-1.67011000	-0.98550000	C	2.21834200	-1.68690600	-0.98909700
C	1.04626900	-0.38063400	1.57999900	C	1.04321400	-0.42527600	1.60506300
C	2.07372000	-2.23718700	0.26613900	C	1.96010100	-2.28400200	0.21960800
C	1.74376400	-1.55547300	1.47037400	C	1.67139000	-1.61948900	1.46174500
H	2.59008900	-2.33897700	-1.78378300	H	2.50718500	-2.33616800	-1.81452500
H	0.82141200	-0.00759000	2.57976300	H	0.87192600	-0.03796500	2.61013100
H	2.33988900	-3.28609400	0.36194100	H	2.12014300	-3.35668800	0.27169900
H	2.00804300	-2.04198300	2.40478200	H	1.93957800	-2.13930800	2.37648800
N	2.32943800	-0.35577500	-1.23609800	N	2.36760700	-0.35498800	-1.18851200
N	0.54914600	0.33718000	0.49694100	N	0.56868200	0.34752800	0.52997600
B	1.66831800	0.57705400	-0.45887800	B	1.68630400	0.54580800	-0.41757600
C	-2.10863200	-0.67441200	-0.47125900	C	-2.08147400	-0.63808800	-0.50233200
C	-1.82874700	1.12844500	2.05065600	C	-1.82178500	1.05738800	2.07665500
H	-1.24304000	1.90983900	2.54383100	H	-1.22440200	1.80030100	2.61224300
H	-2.86221900	1.47976900	1.98650000	H	-2.84556200	1.43536800	2.01464300
H	-1.82138000	0.24520400	2.69420000	H	-1.84163300	0.14416600	2.67680600
C	-1.28086600	2.29679400	-0.75285900	C	-1.25532100	2.31926300	-0.68122700
H	-0.89849600	3.18519500	-0.24363100	H	-0.92040100	3.20116500	-0.12905300
H	-0.73083700	2.18010900	-1.68955400	H	-0.66718300	2.24447700	-1.59981900
H	-2.32741400	2.48846600	-1.00530600	H	-2.29538300	2.49154500	-0.97076400
C	-3.61316700	-0.34916900	-0.51329500	C	-3.58546200	-0.32969100	-0.50560400
H	-3.82253600	0.55886000	-1.08687800	H	-3.81330900	0.61298100	-1.01281400
H	-4.16314300	-1.16910900	-0.99147600	H	-4.12901700	-1.12275600	-1.03301400
H	-4.03067400	-0.21842500	0.48940900	H	-3.98930500	-0.27551100	0.50986300
C	-1.59218600	-0.89273800	-1.90527000	C	-1.58101100	-0.77023800	-1.94792500
H	-1.76940300	-0.01899400	-2.53930100	H	-1.78934600	0.13086800	-2.53294200
H	-0.52109800	-1.10924000	-1.91808900	H	-0.50309600	-0.95981100	-1.98120200
H	-2.10852800	-1.74300400	-2.36782200	H	-2.08185200	-1.60952100	-2.44535400
C	-1.88993200	-1.96374600	0.34113900	C	-1.84035900	-1.96509000	0.23076400
H	-2.44889300	-2.79239400	-0.11149600	H	-2.40988900	-2.76754900	-0.25402700
H	-0.83612300	-2.24742100	0.36654900	H	-0.78371900	-2.24278500	0.21048200
H	-2.23931400	-1.86154500	1.37321800	H	-2.16263600	-1.91991700	1.27602200
Si	-1.14748800	0.76700000	0.33295100	Si	-1.12488400	0.75852800	0.35814500
C	2.12146100	2.20496700	-0.66486400	C	2.13784200	2.23594500	-0.69488300
C	3.17736000	1.61952100	0.06644700	C	3.13379900	1.65090100	0.07573700
H	3.17093200	1.62453500	1.14921600	H	3.07641400	1.67115700	1.15831100
H	1.42993100	2.86073400	-0.15479400	H	1.37337700	2.83769400	-0.22447900
H	4.06640400	1.25088500	-0.42017400	H	4.04128700	1.27094000	-0.36722900
H	2.27575100	2.40051500	-1.72018600	H	2.29400000	2.36281200	-1.75888700

5							
B3LYP-D3(BJ)/6-311+G(d,p)				M06-2X/6-311+G(d,p)			
C	-3.54119700	-0.46036000	0.51033300	C	-3.52184400	-0.45950700	0.52331400
C	-0.80384300	-1.68304800	-0.57736900	C	-0.80419000	-1.67949200	-0.60305400
C	-3.19302200	-1.75164200	0.38698600	C	-3.17232400	-1.74668900	0.41205300
C	-1.95134100	-2.27374400	-0.19091300	C	-1.94617200	-2.26831600	-0.21016000
H	-4.49876700	-0.17618400	0.93395900	H	-4.46371300	-0.16973100	0.97801200
H	-0.05112900	-2.34447100	-0.99005000	H	-0.06257800	-2.33762300	-1.04573000
H	-3.90972500	-2.49151600	0.72207600	H	-3.86962800	-2.48553300	0.78621500
H	-1.95345500	-3.34774700	-0.34877100	H	-1.96197600	-3.33751500	-0.39381400
N	-2.71708000	0.58289000	0.11860100	N	-2.71689800	0.57963400	0.08198600
N	-0.39276900	-0.31636000	-0.55263000	N	-0.38561000	-0.31919900	-0.55292700
B	-1.35166900	0.70060200	-0.27709900	B	-1.34758400	0.69795600	-0.28801200
C	2.18490000	-0.04746500	0.96134500	C	2.17175400	-0.05081500	0.96112700
C	2.09842900	-1.20576500	-1.92703100	C	2.10496700	-1.19313200	-1.91970900
H	1.51770100	-1.26917200	-2.85165200	H	1.49808300	-1.29324700	-2.82364300
H	3.11047900	-0.88781300	-2.19358300	H	3.09487800	-0.84191700	-2.22343800
H	2.17305200	-2.21036300	-1.50551300	H	2.22964100	-2.18653400	-1.48350900
C	1.54571400	1.75887400	-1.49790200	C	1.53417700	1.76506200	-1.47735600
H	0.96603800	1.84942700	-2.42013800	H	0.94852200	1.85631300	-2.39567000
H	1.23883200	2.56204300	-0.82736300	H	1.22408900	2.56181800	-0.79986400
H	2.59837000	1.91718400	-1.75019800	H	2.58420600	1.93118700	-1.73435200
C	3.68746800	0.25761000	0.81858300	C	3.68119900	0.19007500	0.81929100
H	3.86387800	1.26261700	0.42392800	H	3.89744800	1.17895200	0.40306600
H	4.18165200	0.19854000	1.79608400	H	4.16716400	0.13305100	1.80051300
H	4.18698700	-0.45671200	0.15740900	H	4.15166600	-0.55981800	0.17603700
C	1.54537100	0.97617900	1.91681500	C	1.57085700	1.00800400	1.89635900
H	1.66225700	2.00200100	1.55580200	H	1.73915300	2.02345200	1.52506100
H	0.47759200	0.78491900	2.05449200	H	0.49310800	0.86412700	2.02379900
H	2.01978500	0.91985900	2.90411500	H	2.03227200	0.93900200	2.88861500
C	2.00243500	-1.45843100	1.55136300	C	1.92788000	-1.44075700	1.56739500
H	2.46607100	-1.51506100	2.54398900	H	2.39169900	-1.50406700	2.55908000
H	0.94634000	-1.71737400	1.66364200	H	0.85974100	-1.64696800	1.68426200
H	2.47281200	-2.22496100	0.92877300	H	2.36144900	-2.23574100	0.95306800
Si	1.33836100	0.04956500	-0.74711300	Si	1.33830600	0.05242300	-0.74035500
C	-1.44126800	2.29503200	-0.21562100	C	-1.42684400	2.29251900	-0.20926900
H	-1.35697700	2.83726600	-1.15871800	H	-1.33579500	2.84393400	-1.14545400
C	-2.91293600	2.03559400	0.24216800	C	-2.89728500	2.02816500	0.23582400
H	-3.68336900	2.43178600	-0.42833200	H	-3.66214800	2.44400200	-0.42625700
H	-3.13211100	2.35401500	1.26780400	H	-3.11183900	2.32213200	1.26820000
H	-0.83033600	2.80208900	0.53297400	H	-0.81932000	2.78327600	0.55252800

6							
B3LYP-D3(BJ)/6-311+G(d,p)				M06-2X/6-311+G(d,p)			
C	3.03108100	-0.64497100	-0.93258700	C	-3.02845000	-0.56386200	0.92153100
C	1.03655500	-1.41145900	1.29453400	C	-1.05010100	-1.43357900	-1.27261800
C	2.70890300	-1.87640900	-0.51100100	C	-2.71054600	-1.81089200	0.55849500
C	1.96082900	-2.17889500	0.70091800	C	-1.98307500	-2.16633200	-0.65760800
H	3.66345100	-0.52394400	-1.80657000	H	-3.64486200	-0.40183700	1.80028100
H	0.59927000	-1.76986700	2.22163200	H	-0.61991900	-1.82138700	-2.19227600
H	3.09975300	-2.71205300	-1.08075000	H	-3.08653200	-2.61841200	1.17572200
H	2.19498700	-3.11756400	1.19470400	H	-2.24118500	-3.11205700	-1.12352000
N	2.64269300	0.55608600	-0.32683500	N	-2.64687200	0.60602900	0.25441400
N	0.52927400	-0.15061000	0.85911600	N	-0.52722700	-0.16969300	-0.87017000
B	1.45035700	0.86576000	0.39614800	B	-1.42867000	0.87458600	-0.44103800
C	-1.72392400	-0.28440000	-1.09996300	C	1.68982600	-0.27096600	1.10607000
C	-2.02020300	-1.32636700	1.81515100	C	1.99873900	-1.41475100	-1.75808400
H	-1.73738300	-1.19310500	2.86348300	H	1.74731900	-1.29495200	-2.81565400
H	-3.10954100	-1.24677700	1.75856500	H	3.08814800	-1.37242300	-1.67462300
H	-1.74333200	-2.33921900	1.51636900	H	1.67579000	-2.40902700	-1.44311500
C	-1.87041500	1.66889000	1.31219400	C	1.90452900	1.59504000	-1.35164000
H	-1.51716400	1.89014000	2.32272600	H	1.52251600	1.82065600	-2.35018600
H	-1.57249000	2.49087300	0.66100600	H	1.66618500	2.43436500	-0.69646800
H	-2.96402500	1.64080100	1.34293700	H	2.99378100	1.51923300	-1.42332600
C	-3.25009400	-0.15991200	-1.26330100	C	3.20023500	-0.07702100	1.30153900
H	-3.60981100	0.83752900	-0.99411500	H	3.51524900	0.93789900	1.04092700
H	-3.53487900	-0.33936100	-2.30742600	H	3.46893300	-0.24604700	2.35114700
H	-3.78759700	-0.88947100	-0.65016800	H	3.78365200	-0.77976800	0.69833800
C	-1.03280300	0.76088700	-1.99436100	C	0.93455000	0.75430100	1.96472100
H	-1.30206700	1.78408000	-1.71843700	H	1.14555400	1.78441000	1.66131000
H	0.05578200	0.67455300	-1.94562700	H	-0.14816500	0.59902200	1.91200000
H	-1.32606000	0.61221300	-3.04105200	H	1.23001000	0.64926200	3.01572500
C	-1.27333300	-1.68942400	-1.54110300	C	1.28750400	-1.68256900	1.55790400
H	-1.52270200	-1.85046500	-2.59743100	H	1.49544600	-1.80777600	2.62752500
H	-0.19408400	-1.81894600	-1.42949100	H	0.21895300	-1.86227500	1.40091400
H	-1.76923000	-2.47569400	-0.96491200	H	1.84665500	-2.45463800	1.02129700
Si	-1.23898300	-0.00367200	0.72725200	Si	1.23656900	-0.04404000	-0.72520500
C	1.17764300	2.39279800	0.64160100	C	-1.09870800	2.39714100	-0.68053600
C	1.41635200	3.35834300	-0.25198800	C	-1.30366200	3.34383700	0.23742200
H	1.79674200	3.13539300	-1.24597400	H	-1.69308500	3.10155400	1.22389700
H	1.21895400	4.40634200	-0.04643300	H	-1.07043300	4.38924000	0.05996500
H	0.75498600	2.69324700	1.59820200	H	-0.66781700	2.70345300	-1.63182900
H	3.23309900	1.34108800	-0.56004800	H	-3.22115600	1.40870400	0.46714100

$TS_{5\_comp' - 5\_comp}$							
**B3LYP-D3(BJ)/6-311+G(d,p)**				**M06-2X/6-311+G(d,p)**			
C	2.15196600	-1.95927600	-0.98840600	C	2.106024000	-1.945334000	-0.990886000
C	0.91351300	-0.73627200	1.64307700	C	0.941006000	-0.680868000	1.639829000
C	1.67027600	-2.60286900	0.11761700	C	1.642335000	-2.568340000	0.128584000
C	1.35979900	-1.98654900	1.39693200	C	1.384333000	-1.931728000	1.413925000
H	2.43353300	-2.56023000	-1.85178200	H	2.345588000	-2.561846000	-1.855645000
H	0.81583400	-0.36751200	2.65896400	H	0.859149000	-0.288955000	2.649582000
H	1.65215900	-3.68739400	0.08786900	H	1.589760000	-3.651274000	0.105551000
H	1.53125000	-2.60117600	2.27758300	H	1.586236000	-2.528563000	2.299328000
N	2.42198900	-0.60251800	-1.07506200	N	2.413222000	-0.598665000	-1.101710000
N	0.56588100	0.20402700	0.59228100	N	0.572050000	0.226970000	0.577346000
B	1.60018900	0.11618300	-0.33456300	B	1.607005000	0.122400000	-0.348718000
C	-2.13944100	-0.42616600	-0.59961700	C	-2.137060000	-0.451107000	-0.550450000
C	-1.78232800	1.04223600	2.13663300	C	-1.748497000	1.120304000	2.109276000
H	-1.14997000	1.71981200	2.71735900	H	-1.089922000	1.796487000	2.660829000
H	-2.78496200	1.47636600	2.09611500	H	-2.738765000	1.580678000	2.066423000
H	-1.85129000	0.09638300	2.67872000	H	-1.835910000	0.192577000	2.679799000
C	-0.99385900	2.45885800	-0.49016000	C	-0.986001000	2.414955000	-0.593196000
H	-0.47505000	3.20236500	0.12010700	H	-0.526512000	3.208572000	0.000849000
H	-0.46859700	2.37433500	-1.44458200	H	-0.402010000	2.289827000	-1.509249000
H	-1.99668300	2.84167600	-0.69779600	H	-1.984601000	2.752698000	-0.881926000
C	-3.57565800	0.11325700	-0.74360300	C	-3.586337000	0.049418000	-0.645773000
H	-3.60522200	1.07221600	-1.26922900	H	-3.659637000	1.006427000	-1.171124000
H	-4.18437600	-0.59293200	-1.32101800	H	-4.193231000	-0.674839000	-1.201574000
H	-4.06328500	0.24395500	0.22711000	H	-4.041362000	0.166602000	0.342454000
C	-1.51924500	-0.60621100	-1.99790600	C	-1.564807000	-0.637628000	-1.963551000
H	-1.49589700	0.33225000	-2.55963400	H	-1.601375000	0.289490000	-2.543687000
H	-0.49969500	-0.99531400	-1.94142400	H	-0.527163000	-0.986004000	-1.934395000
H	-2.11157400	-1.32122000	-2.58156600	H	-2.149157000	-1.389284000	-2.507185000
C	-2.17966300	-1.79178100	0.11090900	C	-2.116935000	-1.802488000	0.178193000
H	-2.79344600	-2.49376900	-0.46688600	H	-2.751781000	-2.519423000	-0.356257000
H	-1.18241100	-2.22422500	0.20848000	H	-1.106934000	-2.217060000	0.221205000
H	-2.61947400	-1.72008500	1.11001400	H	-2.500574000	-1.722688000	1.200025000
Si	-1.09538900	0.81145100	0.40536100	Si	-1.086251000	0.818535000	0.383804000
C	2.60627100	2.45112200	-0.74468200	C	2.578825000	2.400429000	-0.771449000
C	3.36637500	1.95136300	0.23478200	C	3.343418000	1.894850000	0.199232000
H	3.18520900	2.20023700	1.27501500	H	3.169249000	2.142760000	1.241195000
H	1.79720600	3.14075400	-0.53815600	H	1.775245000	3.093790000	-0.552790000
H	4.18380100	1.27385200	0.02456600	H	4.157744000	1.216735000	-0.022881000
H	2.79707700	2.20817200	-1.78321200	H	2.763733000	2.158890000	-1.812059000

$TS_{5\_comp\ -\ 5}$							
**B3LYP-D3(BJ)/6-311+G(d,p)**				**M06-2X/6-311+G(d,p)**			
C	2.75890600	-1.40817000	-0.99995200	C	2.50012900	-1.48063500	-0.97417300
C	0.81735600	-0.77861600	1.35786400	C	0.97285000	-0.50420600	1.56035800
C	2.42323700	-2.20133700	0.06541400	C	2.30944800	-2.12432500	0.21161200
C	1.69949700	-1.81054200	1.23831800	C	1.81433600	-1.56043000	1.44217600
H	3.34335500	-1.86209300	-1.79934400	H	2.93266700	-2.05030800	-1.79409700
H	0.30558800	-0.66542400	2.31284300	H	0.62043600	-0.23948400	2.55773600
H	2.80280000	-3.21963000	0.05364900	H	2.63680900	-3.15894900	0.25844200
H	1.80236900	-2.44385200	2.11370600	H	2.07127900	-2.07569500	2.36148700
N	2.59752100	-0.05696900	-1.07650300	N	2.39797800	-0.13039800	-1.18405500
N	0.47327800	0.13563100	0.36786800	N	0.49114000	0.27507800	0.50900400
B	1.63060600	0.66658100	-0.38942600	B	1.54339200	0.67727100	-0.45788300
C	-2.30163200	-0.65844000	-0.38995500	C	-2.10781200	-0.67969700	-0.55865900
C	-1.76765900	1.38812000	1.90264100	C	-1.91510900	0.86060900	2.12517400
H	-1.10994500	2.18364400	2.26460000	H	-1.31422400	1.54702000	2.72801000
H	-2.77806500	1.80009900	1.82768100	H	-2.93062700	1.26288700	2.07857600
H	-1.79108800	0.60220800	2.66169100	H	-1.96408600	-0.09733100	2.64881900
C	-1.14698900	2.15903800	-1.01889800	C	-1.36599200	2.32039100	-0.53480200
H	-0.62040900	3.03323200	-0.63054800	H	-1.01745800	3.15985900	0.07175700
H	-0.66142900	1.86215000	-1.95181400	H	-0.79068300	2.31237100	-1.46397300
H	-2.16796500	2.46802000	-1.25850600	H	-2.41168500	2.50973500	-0.79275200
C	-3.73230700	-0.13141100	-0.60994400	C	-3.62739400	-0.47620900	-0.49074400
H	-3.76659800	0.66294300	-1.36088300	H	-3.93125500	0.49262000	-0.90014800
H	-4.38150100	-0.94168000	-0.96343900	H	-4.13815200	-1.25156800	-1.07411600
H	-4.17187800	0.25759400	0.31360700	H	-3.99744900	-0.54117100	0.53689000
C	-1.74132700	-1.19679900	-1.71946400	C	-1.64862300	-0.65614600	-2.02373900
H	-1.73181500	-0.42784000	-2.49766700	H	-1.93710100	0.27284800	-2.52470400
H	-0.72020100	-1.56820600	-1.60168900	H	-0.56171500	-0.76673200	-2.10392900
H	-2.36184100	-2.02440900	-2.08446100	H	-2.10635300	-1.48560500	-2.57591100
C	-2.34397800	-1.80521800	0.63718300	C	-1.74465000	-2.04108700	0.05190400
H	-3.00057900	-2.60651000	0.27602200	H	-2.27548000	-2.84148400	-0.47769500
H	-1.35526300	-2.23964100	0.80109600	H	-0.67160800	-2.23783100	-0.02661900
H	-2.73576000	-1.47467600	1.60387600	H	-2.02553600	-2.10503000	1.10829000
Si	-1.17769100	0.75555400	0.22932900	Si	-1.21108400	0.69608100	0.39167200
C	2.10967600	2.22436700	-0.16604000	C	2.00919000	2.27407900	-0.49883300
C	3.32107200	1.53787800	0.23557900	C	3.14407300	1.54739100	-0.01527200
H	3.42928700	1.18282600	1.25422400	H	3.23998200	1.33489700	1.04488600
H	1.54623700	2.71331400	0.62238400	H	1.43108900	2.84765900	0.21386300
H	4.20373400	1.51884300	-0.38153200	H	4.02374300	1.39477700	-0.61857100
H	2.18491100	2.81457000	-1.07501100	H	2.08912100	2.69385300	-1.49488200

TS$_{5_comp' - 6}$							
B3LYP-D3(BJ)/6-311+G(d,p)				M06-2X/6-311+G(d,p)			
C	2.76049900	-1.80321500	-1.11864400	C	2.65874300	-1.72833900	-1.30560300
C	0.82363700	-1.01930500	1.22114900	C	0.86113000	-1.03121400	1.15505300
C	2.32915600	-2.56858700	-0.06750800	C	2.19096000	-2.55180700	-0.33053000
C	1.59833600	-2.12882600	1.08744700	C	1.56359200	-2.15889500	0.91364900
H	3.34839300	-2.29648500	-1.89114500	H	3.17383200	-2.18338000	-2.14880600
H	0.34248500	-0.85646700	2.18400300	H	0.45839500	-0.87621200	2.15625500
H	2.63312900	-3.61156000	-0.07038200	H	2.38925300	-3.61224700	-0.45190800
H	1.63775200	-2.77478400	1.95898800	H	1.64831800	-2.85333600	1.74343700
N	2.64556400	-0.45029600	-1.23062300	N	2.67556300	-0.35360800	-1.27235900
N	0.55863400	-0.06029600	0.23981500	N	0.58689800	-0.01987600	0.21721400
B	1.73077900	0.33596600	-0.54191400	B	1.75827100	0.37348400	-0.55007200
C	-2.35612400	-0.56029800	-0.18999000	C	-2.31248300	-0.57464700	-0.13626800
C	-1.35278000	1.64232000	1.78586900	C	-1.32709900	1.68007000	1.75290500
H	-0.60492400	2.42222500	1.95368400	H	-0.66904100	2.54847300	1.84293000
H	-2.33619900	2.12088300	1.77582000	H	-2.35933300	2.03928200	1.79365100
H	-1.32681200	0.96289700	2.64173000	H	-1.16666000	1.04241700	2.62637500
C	-0.91916800	1.98770100	-1.24993300	C	-0.93151200	1.95723800	-1.29173600
H	-0.24099200	2.80899800	-1.01170400	H	-0.18156300	2.73443300	-1.13011900
H	-0.57087900	1.51979900	-2.17459500	H	-0.69341200	1.44563000	-2.22855500
H	-1.90655900	2.41457500	-1.44415900	H	-1.89933500	2.44992800	-1.41434200
C	-3.70371300	0.14167000	-0.44762200	C	-3.61184900	0.09518600	-0.61096900
H	-3.66360300	0.80089000	-1.31889100	H	-3.48394000	0.58716200	-1.57899100
H	-4.48424800	-0.60452600	-0.63897400	H	-4.39944800	-0.65868800	-0.72558900
H	-4.02755500	0.73668800	0.41199600	H	-3.97592900	0.84104700	0.10378200
C	-1.95130500	-1.36796000	-1.43710100	C	-1.82084100	-1.55309800	-1.21297300
H	-1.86175400	-0.73087700	-2.32199000	H	-1.59782000	-1.04052700	-2.15402100
H	-0.99445500	-1.87553500	-1.29270500	H	-0.91393600	-2.07572200	-0.89629900
H	-2.70918200	-2.12975200	-1.65716800	H	-2.59470700	-2.30213300	-1.41958900
C	-2.52052200	-1.52214100	1.00160900	C	-2.60317000	-1.35031400	1.15748000
H	-3.31818700	-2.24486000	0.78976500	H	-3.37762600	-2.10415900	0.97154400
H	-1.60802500	-2.09054200	1.19379100	H	-1.71764100	-1.87675900	1.52355800
H	-2.79496400	-0.99422300	1.91979700	H	-2.96765900	-0.69330000	1.95302900
Si	-1.01426100	0.75347000	0.16107400	Si	-0.98991000	0.75981900	0.14822500
C	2.35762800	1.86162000	-0.46435900	C	2.40790400	1.94027300	-0.33733800
C	2.35185100	2.66908700	0.63261900	C	2.28015400	2.62647100	0.81234600
H	2.58258800	3.72646900	0.57042000	H	2.44349900	3.69744900	0.86616100
H	2.60425700	2.31689000	-1.42204100	H	2.72124200	2.45823800	-1.23974800
H	2.11659100	2.27919900	1.61806300	H	1.99026700	2.12404500	1.73143300
H	3.15511300	0.82104500	-0.43584600	H	3.09707600	0.82947400	-0.31286700

M06-2X/6-311+G(d,p)

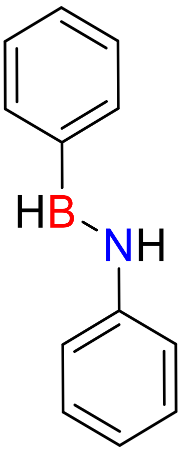

B	-0.48120100	-0.68879200	-0.00000200
N	0.53870900	0.27751100	-0.00000100
C	-1.98998100	-0.27901600	-0.00000100
C	-2.44033800	1.04962700	-0.00001100
C	-2.96194300	-1.28863200	0.00001000
C	-3.79412400	1.35767900	-0.00001000
H	-1.72946300	1.87101800	-0.00002100
C	-4.32145000	-0.99218600	0.00001100
H	-2.64197600	-2.32551000	0.00001700
C	-4.73899400	0.33368200	0.00000100
H	-4.11656700	2.39234200	-0.00001800
H	-5.05273100	-1.79200300	0.00002000
H	-5.79639900	0.57170300	0.00000200
C	1.93988500	0.12116300	0.00000000
C	2.55014100	-1.13570100	-0.00001100
C	2.74529800	1.26281800	0.00001000
C	3.93515300	-1.23502000	-0.00000900
H	1.94410900	-2.03100900	-0.00002000
C	4.12992100	1.15426800	0.00001100
H	2.27920100	2.24319400	0.00001800
C	4.73594300	-0.09663900	0.00000200
H	4.39240700	-2.21757000	-0.00001700
H	4.73449900	2.05350700	0.00002000
H	5.81509000	-0.18442700	0.00000300
H	0.26678500	1.25187600	0.00000100
H	-0.19696400	-1.84400000	0.00000000

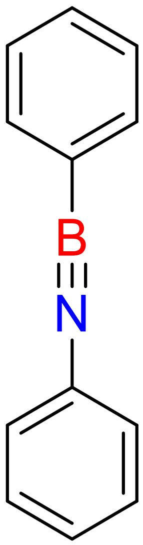

B	-0.56639100	0.00007400	0.00008500
N	0.67820300	0.00005700	0.00005800
C	-2.09100800	0.00003100	0.00003400
C	-2.80493400	1.20636000	-0.00013500
C	-2.80486700	-1.20633700	0.00016300
C	-4.19411400	1.20551800	-0.00017700
H	-2.26898400	2.14858100	-0.00023700
C	-4.19404700	-1.20557100	0.00012200
H	-2.26886500	-2.14852800	0.00029800
C	-4.88933100	-0.00004600	-0.00004800
H	-4.73474600	2.14436700	-0.00031000
H	-4.73462700	-2.14445000	0.00022400
H	-5.97299200	-0.00007600	-0.00008000
C	2.04880800	0.00002500	0.00002300
C	2.75798500	1.20739000	0.00015600
C	2.75792800	-1.20737400	-0.00014300
C	4.14617800	1.20187600	0.00012200
H	2.20321800	2.13758200	0.00028700
C	4.14612200	-1.20192500	-0.00017600
H	2.20311700	-2.13753900	-0.00024700
C	4.84824900	-0.00004100	-0.00004400
H	4.68271200	2.14347300	0.00022800
H	4.68261100	-2.14354800	-0.00030700
H	5.93126400	-0.00006700	-0.00007000

M06-2X/6-311+G(d,p)				
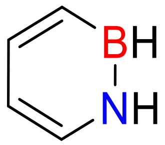	N	-1.26767000	0.50424300	-0.00008100
	B	-0.20647400	1.46774800	0.00000400
	C	1.18600600	0.87380800	-0.00001300
	C	1.32335100	-0.48849500	0.00003300
	C	0.19641700	-1.36003200	0.00009900
	C	-1.06300200	-0.84412100	-0.00004700
	H	-2.23217500	0.80327800	-0.00013100
	H	-0.50008600	2.62001400	-0.00009000
	H	2.08731500	1.47759800	0.00004400
	H	2.30950600	-0.94569500	0.00014600
	H	0.32714500	-2.43361500	0.00019900
	H	-1.94227700	-1.47698000	-0.00005200
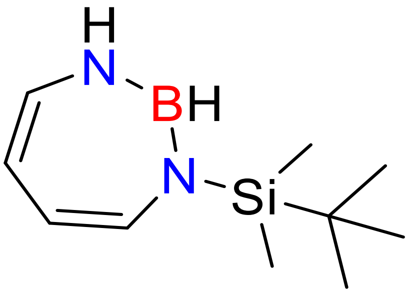	C	3.82324200	-0.64094700	-0.12100700
	C	3.78012900	0.69315100	-0.13675700
	C	2.60228000	1.52499600	-0.39142100
	C	1.31067000	1.20904200	-0.25074300
	N	0.72866700	-0.01161500	0.18286700
	B	1.35591000	-1.28485300	-0.00688600
	N	2.73464200	-1.51082700	-0.28465700
	Si	-0.96061000	0.05787300	0.73782100
	C	-2.14982200	-0.08754000	-0.73745100
	C	-1.26237300	-1.31827300	1.97403300
	C	-1.20434100	1.70879100	1.60011500
	C	-3.58535300	0.16245600	-0.25078800
	C	-1.79158600	0.95061100	-1.81069600
	C	-2.06243900	-1.48792400	-1.35981600
	H	4.77324100	-1.13978300	0.03839400
	H	4.71939000	1.21115400	0.01547800
	H	2.79959600	2.53258200	-0.74091200
	H	0.58707800	1.98022800	-0.50065800
	H	0.68141600	-2.26609700	0.07558000
	H	3.00372800	-2.47523100	-0.39765200
	H	-2.25375700	-1.20528400	2.42175200
	H	-0.52590600	-1.26779400	2.78029100
	H	-1.20402800	-2.31178800	1.52766500
	H	-0.36346600	1.91477200	2.26764600
	H	-1.29367200	2.54870400	0.90729500
	H	-2.11476700	1.68233100	2.20471600
	H	-4.28784400	0.06767200	-1.08727200
	H	-3.88762600	-0.55991500	0.51386000
	H	-3.70286700	1.16735900	0.16570900
	H	-2.49250900	0.87731800	-2.65083100
	H	-0.78388100	0.78916600	-2.20454400
	H	-1.84972100	1.97427200	-1.42722600
	H	-2.33756600	-2.26939800	-0.64570100
	H	-2.74858300	-1.56216700	-2.21201900
	H	-1.05486900	-1.70314700	-1.72692000

B3LYP/6-311+G(d,p)//M06-2X/6-311+G(d,p)				
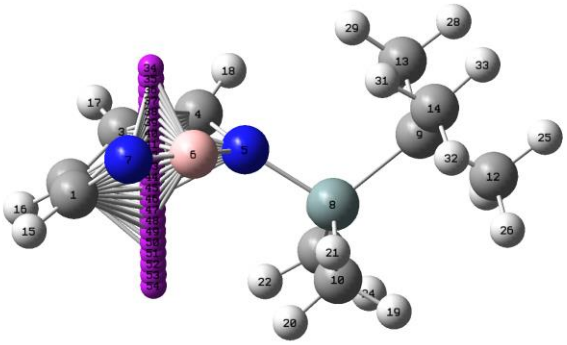	C	-3.66123600	0.76718100	-0.03605600
	C	-3.59466600	-0.54809400	0.30490100
	C	-2.61777600	-1.52129500	-0.18345200
	C	-1.33113300	-1.32225100	-0.52183400
	N	-0.66994400	-0.04092300	-0.36124900
	B	-1.62792400	0.87480800	-0.71138300
	N	-2.78945500	1.43183200	-0.90955400
	Si	0.79452400	0.18091900	0.62800300
	C	2.31157500	-0.07684300	-0.47179400
	C	0.69860000	1.93406300	1.27093400
	C	0.71357100	-1.08700100	1.99908100
	C	3.56619800	-0.17913500	0.40837400
	C	2.15282400	-1.36949200	-1.28533400
	C	2.45679600	1.10770200	-1.43725200
	H	-4.49038400	1.35943900	0.34553400
	H	-4.40798800	-0.94447400	0.90180400
	H	-2.98074100	-2.53703400	-0.31311400
	H	-0.73042900	-2.11628000	-0.95309100
	H	1.61857400	2.21077200	1.79265300
	H	-0.13234800	2.04145900	1.97248800
	H	0.54927600	2.65334800	0.46110000
	H	-0.25956600	-1.04001200	2.49445600
	H	0.84602400	-2.10436800	1.62249100
	H	1.48896600	-0.90131600	2.74654600
	H	4.45838600	-0.28073300	-0.22001200
	H	3.70430200	0.71164700	1.02971800
	H	3.52634300	-1.05014000	1.06886900
	H	3.05072300	-1.54481300	-1.88909200
	H	1.30219900	-1.30591600	-1.96996500
	H	2.01518300	-2.24556500	-0.64329900
	H	1.56016300	1.24013000	-2.05122700
	H	2.64517300	2.04356800	-0.90379400
	H	3.29969800	0.93800600	-2.11698700
	Bq	-2.26360500	0.06884900	-2.00000000
	Bq	-2.26360500	0.06884900	-1.80000000
	Bq	-2.26360500	0.06884900	-1.60000000
	Bq	-2.26360500	0.06884900	-1.40000000
	Bq	-2.26360500	0.06884900	-1.20000000
	Bq	-2.26360500	0.06884900	-1.00000000
	Bq	-2.26360500	0.06884900	-0.80000000
	Bq	-2.26360500	0.06884900	-0.60000000
	Bq	-2.26360500	0.06884900	-0.40000000
	Bq	-2.26360500	0.06884900	-0.20000000
	Bq	-2.26360500	0.06884900	0.00000000
	Bq	-2.26360500	0.06884900	0.20000000
	Bq	-2.26360500	0.06884900	0.40000000
	Bq	-2.26360500	0.06884900	0.60000000
	Bq	-2.26360500	0.06884900	0.80000000
	Bq	-2.26360500	0.06884900	1.00000000
	Bq	-2.26360500	0.06884900	1.20000000
	Bq	-2.26360500	0.06884900	1.40000000
	Bq	-2.26360500	0.06884900	1.60000000
	Bq	-2.26360500	0.06884900	1.80000000
	Bq	-2.26360500	0.06884900	2.00000000

B3LYP/6-311+G(d,p)//M06-2X/6-311+G(d,p)

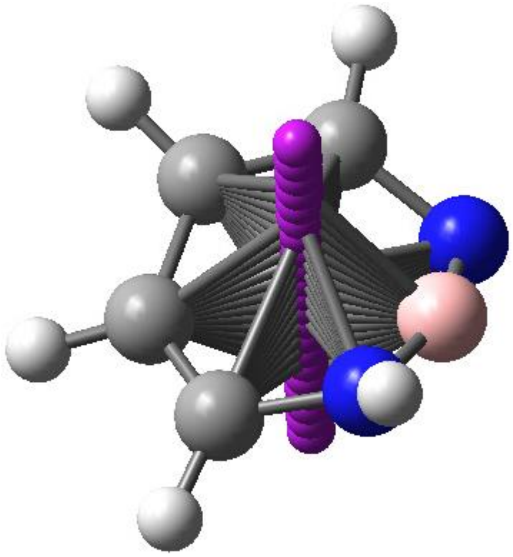

C	1.61484900	0.08542100	0.12509200
C	0.82487500	1.18443900	0.23005300
C	-0.57926500	1.30529900	-0.17110300
C	-1.54459900	0.37103200	-0.15651700
N	-1.29772900	-0.97486700	0.28693400
B	-0.00880400	-1.30738800	-0.03892000
N	1.22445900	-1.15253600	-0.41624900
H	2.65794700	0.16371200	0.42265100
H	1.30068500	2.10053200	0.56092600
H	-0.88530400	2.28267500	-0.53257500
H	-2.54063000	0.57277800	-0.53334900
H	-1.87095100	-1.36808600	1.01700200
Bq	0.00000000	0.00000000	-2.00000000
Bq	0.00000000	0.00000000	-1.80000000
Bq	0.00000000	0.00000000	-1.60000000
Bq	0.00000000	0.00000000	-1.40000000
Bq	0.00000000	0.00000000	-1.20000000
Bq	0.00000000	0.00000000	-1.00000000
Bq	0.00000000	0.00000000	-0.80000000
Bq	0.00000000	0.00000000	-0.60000000
Bq	0.00000000	0.00000000	-0.40000000
Bq	0.00000000	0.00000000	-0.20000000
Bq	0.00000000	0.00000000	0.00000000
Bq	0.00000000	0.00000000	0.20000000
Bq	0.00000000	0.00000000	0.40000000
Bq	0.00000000	0.00000000	0.60000000
Bq	0.00000000	0.00000000	0.80000000
Bq	0.00000000	0.00000000	1.00000000
Bq	0.00000000	0.00000000	1.20000000
Bq	0.00000000	0.00000000	1.40000000
Bq	0.00000000	0.00000000	1.60000000
Bq	0.00000000	0.00000000	1.80000000
Bq	0.00000000	0.00000000	2.00000000

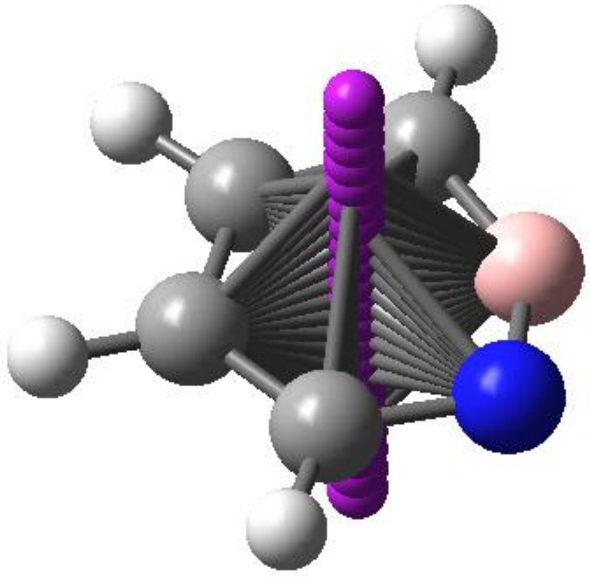

N	-1.32268300	-0.76818700	0.00000000
C	-1.22955700	0.58298900	0.00000000
C	0.00000000	1.23637800	0.00000000
C	1.20821900	0.50692200	0.00000000
C	1.28356900	-0.89329100	0.00000000
H	0.02886700	2.31790200	0.00000000
H	-2.15502600	1.15370900	0.00000000
H	2.14005800	1.06665900	0.00000000
H	2.22511000	-1.41638200	0.00000000
B	-0.11072200	-1.26851200	0.00000000
Bq	0.00000000	0.00000000	-2.00000000
Bq	0.00000000	0.00000000	-1.80000000
Bq	0.00000000	0.00000000	-1.60000000
Bq	0.00000000	0.00000000	-1.40000000
Bq	0.00000000	0.00000000	-1.20000000
Bq	0.00000000	0.00000000	-1.00000000
Bq	0.00000000	0.00000000	-0.80000000
Bq	0.00000000	0.00000000	-0.60000000
Bq	0.00000000	0.00000000	-0.40000000
Bq	0.00000000	0.00000000	-0.20000000
Bq	0.00000000	0.00000000	0.00000000
Bq	0.00000000	0.00000000	0.20000000
Bq	0.00000000	0.00000000	0.40000000
Bq	0.00000000	0.00000000	0.60000000
Bq	0.00000000	0.00000000	0.80000000
Bq	0.00000000	0.00000000	1.00000000
Bq	0.00000000	0.00000000	1.20000000
Bq	0.00000000	0.00000000	1.40000000

	Bq	0.00000000	0.00000000	1.60000000
	Bq	0.00000000	0.00000000	1.80000000
	Bq	0.00000000	0.00000000	2.00000000
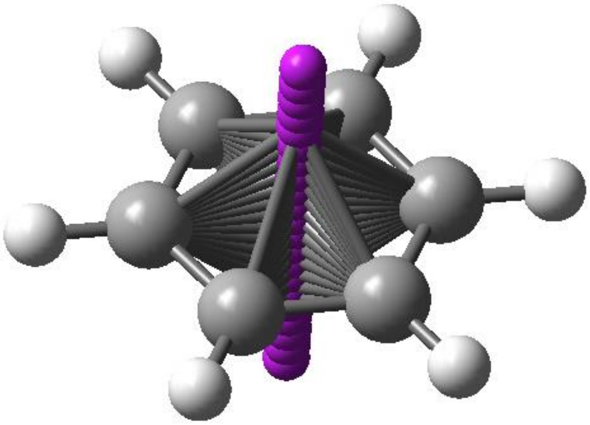	C	0.38290700	1.33759100	-0.00000100
	C	1.34986100	0.33715000	0.00002000
	C	0.96697000	-1.00037800	-0.00001900
	C	-0.38296500	-1.33757400	0.00000300
	C	-1.34984600	-0.33720700	0.00001600
	C	-0.96692800	1.00041900	-0.00001600
	H	0.68118600	2.37929800	-0.00000100
	H	2.40113100	0.59977000	0.00001000
	H	1.71990000	-1.77963500	-0.00001800
	H	-0.68110900	-2.37932000	-0.00000700
	H	-2.40115000	-0.59969700	0.00001500
	H	-1.71995600	1.77958100	-0.00002500
	Bq	0.00000000	0.00000000	-2.00000000
	Bq	0.00000000	0.00000000	-1.80000000
	Bq	0.00000000	0.00000000	-1.60000000
	Bq	0.00000000	0.00000000	-1.40000000
	Bq	0.00000000	0.00000000	-1.20000000
	Bq	0.00000000	0.00000000	-1.00000000
	Bq	0.00000000	0.00000000	-0.80000000
	Bq	0.00000000	0.00000000	-0.60000000
	Bq	0.00000000	0.00000000	-0.40000000
	Bq	0.00000000	0.00000000	-0.20000000
	Bq	0.00000000	0.00000000	0.00000000
	Bq	0.00000000	0.00000000	0.20000000
	Bq	0.00000000	0.00000000	0.40000000
	Bq	0.00000000	0.00000000	0.60000000
	Bq	0.00000000	0.00000000	0.80000000
	Bq	0.00000000	0.00000000	1.00000000
	Bq	0.00000000	0.00000000	1.20000000
	Bq	0.00000000	0.00000000	1.40000000
	Bq	0.00000000	0.00000000	1.60000000
	Bq	0.00000000	0.00000000	1.80000000
	Bq	0.00000000	0.00000000	2.00000000
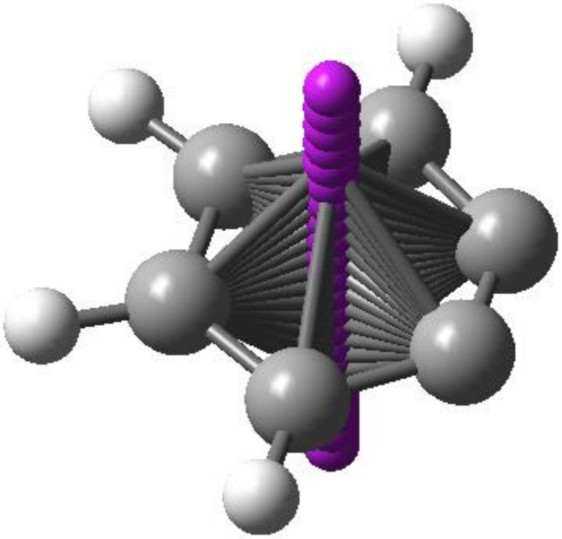	C	0.70192500	1.05194300	0.00001300
	C	-0.70191300	1.05194900	0.00001100
	C	-1.45877600	-0.13281000	0.00002600
	C	-0.61973600	-1.23033400	-0.00004200
	C	0.61972000	-1.23033300	-0.00004000
	C	1.45877700	-0.13282500	0.00003000
	H	1.22521800	2.00186800	-0.00000100
	H	-1.22519600	2.00188000	-0.00000500
	H	-2.54012000	-0.13462800	0.00000400
	H	2.54012000	-0.13465600	0.00001200
	Bq	0.00000000	0.00000000	-2.00000000
	Bq	0.00000000	0.00000000	-1.80000000
	Bq	0.00000000	0.00000000	-1.60000000
	Bq	0.00000000	0.00000000	-1.40000000
	Bq	0.00000000	0.00000000	-1.20000000
	Bq	0.00000000	0.00000000	-1.00000000
	Bq	0.00000000	0.00000000	-0.80000000
	Bq	0.00000000	0.00000000	-0.60000000
	Bq	0.00000000	0.00000000	-0.40000000
	Bq	0.00000000	0.00000000	-0.20000000
	Bq	0.00000000	0.00000000	0.00000000
	Bq	0.00000000	0.00000000	0.20000000
	Bq	0.00000000	0.00000000	0.40000000
	Bq	0.00000000	0.00000000	0.60000000
	Bq	0.00000000	0.00000000	0.80000000
	Bq	0.00000000	0.00000000	1.00000000

	Bq	0.00000000	0.00000000	1.20000000
	Bq	0.00000000	0.00000000	1.40000000
	Bq	0.00000000	0.00000000	1.60000000
	Bq	0.00000000	0.00000000	1.80000000
	Bq	0.00000000	0.00000000	2.00000000

SMD(DCM)/MN15/def2-TZVP				
**7a**	B	0.00062300	-0.00111800	0.00069500
	C	-0.42197300	-1.50492700	0.00104300
	C	-1.59280800	-1.93377900	0.64777200
	C	0.35295200	-2.48188300	-0.64553800
	C	-1.96435700	-3.27193500	0.66403600
	H	-2.21236400	-1.20461800	1.15908200
	C	-0.02848300	-3.81728500	-0.66144900
	H	1.26181600	-2.18339800	-1.15724900
	C	-1.18532200	-4.21525600	0.00132600
	H	-2.86239700	-3.57998900	1.18589600
	H	0.57677300	-4.54901500	-1.18292400
	H	-1.47909800	-5.25811600	0.00141200
	C	1.51398500	0.38600800	-0.00020500
	C	2.47295100	-0.41247000	0.64466000
	C	1.96983100	1.54723300	-0.64582700
	C	3.81697000	-0.06258500	0.65998700
	H	2.15357000	-1.31470700	1.15546500
	C	3.31631700	1.88730900	-0.66256900
	H	1.25514200	2.18433000	-1.15598900
	C	4.24153200	1.08547700	-0.00156100
	H	4.53464800	-0.68557200	1.18010500
	H	3.64500400	2.77854800	-1.18348500
	H	5.29106100	1.35450200	-0.00209100
	C	-1.09084900	1.11644400	0.00073000
	C	-0.87894600	2.34402100	0.64966200
	C	-2.32275400	0.93388600	-0.64894100
	C	-1.85262200	3.33433400	0.66489000
	H	0.06102800	2.51590500	1.16336500
	C	-3.28874900	1.93169200	-0.66637100
	H	-2.51715500	-0.00210300	-1.16191900
	C	-3.05722100	3.13170000	-0.00131200
	H	-1.67208900	4.26561300	1.18819100
	H	-4.22367000	1.77351500	-1.19041000
	H	-3.81382800	3.90729100	-0.00223100
**7b**	B	0.00017900	-0.00340600	0.00055100
	C	1.35704600	-0.77517000	0.00054000
	C	2.49334100	-0.25311000	0.63907400
	C	1.49239500	-2.01831000	-0.63800000
	C	3.70089600	-0.93544200	0.65829700
	H	2.42753700	0.70226400	1.14779400
	C	2.69813800	-2.70397500	-0.65748000
	H	0.63890000	-2.45279500	-1.14657700
	C	3.78972800	-2.15413500	0.00015000
	H	4.56511500	-0.53046100	1.16879000
	H	2.79417200	-3.65332700	-1.16841300
	C	-0.00845600	1.55758700	0.00114900
	C	1.00120500	2.29438200	-0.63834100
	C	-1.02640800	2.28254300	0.64104300
	C	0.99507500	3.68141500	-0.65753200
	H	1.80247500	1.77073100	-1.14786000
	C	-1.03613300	3.66954300	0.66083900
	H	-1.82172900	1.74956300	1.15023400
	C	-0.02442000	4.35391600	0.00177700

	H	1.77013700	4.23744800	-1.16900500
	H	-1.81744000	4.21653100	1.17254300
	C	-1.34815000	-0.78998000	-0.00002800
	C	-1.46986800	-2.03490900	0.63776500
	C	-2.49004900	-0.28012800	-0.63841800
	C	-2.66792200	-2.73394500	0.65657100
	H	-0.61169100	-2.46016600	1.14625300
	C	-3.68996700	-0.97577500	-0.65823100
	H	-2.43483800	0.67620200	-1.14659200
	C	-3.76541000	-2.19585000	-0.00096400
	H	-2.75341000	-3.68460800	1.16694000
	H	-4.55855200	-0.58002200	-1.16854200
	Cl	-0.03436900	6.08363300	0.00207700
	Cl	5.29492300	-3.00624700	-0.00072000
	Cl	-5.26103500	-3.06469700	-0.00121900
**7c**	B	0.00034500	0.00000800	-0.00067900
	C	1.12321600	1.08427500	-0.00037300
	C	2.34397600	0.85219200	-0.65115300
	C	0.93247800	2.31188300	0.65101500
	C	3.32152500	1.82390000	-0.65792200
	H	2.53806200	-0.07769900	-1.17224600
	C	1.93643600	3.25624500	0.65819400
	H	0.00956100	2.53651800	1.17223700
	C	3.13524400	3.02975000	0.00004600
	C	0.37762500	-1.51472200	-0.00070800
	C	1.53540300	-1.96472200	0.65105200
	C	-0.43401900	-2.45501300	-0.65245600
	C	1.84997100	-3.30665000	0.65803400
	H	2.19214600	-1.27884200	1.17270700
	C	-0.08268200	-3.78783600	-0.65914300
	H	-1.33537200	-2.15697400	-1.17457700
	C	1.05367600	-4.23087000	-0.00025800
	C	-1.50016800	0.43054000	-0.00042600
	C	-1.90954200	1.60293600	-0.65297200
	C	-2.46805100	-0.34694300	0.65267100
	C	-3.23963900	1.96428300	-0.65962000
	H	-1.20117900	2.23479500	-1.17537500
	C	-3.78766700	0.05118900	0.66005300
	H	-2.20158700	-1.25784200	1.17533500
	C	-4.19084900	1.20162400	0.00008300
	F	4.08876000	3.95213900	-0.00015900
	F	4.48077500	1.63040400	-1.29091600
	F	1.78184100	4.42124000	1.29136700
	F	2.93570600	-3.75634400	1.29114600
	F	1.37408900	-5.51831600	-0.00000900
	F	-0.83043900	-4.69427700	-1.29259000
	F	-3.65148900	3.06450600	-1.29346600
	F	-5.46613500	1.56739200	0.00044100
	F	-4.71935500	-0.66320700	1.29535500
**Pyridine (8a)**	C	-1.14259200	-0.71900000	-0.00021700
	C	-1.19399900	0.66871400	-0.00012500
	C	0.00014600	1.37642500	0.00010500
	C	1.19414300	0.66848300	0.00021300
	C	1.14243600	-0.71922400	0.00011800
	N	-0.00014600	-1.40906000	-0.00009000
	H	0.00020700	2.45938000	0.00016300
	H	-2.05840200	-1.30060300	-0.00037600
	H	-2.14917800	1.17688400	-0.00024500
	H	2.14946300	1.17638600	0.00034800
	H	2.05812500	-1.30101500	0.00017900
**Acetonitrile (8b)**	N	0.00000000	0.00000000	1.42906400
	C	0.00000000	0.00000000	0.27783500

	C	0.00000000	0.00000000	-1.17399100
	H	0.00000000	1.02496800	-1.54217100
	H	0.88764800	-0.51248400	-1.54217100
	H	-0.88764800	-0.51248400	-1.54217100
**Benzaldehyde (8c)**	C	-1.72052400	1.06346800	0.00000500
	C	-2.20659100	-0.23918400	-0.00000300
	C	-1.32860000	-1.32323200	-0.00000600
	C	0.03871500	-1.10445400	-0.00001100
	C	0.52993400	0.20257400	0.00000100
	C	-0.34921400	1.28360000	0.00001800
	H	-2.40735500	1.90032600	0.00001200
	H	-3.27547200	-0.41453600	-0.00000400
	H	-1.71764700	-2.33366700	-0.00000600
	H	0.73988900	-1.93043900	-0.00002000
	H	0.05020300	2.29239800	0.00003300
	C	1.98020100	0.46630400	-0.00000800
	H	2.25651900	1.53659400	-0.00021900
	O	2.83629200	-0.39314200	0.00002700
**7a●8a**	B	-0.02041000	0.01958400	-0.06134200
	C	-1.03716900	-1.15792300	-0.52390600
	C	-1.37740100	-2.28825700	0.22565500
	C	-1.72900800	-0.98291600	-1.73367300
	C	-2.33332400	-3.20620700	-0.21033500
	H	-0.90373800	-2.46835800	1.18645700
	C	-2.67834900	-1.89021700	-2.18370500
	H	-1.52043100	-0.09907300	-2.33177400
	C	-2.98386500	-3.01562100	-1.42110400
	H	-2.56982300	-4.06852600	0.40284900
	H	-3.18669000	-1.71897700	-3.12581500
	H	-3.72552900	-3.72674200	-1.76485700
	C	1.21240400	0.29677800	-1.08645600
	C	1.41187400	-0.37396900	-2.29609200
	C	2.19268600	1.23362500	-0.72198200
	C	2.51556100	-0.11619000	-3.10927100
	H	0.69658400	-1.12443300	-2.61591400
	C	3.29764600	1.50109300	-1.51960500
	H	2.08307600	1.76922900	0.21925700
	C	3.46171500	0.82487800	-2.72637200
	H	2.63576800	-0.65698800	-4.04121500
	H	4.03146400	2.23382200	-1.20348400
	H	4.32034700	1.02770000	-3.35544800
	C	-0.92495500	1.33770700	0.23995500
	C	-2.01265000	1.24245000	1.12432400
	C	-0.75653100	2.56440500	-0.40831700
	C	-2.86451900	2.31009500	1.37125200
	H	-2.19636600	0.29646800	1.62986200
	C	-1.61268800	3.64275900	-0.18170200
	H	0.05483600	2.68643500	-1.11806000
	C	-2.66601000	3.52270900	0.71378000
	H	-3.68965600	2.19746800	2.06530400
	H	-1.45365900	4.57637000	-0.70939800
	H	-3.33201200	4.35834100	0.89345800
	N	0.73760100	-0.46062900	1.30654900
	C	1.48532500	-1.57574500	1.21847400
	C	0.72301000	0.22521300	2.45586400
	C	2.22072800	-2.05085800	2.28279800
	H	1.47733000	-2.07227700	0.25658000
	C	1.44187100	-0.19011800	3.56403300
	H	0.12762500	1.12655200	2.47174600
	C	2.19917000	-1.34715400	3.48166700
	H	2.80356500	-2.95342700	2.16632900
	H	1.40290800	0.39754000	4.47017300

	H	2.77010700	-1.69386000	4.33313400
**7b●8b**	B	0.00412200	-0.00152100	0.63993700
	C	-0.43472200	1.49544100	0.20968700
	C	-1.54010600	2.11451800	0.80747600
	C	0.20894600	2.19925900	-0.81062100
	C	-1.98339600	3.37231700	0.42178900
	H	-2.08039000	1.59619600	1.59515200
	C	-0.21902000	3.45861900	-1.22339600
	H	1.06926400	1.75976300	-1.30495400
	C	-1.31192800	4.03372500	-0.59749800
	H	-2.83849600	3.83586600	0.89792700
	H	0.29138200	3.98747100	-2.01868400
	C	-1.07709400	-1.12450200	0.20347100
	C	-2.03280500	-0.89832000	-0.78944500
	C	-1.04201700	-2.40564300	0.76955700
	C	-2.91532400	-1.89216000	-1.20586700
	H	-2.09769200	0.07776800	-1.25914100
	C	-1.91423400	-3.41264900	0.37923000
	H	-0.30415400	-2.63070000	1.53498000
	C	-2.84761700	-3.14087400	-0.61188600
	H	-3.64796200	-1.69826200	-1.97930100
	H	-1.87265900	-4.39626600	0.83030900
	C	1.51381300	-0.37059100	0.18650700
	C	2.61110500	0.28303800	0.76235100
	C	1.78735100	-1.28870600	-0.82980600
	C	3.91644500	0.03530200	0.35993300
	H	2.44407400	1.01702900	1.54610900
	C	3.08613700	-1.55093900	-1.25900500
	H	0.97002300	-1.81867500	-1.30802800
	C	4.13928500	-0.88613900	-0.65420900
	H	4.75173600	0.54856100	0.81995800
	H	3.27796800	-2.26477600	-2.05039700
	Cl	-3.94540400	-4.38818000	-1.11306600
	Cl	-1.85368700	5.60602100	-1.09393900
	Cl	5.76450900	-1.20733600	-1.17126300
	N	0.01821300	-0.01078000	2.23137100
	C	0.03464700	-0.02671200	3.37543500
	C	0.05947200	-0.05249700	4.81736900
	H	0.06151300	0.96905900	5.19550100
	H	-0.82271400	-0.57766200	5.18195000
	H	0.95871300	-0.56978300	5.15030700
**7c●8c**	B	0.17808900	-0.00357100	-0.04442300
	C	0.41378300	-1.25011200	0.95892400
	C	-0.56573800	-2.19726000	1.26284400
	C	1.65249300	-1.35743100	1.60355500
	C	-0.30920600	-3.20571300	2.17491900
	H	-1.54789500	-2.19177700	0.80322500
	C	1.88613900	-2.36508700	2.51481800
	H	2.45202800	-0.65142100	1.40392800
	C	0.91224000	-3.30573800	2.81310400
	C	-0.15218900	1.38027800	0.72489300
	C	-0.21032300	1.48396600	2.11515900
	C	-0.41540100	2.52941100	-0.03443000
	C	-0.51296600	2.69301400	2.71666100
	H	-0.02096700	0.63014700	2.75600900
	C	-0.72462100	3.72249900	0.58245100
	H	-0.37597400	2.50670600	-1.11904200
	C	-0.77554000	3.82297600	1.96512700
	C	1.38786000	0.13051800	-1.10610200
	C	1.47608800	-0.75140800	-2.18951100
	C	2.41222500	1.06424200	-0.93276800
	C	2.54413600	-0.68675500	-3.05969400

	H	0.71711100	-1.50527200	-2.36684700
	C	3.47960300	1.10595600	-1.81019400
	H	2.39756200	1.77458700	-0.11322300
	C	3.56055200	0.23851900	-2.88432700
	F	1.15118600	-4.28299500	3.68708600
	F	-1.24504000	-4.11839200	2.46161800
	F	3.06559300	-2.46968100	3.13689900
	F	-0.56606300	2.79944100	4.04906400
	F	-1.06961600	4.98190200	2.55159100
	F	-0.97986300	4.82363900	-0.13258900
	F	2.63264500	-1.52119500	-4.10316400
	F	4.58827600	0.29235800	-3.73180200
	F	4.46544100	1.99676700	-1.64763900
	O	-1.07217300	-0.37885600	-0.92876600
	C	-2.25703900	-0.13421700	-0.62506400
	H	-2.46799200	0.41025100	0.30036800
	C	-3.36204600	-0.54338300	-1.44251900
	C	-4.65147400	-0.22293200	-0.99975700
	C	-3.16729000	-1.23960300	-2.64520100
	C	-5.74788700	-0.59955500	-1.75703000
	H	-4.77327500	0.31665900	-0.06748300
	C	-4.26565600	-1.61053100	-3.39392000
	H	-2.16163000	-1.47432000	-2.97115400
	C	-5.55071000	-1.29051300	-2.94849900
	H	-6.74940300	-0.35888200	-1.42653000
	H	-4.13456900	-2.14710200	-4.32423300
	H	-6.40736900	-1.58504200	-3.54201400
**1**	N	-1.32421600	-0.76236700	0.00000000
	C	-1.23140100	0.59267800	0.00000000
	C	0.00000000	1.23287200	0.00000000
	C	1.20756300	0.49979400	0.00000000
	C	1.28494700	-0.89731200	0.00000000
	H	0.03736000	2.31418300	0.00000000
	H	-2.15194700	1.16921700	0.00000000
	H	2.13787200	1.06095500	0.00000000
	H	2.22640100	-1.42114700	0.00000000
	B	-0.10936500	-1.27096800	0.00000000
**1**●pyridine	N	-1.39861400	-1.20960700	0.19692700
	C	-2.73173300	-1.23308100	0.20175400
	C	-3.53521400	-0.11143300	0.02045900
	C	-2.94054400	1.14195500	-0.18859900
	C	-1.56215400	1.29412100	-0.21020000
	H	-4.61376000	-0.21442800	0.03682300
	H	-3.22140700	-2.19608700	0.35855000
	H	-3.58904500	2.00135900	-0.34044400
	H	-1.16906800	2.29048700	-0.39447900
	B	-0.78399900	0.03501100	0.00080900
	N	0.77564800	0.02300100	0.00361700
	C	1.42702200	-1.13762100	-0.19196400
	C	1.47261400	1.15682800	0.19797200
	C	2.80477400	-1.19593500	-0.20257300
	H	0.79278200	-2.00128600	-0.33328400
	C	2.85105100	1.16438200	0.19829400
	H	0.89107200	2.05230700	0.36258600
	C	3.53164900	-0.02929900	-0.00582700
	H	3.29474400	-2.14524100	-0.36551400
	H	3.37702400	2.09400700	0.36177100
	H	4.61362300	-0.04942400	-0.00975200
**1**●acetonitrile	N	-0.71706000	-1.25219300	-0.00006000
	C	-2.05112500	-1.23967700	-0.00008300
	C	-2.82666800	-0.08245800	-0.00011000
	C	-2.21091400	1.17742400	-0.00010800

	C	-0.82897800	1.29863700	-0.00008200
	H	-3.90714500	-0.16495500	-0.00012900
	H	-2.56578600	-2.20181500	-0.00008700
	H	-2.84268000	2.06195200	-0.00012300
	H	-0.38850000	2.29132200	-0.00007600
	B	-0.09487200	0.00260000	-0.00006500
	N	1.43203600	-0.00925100	0.00001100
	C	2.57686500	-0.00792200	0.00011300
	C	4.01624300	-0.00518600	0.00028500
	H	4.37377400	0.50989000	-0.89118300
	H	4.37355200	0.51001000	0.89177300
	H	4.37377600	-1.03420000	0.00039800
**1**●benzaldehyde	N	-1.67715400	0.03767400	1.21422700
	C	-2.64621000	-0.88087300	1.25305600
	C	-3.53010300	-1.13304200	0.20800200
	C	-3.44447000	-0.38279300	-0.97674900
	C	-2.49007000	0.61045900	-1.13646700
	H	-4.28612800	-1.90121600	0.31892000
	H	-2.74719900	-1.47043200	2.16589500
	H	-4.15342400	-0.59550200	-1.77325400
	H	-2.46243600	1.16921200	-2.06718800
	B	-1.58625000	0.77774300	0.03482500
	O	-0.52074500	1.83361200	0.01560900
	C	0.72501000	1.76520900	0.10709700
	C	1.57823800	0.61726800	0.00344100
	H	1.22462600	2.72278100	0.26111600
	C	1.15290100	-0.67855900	-0.34061500
	C	2.94146300	0.87373700	0.22658500
	C	2.08646500	-1.68982300	-0.44499600
	H	0.10801100	-0.88513500	-0.52682100
	C	3.86711200	-0.14994000	0.13643700
	H	3.25430200	1.88244400	0.47013700
	C	3.43588300	-1.42907100	-0.19853000
	H	1.77038800	-2.68878400	-0.71427200
	H	4.91566200	0.04413700	0.31798200
	H	4.15616800	-2.23425200	-0.27467700
**2**	C	-3.30564700	0.08819100	-0.94675300
	C	-1.27276500	0.19137300	1.49194700
	C	-3.06131700	1.08384800	-0.05817300
	C	-2.32923000	0.97310000	1.20362500
	H	-3.90545100	0.30773600	-1.82662100
	H	-0.83690800	0.16952200	2.48539800
	H	-3.52514500	2.04410800	-0.25589700
	H	-2.67686000	1.60830200	2.01253900
	N	-2.89196300	-1.24944700	-0.78912200
	N	-0.66230900	-0.67626800	0.51791700
	B	-1.71330600	-1.14543700	-0.22206100
	C	1.67152600	0.89415800	-0.41011900
	C	1.90900500	-1.23706100	1.79314000
	H	1.64435000	-2.25287700	2.09654500
	H	2.99662400	-1.18864200	1.68577900
	H	1.62735200	-0.55659100	2.60139900
	C	1.22767900	-2.07450000	-1.10538900
	H	0.74279800	-3.00352400	-0.79312300
	H	0.76576700	-1.74803300	-2.04237300
	H	2.27670200	-2.30089000	-1.31219500
	C	3.02498000	0.70120900	-1.09967800
	H	2.93518200	0.08421500	-1.99815300
	H	3.42937700	1.67459000	-1.40196700
	H	3.75924800	0.23061100	-0.43693900
	C	0.66691400	1.46007800	-1.41431400
	H	0.46843000	0.76027500	-2.23337700

	H	-0.28827300	1.69657200	-0.93534400
	H	1.05992900	2.38379400	-1.85574700
	C	1.83909100	1.87627800	0.75026500
	H	2.15022800	2.85500900	0.36521700
	H	0.90541100	2.02053100	1.30308100
	H	2.60323200	1.53903600	1.45643800
	Si	1.07807400	-0.77547100	0.20476900
**2**●pyridine	C	0.15148100	3.06980900	-0.78180400
	C	0.63179200	1.26093800	1.66342300
	C	1.01098000	3.27543900	0.25868900
	C	0.98548700	2.55086200	1.51406900
	H	0.24976600	3.74842100	-1.63151300
	H	0.60525700	0.81659100	2.65873800
	H	1.68190600	4.12615400	0.18873000
	H	1.27700400	3.08732200	2.41335400
	N	-0.89346300	2.19573000	-0.83027500
	N	0.27294000	0.41045500	0.57877800
	B	-0.85063200	1.01829600	-0.14546200
	C	2.73570000	-0.78558400	-0.62447100
	C	1.40980300	-1.96253900	1.86617400
	H	0.49225500	-2.02895400	2.45960100
	H	1.76368900	-2.98184800	1.68769600
	H	2.16416300	-1.45240400	2.47251800
	C	0.00407500	-2.08820900	-0.86681800
	H	-0.82891400	-2.55347300	-0.33315700
	H	-0.40942500	-1.47615600	-1.67518300
	H	0.58598000	-2.89039700	-1.33080600
	C	3.53775900	-2.08575200	-0.68320900
	H	2.97619800	-2.89633200	-1.15923500
	H	4.45543200	-1.93425700	-1.26566000
	H	3.83317700	-2.42060100	0.31570700
	C	2.45482800	-0.29305400	-2.04458600
	H	1.95761800	-1.05854000	-2.64794900
	H	1.82085400	0.60171800	-2.03785500
	H	3.39435000	-0.03153100	-2.54772100
	C	3.55584700	0.27407800	0.11078600
	H	4.53876700	0.38230100	-0.36596600
	H	3.05951200	1.24776100	0.08065300
	H	3.72471100	0.01005200	1.16033600
	Si	1.09604300	-1.07718000	0.25662700
	N	-2.19851100	0.21327300	-0.09373300
	C	-3.12162800	0.41197500	-1.05269300
	C	-2.44474200	-0.64778400	0.90845800
	C	-4.32456700	-0.26069200	-1.03864900
	H	-2.84528600	1.12805000	-1.81346300
	C	-3.63518300	-1.34026300	0.98632700
	H	-1.65624700	-0.75505500	1.64149500
	C	-4.58955800	-1.14993400	-0.00398800
	H	-5.04002300	-0.08601100	-1.82930500
	H	-3.80360100	-2.01771600	1.81104900
	H	-5.52780000	-1.68800500	0.02883000
**2**●acetonitrile	C	-0.84950000	2.89186200	-1.01292400
	C	-0.10353500	1.35997700	1.56274600
	C	-0.06514400	3.31262900	0.01872500
	C	0.01562900	2.67819200	1.32116700
	H	-0.85845100	3.51748700	-1.90674100
	H	-0.06018200	0.99263600	2.58729900
	H	0.44930400	4.26093900	-0.10275600
	H	0.19200100	3.31813000	2.18154600
	N	-1.72021000	1.83719700	-1.01141700
	N	-0.29023100	0.37905400	0.54830500
	B	-1.46210400	0.74010500	-0.25377100

	C	2.29683200	-0.56041500	-0.57875700
	C	1.24466900	-1.57434100	2.10638600
	H	0.36754700	-1.70844800	2.74590800
	H	1.76344200	-2.53554200	2.04776400
	H	1.91636600	-0.86683700	2.60086400
	C	-0.17953800	-2.32899700	-0.50617300
	H	-0.96932700	-2.76460300	0.11212100
	H	-0.63533300	-1.94504500	-1.42379300
	H	0.50169900	-3.13626000	-0.79165400
	C	3.30345400	-1.70936200	-0.52755900
	H	2.87557000	-2.64799800	-0.89459700
	H	4.17304600	-1.47604500	-1.15506300
	H	3.66781600	-1.87895000	0.49018700
	C	1.89986500	-0.29073600	-2.03035700
	H	1.51768500	-1.19190600	-2.51942300
	H	1.12867600	0.48631900	-2.09560600
	H	2.76935400	0.05516500	-2.60346900
	C	2.94298300	0.69929000	-0.00257300
	H	3.86831100	0.92784100	-0.54701600
	H	2.27774700	1.56264900	-0.09454100
	H	3.20232100	0.58144600	1.05491300
	Si	0.75171900	-0.99802100	0.40594700
	N	-2.61716200	-0.28874100	-0.19558800
	C	-3.45432500	-1.06828600	-0.14669600
	C	-4.49995600	-2.05493500	-0.08473600
	H	-4.87307900	-2.11482000	0.93749100
	H	-5.30722300	-1.76454800	-0.75608100
	H	-4.09622900	-3.02167500	-0.38635200
**2**●benzaldehyde	C	0.99432400	3.18859000	-0.95702600
	C	0.23851900	1.28172700	1.36337400
	C	1.19996600	3.37036400	0.37360500
	C	0.58397200	2.57615500	1.42714400
	H	1.46440000	3.91719900	-1.62103200
	H	-0.26913000	0.82714800	2.21193100
	H	1.77570100	4.23838900	0.67882000
	H	0.36286400	3.08146400	2.36399200
	N	0.22074400	2.24180100	-1.57567700
	N	0.45085600	0.41712600	0.24567400
	B	0.02579700	1.02233300	-1.01335700
	C	3.08581000	-0.89608800	-0.07858900
	C	1.24023500	-1.50462400	2.29386700
	H	0.21370300	-1.68404700	2.62647200
	H	1.81297600	-2.41805100	2.48198400
	H	1.66247200	-0.71166000	2.91563500
	C	0.51605600	-2.48315800	-0.47828300
	H	-0.55062200	-2.57629600	-0.24902200
	H	0.63276100	-2.38291400	-1.56059200
	H	0.98994900	-3.42450300	-0.18343400
	C	3.78698900	-2.25434500	-0.09647200
	H	3.35220800	-2.92138700	-0.84681900
	H	4.84825600	-2.12574200	-0.34376700
	H	3.73343800	-2.75726800	0.87488600
	C	3.12072000	-0.29327400	-1.48374500
	H	2.52862300	-0.87546100	-2.19910900
	H	2.74312700	0.73377900	-1.48603400
	H	4.15300600	-0.26751600	-1.85511000
	C	3.81603600	0.04548500	0.87953300
	H	4.83114000	0.24441400	0.51328900
	H	3.29860600	1.00836400	0.96423900
	H	3.90293200	-0.38494900	1.88157900
	Si	1.29951600	-1.08952100	0.48256100
	O	-0.72037600	0.03896600	-1.89578100

	C	-1.77286400	-0.62068300	-1.74921100
	C	-2.78891000	-0.42905700	-0.75935800
	H	-1.92185400	-1.42883800	-2.46674000
	C	-2.84346000	0.69041300	0.08668500
	C	-3.76321600	-1.43620700	-0.67060200
	C	-3.85205400	0.77910900	1.02521900
	H	-2.12260700	1.49331200	-0.01049800
	C	-4.76077400	-1.34644900	0.28315000
	H	-3.71376800	-2.28024900	-1.34931100
	C	-4.79933800	-0.24118800	1.12886100
	H	-3.90940500	1.64079000	1.67694400
	H	-5.50829100	-2.12378900	0.36739700
	H	-5.58297000	-0.16539300	1.87259200

References

1. I. R. Dunkin, *Matrix-isolation techniques: a practical approach*, Oxford University Press, 1998.
2. Y. Zhao and D. G. Truhlar, *Theor. Chem. Acc.*, 2008, **120**, 215-241.
3. A. D. Becke, *J. Chem. Phys.*, 1993, **98**, 5648-5652.
4. C. Lee, W. Yang and R. G. Parr, *Phys. Rev. B* . 1988, **37**, 785.
5. S. Grimme, S. Ehrlich and L. Goerigk, *J. Comput. Chem.*, 2011, **32**, 1456-1465.
6. R. Krishnan, J. S. Binkley, R. Seeger and J. A. Pople, *J. Chem. Phys.*, 1980, **72**, 650-654.
7. J. Bloino and V. Barone, *J. Chem. Phys.*, 2012, **136**.
8. V. Barone, M. Biczysko and J. Bloino, *Phys. Chem. Chem. Phys.*, 2014, **16**, 1759-1787.
9. V. Barone, *J. Chem. Phys.*, 2004, **122**.
10. H. P. Hratchian and H. B. Schlegel, *J. Chem. Phys.*, 2004, **120**, 9918-9924.
11. H. Hratchian and H. Schlegel, *J. Chem. Theory Comput.*, 2005, **1**, 61-69.
12. C. Riplinger, B. Sandhoefer, A. Hansen and F. Neese, *J. Chem. Phys.*, 2013, **139**, 134101.
13. C. Riplinger and F. Neese, *J. Chem. Phys.*, 2013, **138**, 034106.
14. D. G. Liakos, Y. Guo and F. Neese, *J. Phys. Chem. A*, 2019, **124**, 90-100.
15. D. E. Woon and T. H. Dunning Jr, *J. Chem. Phys.*, 1993, **98**, 1358-1371.
16. T. H. Dunning Jr, *J. Chem. Phys.*, 1989, **90**, 1007-1023.
17. F. Neese, *Wiley Interdiscip. Rev. Comput. Mol. Sci.*, 2012, **2**, 73-78.
18. M. J. Frisch, G. W. Trucks, H. B. Schlegel, G. E. Scuseria, M. A. Robb, J. R. Cheeseman, G. Scalmani, V. Barone, G. A. Petersson, H. Nakatsuji, X. Li, M. Caricato, A. V. Marenich, J. Bloino, B. G. Janesko, R. Gomperts, B. Mennucci, H. P. Hratchian, J. V. Ortiz, A. F. Izmaylov, J. L. Sonnenberg, Williams, F. Ding, F. Lipparini, F. Egidi, J. Goings, B. Peng, A. Petrone, T. Henderson, D. Ranasinghe, V. G. Zakrzewski, J. Gao, N. Rega, G. Zheng, W. Liang, M. Hada, M. Ehara, K. Toyota, R. Fukuda, J. Hasegawa, M. Ishida, T. Nakajima, Y. Honda, O. Kitao, H. Nakai, T. Vreven, K. Throssell, J. A. Montgomery Jr., J. E. Peralta, F. Ogliaro, M. J. Bearpark, J. J. Heyd, E. N. Brothers, K. N. Kudin, V. N. Staroverov, T. A. Keith, R. Kobayashi, J. Normand, K. Raghavachari, A. P. Rendell, J. C. Burant, S. S. Iyengar, J. Tomasi, M. Cossi, J. M. Millam, M. Klene, C. Adamo, R. Cammi, J. W. Ochterski, R. L. Martin, K. Morokuma, O. Farkas, J. B. Foresman and D. J. Fox, *Journal*, 2016.
19. T. Yanai, D. P. Tew and N. C. Handy, *Chem. Phys. Lett.*, 2004, **393**, 51-57.
20. E. D. Glendening, C. R. Landis and F. Weinhold, *J. Comput. Chem.*, 2013, **34**, 1429-1437.
21. F. Weinhold, *J. Comput. Chem.*, 2012, **33**, 2363-2379.
22. E. Glendening, C. Landis and F. Weinhold, *Wiley Interdiscip. Rev. Comput. Mol. Sci.*, 2012, **2**, 1-42.
23. A. E. Reed, L. A. Curtiss and F. Weinhold, *Chem. Rev.*, 1988, **88**, 899-926.

24. K. Wolinski, J. F. Hinton and P. Pulay, *J. Am. Chem. Soc.*, 1990, **112**, 8251-8260.
25. R. Ditchfield, *Mol. Phys.*, 1974, **27**, 789-807.
26. A. V. Marenich, C. J. Cramer and D. G. Truhlar, *The Journal of Physical Chemistry B*, 2009, **113**, 6378-6396.
27. H. S. Yu, X. He, S. L. Li and D. G. Truhlar, *Chem. Sci.*, 2016, **7**, 5032-5051.
28. F. Weigend and R. Ahlrichs, *Phys. Chem. Chem. Phys.*, 2005, **7**, 3297-3305.
29. R. J. Mayer, N. Hampel and A. R. Ofial, *Chem. Eur. J.*, 2021, **27**, 4070-4080.
30. C. Angeli, R. Cimiraglia and J.-P. Malrieu, *J. Chem. Phys.*, 2002, **117**, 9138-9153.
